# Natural djurleite with refined composition Cu_61.39_S_32_ revealing disorder of some Cu sites

**DOI:** 10.1107/S2414314622006940

**Published:** 2022-07-12

**Authors:** Yawei Zhou, Changzeng Fan, Bin Wen, Lifeng Zhang

**Affiliations:** a State Key Laboratory of Metastable Materials Science and Technology, Yanshan University, Qinhuangdao 066004, People’s Republic of China; Vienna University of Technology, Austria

**Keywords:** crystal structure, Cu_61.39_S_32_, twin, disorder, redetermination

## Abstract

A djurleite crystal was discovered from a natural sample originally labelled as chalcocite. The djurleite crystal under investigation has a refined composition of Cu_61.39_S_32_, thus revealing a Cu deficiency compared to the originally reported Cu_62_S_32_ phase [Evans (1979). *Z. Kristallogr.* **150**, 299–320], where all atomic sites are reported to be fully occupied. In addition, the current refinement revealed a splitting of eight of the Cu sites into pairs.

## Abstract 2

The djurleite phase with composition Cu_61.39_S_32_ was refined from a natural twinned specimen and shows disorder of some of its Cu sites.

## Graphical abstract

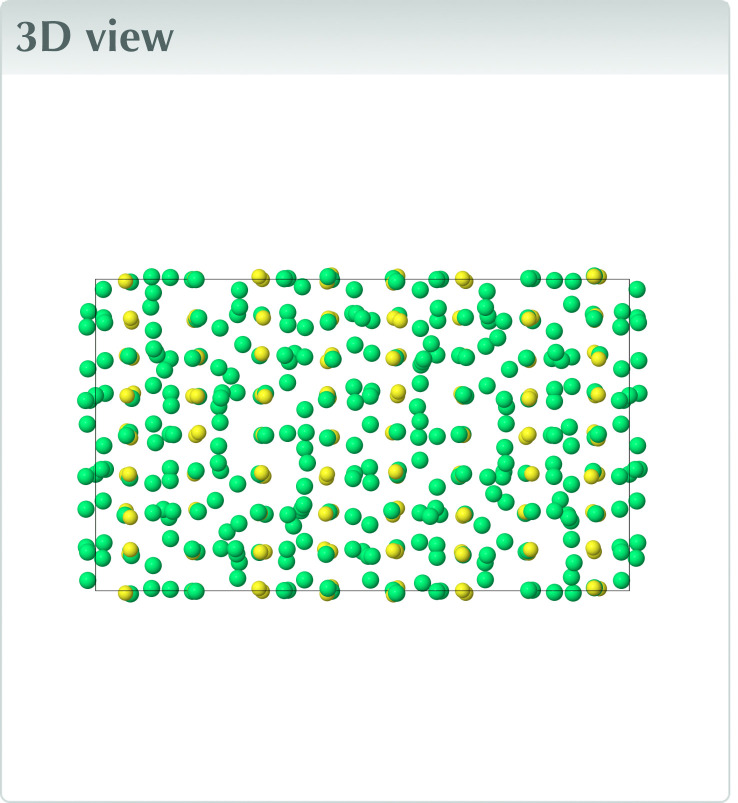

## Structure description

The Cu–S system has been the subject of structural research for nearly one century. Several well-defined compounds and their crystal structures have been reported, with high-chalcocite (Buerger & Wuensch, 1963 ▸), low-chalcocite and djurleite (Evans, 1979*a* ▸,*b* ▸) being the most prominent ones. Low-chalcocite and djurleite are difficult to distinguish, thus many samples labelled chalcocite represent in fact djurleite, or an intimately inter­grown mixture of low-chalcocite and djurleite (Evans, 1981 ▸). The existence of djurleite was not suspected until it was discovered and suggested to be of ortho­rhom­bic symmetry by Djurle (1958 ▸). This phase was later assigned as an independent mineral species (Roseboom, 1962 ▸; Morimoto, 1962 ▸). Further studies of this mineral revealed that the previously supposed space group of *Pmmm* is affected by systematic twinning and that djurleite actually crystallizes in the monoclinic space group *P*2_1_/*n* (Takeda *et al.*, 1967 ▸; Evans, 1979*a* ▸,*b* ▸). The final determination of the crystal structures of low-chalcocite and djurleite was accomplished by Evans (1979*a* ▸). Low-chalcocite from Bristol, Connecticut, crystallizes in space group *P*2_1_/*c* and contains 48 formula units of Cu_2_S [*a* = 15.246 (4), *b* = 11.884 (2), *c* = 13.494 (3) Å, *β* = 116.35 (1)°] while djurleite from the Ozark Lead Co. mine at Sweetwater, Missouri, contains eight formula units of Cu_31_S_16_ in space group *P*2_1_/*n* [*a* = 26.897 (6), *b*= 15.745 (3), *c*= 13.565 (3) Å, *β* = 90.13 (3)°; Evans, 1979*a* ▸,*b* ▸]. All atoms in the above structure models were assumed to be fully occupied. However, there are some studies based on HRTEM revealing that there are vacancies of Cu in natural metadjurleite (Xu *et al.*, 1991 ▸) or low-djurleite (Sun & Xue, 2001 ▸), but without a refined structure model. There are also some studies on synthetic copper-deficient copper sulfides, either considered as monoclinic djurleite (Yoon *et al.*, 2015 ▸), or as cubic Cu_2_S (Zhou *et al.*, 2016 ▸.; Zhang *et al.*, 2021 ▸).

Since the crystal structure of djurleite has been discussed in detail in the original description (Evans, 1979*a* ▸), here only the main differences are emphasized. For the present study, a crystal from a natural sample was used, revealing a refined composition of Cu_61.39_S_32_. In comparison with the original Cu_31_S_16_ model where all sites are ordered and fully occupied, eight Cu sites are split over two positions and one Cu site shows partial occupancy. The crystal under investigation used for the present study was twinned whereas that used for the original study was not reported to be twinned. The reported temperature of formation for the latter crystal was below 370 K.

**Figure 1:**
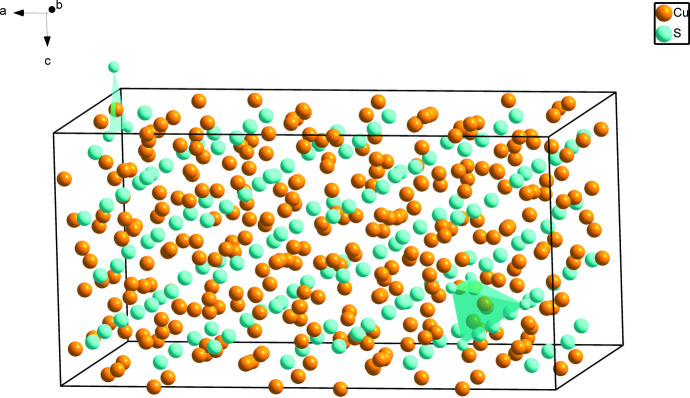
The unit cell of Cu_61.39_S_32_, with sites Cu1 and Cu48 displayed with their different coordination environments as polyhedra.

**Figure 2:**
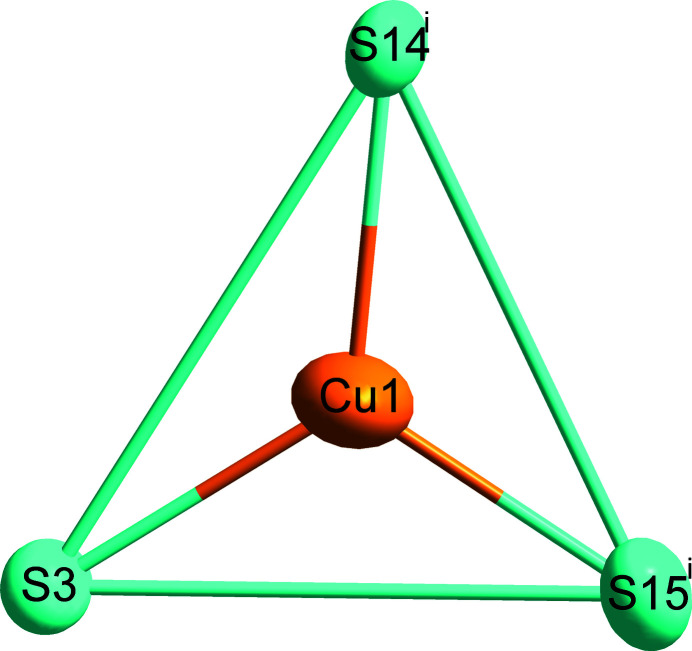
The environment of Cu1. Displacement ellipsoids are drawn at the 90% probability level. [Symmetry code: (i) *x*, *y*, *z* − 1.]

**Figure 3:**
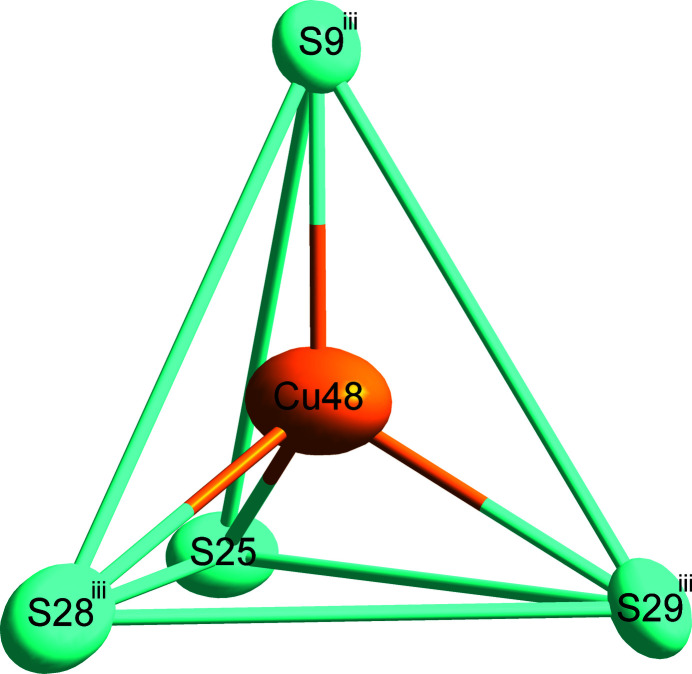
The environment of Cu48. Displacement ellipsoids are drawn at the 90% probability level. [Symmetry code: (iii) *x*, *y* − 1, *z*.]

Fig. 1 ▸ shows the overall atomic distribution of Cu_61.39_S_32_ in the unit cell. For simplicity, two different coordination polyhedra around Cu sites are highlighted, with a criterion of the Cu—S distances being less than 3.0 Å. Representative for the principal environment of Cu sites, Cu1 shows a triangular coordination by three S atoms (Fig. 2 ▸). Another environment of Cu sites is represented by Cu48, where the surrounding four S atoms form a distorted tetra­hedron, as shown in Fig. 3 ▸. The low-occupancy Cu12 site has the same environment as the Cu1 atom. The eight pairs of split Cu atoms have the same environment as Cu48, except the pairs Cu29*A*/*B*, Cu45*A*/*B* and Cu62*A*/*B*, which have the same environment as the Cu1 atom.

## Synthesis and crystallization

Natural samples designated as chalcocite were purchased from Honghu Minerals (Hubei Province, China), Alibaba Taobao Co. Suitable single-crystal fragments were broken from a larger sample and glued on glass fibers for single-crystal X-ray diffraction experiments. Energy-dispersive X-ray spectroscopy measurements did not indicate the presence of elements other than Cu and S (see supporting information).

## Refinement

Crystal data, data collection and structure refinement details are summarized in Table 1 ▸. Starting atomic coordinates and labels were adapted from the original structure investigation (Evans, 1979*a* ▸). The crystal under investigation was twinned by pseudo-merohedry, revealing a twin ratio of 0.92:0.08. Eight pairs of split Cu sites were assigned, in all cases assuming full occupancy using the same anisotropic displacement parameters (EADP) for each pair: Cu29*A*/Cu29*B* [occupancy ratio 0.808 (12)/0.192 (12)], Cu36*A*/Cu36*B* [0.815 (15)/0.185 (15)], Cu45*A*/Cu45*B* [0.860 (18)/0.140 (18)], Cu47*A*/Cu47*B* [0.561 (15)/0.439 (15)], Cu51*A*/Cu51*B* [0.580 (9)/0.480 (9)], Cu54*A*/Cu54*B* [0.770 (6)/0.230 (6)], Cu60*A*/Cu60*B* [0.687 (10)/0.313 (10)], and Cu62*A*/Cu62*B* [0.804 (6)/0.196 (6)]. In addition, the Cu12 site shows an occupancy less than 1, with a refined site occupation of 0.385 (9). The maximum residual electron density in the final difference-Fourier map is located 1.665 Å from atom Cu12 and the minimum electron density is located 0.487 Å from atom Cu51*B*.

## Supplementary Material

Crystal structure: contains datablock(s) I. DOI: 10.1107/S2414314622006940/wm4164sup1.cif

Structure factors: contains datablock(s) I. DOI: 10.1107/S2414314622006940/wm4164Isup4.hkl

Supplementary information (EDX analysis). DOI: 10.1107/S2414314622006940/wm4164sup3.docx

CCDC reference: 2184537

Additional supporting information:  crystallographic information; 3D view; checkCIF report

## Figures and Tables

**Table 1:** Experimental details

Crystal data	
Chemical formula	Cu_61.39_S_32_
*M*_r_	4926.40
Crystal system, space group	Monoclinic, *P*2_1_/*n*
Temperature (K)	296
*a*, *b*, *c* (Å)	26.850 (1), 15.6862 (6), 13.5175 (6)
β (°)	90.062 (2)
*V* (Å^3^)	5693.2 (4)
*Z*	4
Radiation type	Mo *K*α
μ (mm^−1^)	23.54
Crystal size (mm)	0.11 × 0.07 × 0.07

Data collection	
Diffractometer	Bruker D8 Venture Photon 100 CMOS
Absorption correction	Multi-scan (*SADABS*; Krause *et al.*, 2015 ▸)
*T*_min_, *T*_max_	0.526, 0.745
No. of measured, independent and observed [*I* > 2σ(*I*)] reflections	19946, 10098, 7077
*R*_int_	0.062
(sin θ/λ)_max_ (Å^−1^)	0.597

Refinement	
*R*[*F* ^2^ > 2σ(*F* ^2^)], *wR*(*F* ^2^), *S*	0.067, 0.132, 0.95
No. of reflections	10098
No. of parameters	881
Δρ_max_, Δρ_min_ (e Å^−3^)	2.03, −1.59

Computer programs: *APEX3* and *SAINT* (Bruker, 2015 ▸), *SHELXT* (Sheldrick, 2015*a* ▸), *SHELXL* (Sheldrick, 2015*b* ▸), *DIAMOND* (Brandenburg & Putz, 2017 ▸) and *publCIF* (Westrip, 2010 ▸).

## full crystallographic data

### Crystal data

**Table:**

Cu_61.39_S_32_	*F*(000) = 9169
*M**_r_* = 4926.40	*D*_x_ = 5.748 Mg m^−^^3^
Monoclinic, *P*2_1_/*n*	Mo *K*α radiation, λ = 0.71073 Å
*a* = 26.850 (1) Å	Cell parameters from 9872 reflections
*b* = 15.6862 (6) Å	θ = 2.6–25.0°
*c* = 13.5175 (6) Å	µ = 23.54 mm^−^^1^
β = 90.062 (2)°	*T* = 296 K
*V* = 5693.2 (4) Å^3^	Fragment, dark grey
*Z* = 4	0.11 × 0.07 × 0.07 mm

### Data collection

**Table:**

Bruker D8 Venture Photon 100 CMOS diffractometer	7077 reflections with *I* > 2σ(*I*)
phi and ω scans	*R*_int_ = 0.062
Absorption correction: multi-scan (SADABS; Krause *et al.*, 2015)	θ_max_ = 25.1°, θ_min_ = 2.1°
*T*_min_ = 0.526, *T*_max_ = 0.745	*h* = 0→31
19946 measured reflections	*k* = −18→18
10098 independent reflections	*l* = −16→16

### Refinement

**Table:**

Refinement on *F*^2^	881 parameters
Least-squares matrix: full	0 restraints
*R*[*F*^2^ > 2σ(*F*^2^)] = 0.067	*w* = 1/[σ^2^(*F*_o_^2^) + (0.0431*P*)^2^ + 226.7933*P*] where *P* = (*F*_o_^2^ + 2*F*_c_^2^)/3
*wR*(*F*^2^) = 0.132	(Δ/σ)_max_ = 0.050
*S* = 0.95	Δρ_max_ = 2.03 e Å^−^^3^
10098 reflections	Δρ_min_ = −1.59 e Å^−^^3^

### Special details

**Table:**

Geometry. All esds (except the esd in the dihedral angle between two l.s. planes) are estimated using the full covariance matrix. The cell esds are taken into account individually in the estimation of esds in distances, angles and torsion angles; correlations between esds in cell parameters are only used when they are defined by crystal symmetry. An approximate (isotropic) treatment of cell esds is used for estimating esds involving l.s. planes.
Refinement. Refined as a 2-component inversion twin.

### Fractional atomic coordinates and isotropic or equivalent isotropic displacement parameters (Å^2^)

**Table:**

	*x*	*y*	*z*	*U*_iso_*/*U*_eq_	Occ. (<1)
Cu1	0.05802 (8)	0.49828 (14)	0.00493 (17)	0.0241 (5)	
Cu2	0.06237 (8)	0.75278 (15)	0.00218 (17)	0.0254 (5)	
Cu3	0.06563 (8)	0.12723 (14)	0.24690 (16)	0.0218 (5)	
Cu4	0.06530 (9)	0.99009 (15)	0.49282 (17)	0.0283 (6)	
Cu5	0.05524 (8)	0.24246 (14)	0.50056 (17)	0.0246 (5)	
Cu6	0.06408 (8)	0.50787 (14)	0.49479 (17)	0.0258 (6)	
Cu7	0.06740 (8)	0.11292 (14)	0.75840 (16)	0.0205 (5)	
Cu8	0.06409 (9)	0.36157 (15)	0.74230 (18)	0.0291 (6)	
Cu9	0.06786 (8)	0.61680 (14)	0.75346 (16)	0.0201 (5)	
Cu10	0.18823 (7)	0.99835 (14)	0.99856 (16)	0.0191 (5)	
Cu11	0.19240 (8)	0.25413 (14)	0.99740 (17)	0.0237 (5)	
Cu12	0.1821 (2)	0.7453 (4)	0.9908 (5)	0.032 (3)	0.385 (9)
Cu13	0.18979 (10)	0.12498 (17)	0.24019 (19)	0.0382 (6)	
Cu14	0.19304 (8)	0.62310 (15)	0.24707 (17)	0.0262 (5)	
Cu15	0.18958 (7)	0.87104 (14)	0.24350 (16)	0.0200 (5)	
Cu16	0.18134 (8)	0.99834 (16)	0.48732 (18)	0.0297 (6)	
Cu17	0.19465 (8)	0.75158 (14)	0.49793 (17)	0.0224 (5)	
Cu18	0.18129 (8)	0.13355 (14)	0.75600 (17)	0.0245 (5)	
Cu19	0.18859 (8)	0.38481 (14)	0.74382 (17)	0.0251 (5)	
Cu20	0.19348 (9)	0.87522 (15)	0.74492 (18)	0.0295 (6)	
Cu21	0.01578 (7)	0.15373 (14)	0.06607 (16)	0.0197 (5)	
Cu22	0.01827 (8)	0.38946 (14)	0.14172 (17)	0.0230 (5)	
Cu23	0.01800 (8)	0.63341 (15)	0.12577 (19)	0.0272 (6)	
Cu24	0.01467 (7)	0.87550 (14)	0.12492 (17)	0.0203 (5)	
Cu25	0.01418 (8)	0.28830 (14)	0.30698 (17)	0.0213 (5)	
Cu26	0.01536 (8)	0.46557 (15)	0.32197 (18)	0.0267 (6)	
Cu27	0.01788 (8)	0.73844 (18)	0.3340 (2)	0.0370 (7)	
Cu28	0.01314 (8)	0.10450 (16)	0.5913 (2)	0.0355 (7)	
Cu29A	0.01089 (14)	0.3892 (2)	0.5656 (4)	0.0255 (12)	0.808 (12)
Cu29B	0.0002 (6)	0.3741 (11)	0.5212 (18)	0.0255 (12)	0.192 (12)
Cu30	0.01741 (8)	0.85988 (15)	0.6313 (2)	0.0304 (6)	
Cu31	0.01468 (7)	0.96688 (13)	0.81303 (16)	0.0189 (5)	
Cu32	0.10780 (7)	0.08082 (14)	0.07141 (16)	0.0186 (5)	
Cu33	0.10697 (7)	0.34458 (15)	0.06377 (17)	0.0243 (5)	
Cu34	0.10323 (7)	0.90973 (14)	0.04937 (16)	0.0191 (5)	
Cu35	0.10851 (8)	0.53484 (16)	0.31335 (17)	0.0257 (6)	
Cu36A	0.11275 (12)	0.7659 (3)	0.3107 (6)	0.0371 (14)	0.815 (15)
Cu36B	0.1151 (6)	0.7567 (14)	0.361 (2)	0.0371 (14)	0.185 (15)
Cu37	0.10822 (8)	0.95624 (16)	0.31530 (17)	0.0267 (6)	
Cu38	0.10853 (7)	0.65868 (14)	0.56067 (16)	0.0202 (5)	
Cu39	0.10823 (8)	0.83533 (16)	0.55721 (19)	0.0316 (6)	
Cu40	0.10540 (8)	0.00731 (14)	0.88738 (17)	0.0207 (5)	
Cu41	0.10783 (8)	0.24996 (15)	0.87892 (19)	0.0275 (6)	
Cu42	0.11246 (9)	0.47519 (18)	0.8395 (2)	0.0447 (7)	
Cu43	0.10950 (8)	0.77570 (15)	0.81362 (17)	0.0237 (5)	
Cu44	0.13962 (7)	0.50212 (14)	0.12734 (17)	0.0239 (5)	
Cu45A	0.13951 (17)	0.7440 (5)	0.1307 (4)	0.0431 (15)	0.860 (18)
Cu45B	0.1290 (10)	0.708 (3)	0.149 (3)	0.0431 (15)	0.140 (18)
Cu46	0.14045 (9)	0.16731 (19)	0.4294 (2)	0.0438 (7)	
Cu47A	0.1400 (4)	0.3927 (8)	0.4268 (10)	0.0390 (19)	0.561 (15)
Cu47B	0.1398 (5)	0.3538 (9)	0.4288 (13)	0.0390 (19)	0.439 (15)
Cu48	0.14012 (8)	0.00475 (15)	0.66288 (19)	0.0286 (6)	
Cu49	0.14479 (8)	0.25517 (15)	0.6138 (2)	0.0314 (6)	
Cu50	0.14618 (8)	0.50105 (15)	0.6134 (2)	0.0305 (6)	
Cu51A	0.1412 (2)	0.6334 (6)	0.9080 (6)	0.0414 (16)	0.580 (9)
Cu51B	0.1421 (4)	0.5940 (8)	0.9353 (8)	0.0414 (16)	0.420 (9)
Cu52	0.22434 (8)	0.28965 (16)	0.18953 (18)	0.0291 (6)	
Cu53	0.23055 (8)	0.46425 (15)	0.20081 (18)	0.0261 (6)	
Cu54A	0.23507 (12)	0.1357 (3)	0.4194 (3)	0.0525 (13)	0.770 (6)
Cu54B	0.2463 (4)	0.1889 (11)	0.3907 (12)	0.0525 (13)	0.230 (6)
Cu55	0.23404 (9)	0.3859 (2)	0.3949 (3)	0.0576 (9)	
Cu56	0.23085 (8)	0.59121 (16)	0.43753 (17)	0.0270 (6)	
Cu57	0.23338 (8)	0.54026 (15)	0.69488 (18)	0.0274 (6)	
Cu58	0.23099 (8)	0.71556 (15)	0.68504 (18)	0.0245 (5)	
Cu59	0.23013 (8)	0.41014 (17)	0.93999 (19)	0.0343 (6)	
Cu60A	0.23741 (15)	0.6351 (3)	0.8782 (5)	0.0335 (14)	0.687 (10)
Cu60B	0.2353 (4)	0.6181 (8)	0.9294 (11)	0.0335 (14)	0.313 (10)
Cu61	0.23358 (10)	0.8427 (2)	0.9271 (3)	0.0686 (11)	
Cu62A	0.12704 (11)	0.2611 (2)	0.2464 (3)	0.0336 (11)	0.804 (6)
Cu62B	0.1372 (5)	0.2355 (9)	0.1835 (14)	0.0336 (11)	0.196 (6)
S1	0.06574 (14)	0.9910 (3)	0.1732 (3)	0.0126 (9)	
S2	0.05702 (14)	0.2565 (3)	0.1614 (3)	0.0136 (9)	
S3	0.05843 (14)	0.5128 (3)	0.1762 (3)	0.0132 (9)	
S4	0.05761 (14)	0.7543 (3)	0.1732 (3)	0.0148 (9)	
S5	0.06260 (15)	0.1181 (3)	0.4159 (3)	0.0160 (10)	
S6	0.05801 (14)	0.3714 (3)	0.4213 (3)	0.0148 (9)	
S7	0.07091 (14)	0.6340 (3)	0.4117 (3)	0.0130 (9)	
S8	0.06749 (15)	0.8621 (3)	0.4134 (3)	0.0175 (10)	
S9	0.05634 (14)	0.9928 (3)	0.6624 (3)	0.0127 (9)	
S10	0.06507 (14)	0.2353 (3)	0.6692 (3)	0.0156 (10)	
S11	0.06584 (15)	0.4910 (3)	0.6667 (3)	0.0166 (10)	
S12	0.06836 (14)	0.7467 (3)	0.6704 (3)	0.0142 (9)	
S13	0.06560 (14)	0.1321 (3)	0.9276 (3)	0.0106 (9)	
S14	0.06372 (14)	0.3742 (3)	0.9150 (3)	0.0132 (9)	
S15	0.05939 (15)	0.6254 (3)	0.9224 (3)	0.0191 (10)	
S16	0.06580 (14)	0.8742 (3)	0.9023 (3)	0.0119 (9)	
S17	0.18785 (15)	0.1288 (3)	0.0752 (3)	0.0164 (10)	
S18	0.18758 (14)	0.3836 (3)	0.0823 (3)	0.0162 (10)	
S19	0.18235 (15)	0.6249 (3)	0.0769 (3)	0.0157 (9)	
S20	0.18465 (15)	0.8681 (3)	0.0736 (3)	0.0152 (9)	
S21	0.18876 (15)	0.0012 (3)	0.3234 (4)	0.0192 (10)	
S22	0.19085 (18)	0.2561 (3)	0.3414 (3)	0.0248 (11)	
S23	0.18798 (15)	0.4987 (3)	0.3416 (3)	0.0163 (10)	
S24	0.19465 (15)	0.7475 (3)	0.3320 (3)	0.0163 (10)	
S25	0.18212 (14)	0.1233 (3)	0.5801 (3)	0.0152 (10)	
S26	0.18557 (15)	0.3751 (3)	0.5748 (3)	0.0194 (10)	
S27	0.19055 (14)	0.6241 (3)	0.5811 (3)	0.0136 (9)	
S28	0.18839 (15)	0.8785 (3)	0.5788 (3)	0.0198 (10)	
S29	0.18594 (15)	1.0002 (3)	0.8265 (3)	0.0151 (10)	
S30	0.18823 (15)	0.2604 (3)	0.8296 (3)	0.0171 (10)	
S31	0.19344 (15)	0.5075 (3)	0.8398 (3)	0.0151 (10)	
S32	0.19131 (15)	0.7493 (3)	0.8303 (3)	0.0161 (10)	

### Atomic displacement parameters (Å^2^)

**Table:**

	*U*^11^	*U*^22^	*U*^33^	*U*^12^	*U*^13^	*U*^23^
Cu1	0.0320 (13)	0.0188 (13)	0.0214 (14)	0.0006 (10)	−0.0004 (10)	−0.0054 (10)
Cu2	0.0297 (12)	0.0247 (14)	0.0217 (14)	−0.0014 (10)	0.0000 (10)	0.0059 (11)
Cu3	0.0284 (12)	0.0182 (12)	0.0189 (13)	−0.0031 (10)	0.0024 (9)	0.0010 (10)
Cu4	0.0491 (15)	0.0183 (13)	0.0176 (14)	0.0029 (11)	0.0057 (11)	−0.0028 (11)
Cu5	0.0326 (13)	0.0165 (12)	0.0248 (14)	0.0034 (10)	0.0007 (10)	0.0010 (11)
Cu6	0.0338 (13)	0.0209 (13)	0.0228 (15)	−0.0069 (10)	−0.0056 (10)	0.0069 (11)
Cu7	0.0321 (12)	0.0162 (12)	0.0132 (12)	−0.0013 (9)	−0.0001 (9)	−0.0005 (10)
Cu8	0.0454 (14)	0.0197 (13)	0.0221 (14)	0.0005 (11)	−0.0026 (11)	−0.0012 (11)
Cu9	0.0237 (11)	0.0160 (12)	0.0206 (13)	−0.0003 (9)	0.0016 (9)	−0.0004 (10)
Cu10	0.0210 (11)	0.0154 (12)	0.0208 (13)	0.0001 (9)	0.0001 (9)	−0.0021 (10)
Cu11	0.0267 (12)	0.0202 (13)	0.0240 (14)	−0.0028 (10)	−0.0047 (10)	−0.0005 (11)
Cu12	0.047 (4)	0.027 (4)	0.021 (4)	−0.008 (3)	0.006 (3)	0.005 (3)
Cu13	0.0548 (16)	0.0343 (16)	0.0256 (15)	−0.0014 (13)	−0.0020 (12)	0.0046 (13)
Cu14	0.0337 (13)	0.0229 (13)	0.0220 (14)	−0.0049 (10)	0.0004 (10)	0.0007 (11)
Cu15	0.0220 (11)	0.0186 (12)	0.0194 (13)	−0.0004 (9)	−0.0019 (9)	−0.0010 (10)
Cu16	0.0369 (14)	0.0256 (14)	0.0265 (15)	0.0024 (11)	0.0038 (11)	0.0022 (11)
Cu17	0.0287 (12)	0.0199 (13)	0.0185 (13)	0.0022 (10)	0.0026 (9)	0.0012 (10)
Cu18	0.0296 (12)	0.0163 (12)	0.0276 (14)	−0.0003 (10)	−0.0044 (10)	−0.0030 (11)
Cu19	0.0365 (13)	0.0198 (13)	0.0189 (13)	0.0027 (10)	−0.0036 (10)	−0.0012 (10)
Cu20	0.0367 (13)	0.0211 (13)	0.0308 (15)	0.0040 (10)	0.0002 (11)	−0.0037 (11)
Cu21	0.0148 (11)	0.0248 (13)	0.0195 (13)	−0.0017 (9)	0.0026 (9)	0.0007 (10)
Cu22	0.0206 (11)	0.0188 (13)	0.0295 (14)	−0.0036 (9)	0.0038 (9)	−0.0018 (11)
Cu23	0.0206 (12)	0.0206 (13)	0.0406 (16)	0.0002 (10)	−0.0021 (10)	0.0032 (12)
Cu24	0.0120 (10)	0.0219 (12)	0.0269 (13)	−0.0003 (9)	0.0010 (9)	−0.0026 (11)
Cu25	0.0211 (12)	0.0203 (12)	0.0225 (13)	−0.0017 (9)	−0.0002 (9)	0.0012 (10)
Cu26	0.0225 (12)	0.0279 (14)	0.0297 (15)	0.0008 (10)	−0.0066 (10)	0.0017 (12)
Cu27	0.0157 (12)	0.0515 (17)	0.0438 (17)	−0.0020 (11)	−0.0002 (11)	0.0080 (14)
Cu28	0.0247 (13)	0.0284 (14)	0.0535 (19)	0.0084 (11)	−0.0077 (12)	−0.0010 (13)
Cu29A	0.0198 (17)	0.0252 (18)	0.032 (3)	0.0002 (13)	0.0107 (18)	0.0028 (19)
Cu29B	0.0198 (17)	0.0252 (18)	0.032 (3)	0.0002 (13)	0.0107 (18)	0.0028 (19)
Cu30	0.0167 (11)	0.0204 (13)	0.0541 (17)	0.0026 (10)	0.0029 (11)	0.0101 (12)
Cu31	0.0148 (11)	0.0180 (12)	0.0239 (13)	0.0017 (9)	0.0001 (9)	0.0039 (10)
Cu32	0.0194 (11)	0.0186 (12)	0.0180 (13)	0.0003 (9)	0.0050 (9)	−0.0018 (10)
Cu33	0.0164 (11)	0.0279 (14)	0.0284 (14)	−0.0017 (9)	0.0007 (10)	0.0006 (11)
Cu34	0.0176 (11)	0.0210 (12)	0.0187 (13)	−0.0016 (9)	0.0010 (9)	−0.0039 (10)
Cu35	0.0175 (11)	0.0396 (15)	0.0201 (13)	−0.0001 (10)	−0.0010 (9)	−0.0055 (11)
Cu36A	0.0159 (14)	0.057 (2)	0.039 (4)	0.0053 (13)	−0.0004 (18)	−0.001 (2)
Cu36B	0.0159 (14)	0.057 (2)	0.039 (4)	0.0053 (13)	−0.0004 (18)	−0.001 (2)
Cu37	0.0210 (12)	0.0362 (15)	0.0228 (14)	−0.0022 (10)	0.0009 (10)	0.0028 (12)
Cu38	0.0208 (11)	0.0206 (12)	0.0191 (13)	0.0001 (9)	−0.0006 (9)	−0.0037 (10)
Cu39	0.0200 (12)	0.0378 (15)	0.0371 (16)	0.0023 (10)	0.0014 (11)	0.0151 (12)
Cu40	0.0213 (11)	0.0171 (12)	0.0237 (14)	0.0024 (9)	−0.0047 (9)	0.0003 (10)
Cu41	0.0197 (12)	0.0229 (13)	0.0397 (16)	−0.0019 (10)	0.0021 (10)	0.0037 (12)
Cu42	0.0240 (13)	0.0472 (18)	0.063 (2)	−0.0169 (12)	0.0060 (13)	−0.0022 (15)
Cu43	0.0173 (11)	0.0266 (13)	0.0271 (14)	0.0046 (10)	−0.0021 (10)	−0.0081 (11)
Cu44	0.0177 (11)	0.0253 (13)	0.0286 (15)	0.0041 (10)	0.0003 (10)	−0.0037 (11)
Cu45A	0.0188 (18)	0.032 (4)	0.078 (3)	0.002 (2)	−0.0005 (17)	−0.019 (2)
Cu45B	0.0188 (18)	0.032 (4)	0.078 (3)	0.002 (2)	−0.0005 (17)	−0.019 (2)
Cu46	0.0242 (14)	0.0532 (19)	0.0541 (19)	−0.0117 (12)	0.0112 (12)	0.0015 (15)
Cu47A	0.0180 (14)	0.052 (6)	0.047 (2)	−0.001 (5)	−0.0026 (13)	0.012 (6)
Cu47B	0.0180 (14)	0.052 (6)	0.047 (2)	−0.001 (5)	−0.0026 (13)	0.012 (6)
Cu48	0.0189 (12)	0.0289 (14)	0.0380 (16)	−0.0011 (10)	0.0005 (10)	−0.0056 (12)
Cu49	0.0257 (13)	0.0228 (14)	0.0459 (17)	−0.0041 (10)	0.0010 (11)	0.0037 (12)
Cu50	0.0225 (12)	0.0217 (14)	0.0474 (17)	−0.0054 (10)	−0.0065 (11)	0.0078 (12)
Cu51A	0.0183 (15)	0.067 (6)	0.039 (4)	0.000 (3)	0.001 (2)	0.010 (3)
Cu51B	0.0183 (15)	0.067 (6)	0.039 (4)	0.000 (3)	0.001 (2)	0.010 (3)
Cu52	0.0261 (13)	0.0328 (15)	0.0285 (15)	0.0008 (10)	0.0046 (10)	0.0031 (12)
Cu53	0.0184 (12)	0.0289 (14)	0.0308 (15)	0.0022 (10)	−0.0019 (10)	−0.0042 (12)
Cu54A	0.0168 (17)	0.074 (4)	0.066 (3)	0.0043 (19)	−0.0020 (16)	0.019 (3)
Cu54B	0.0168 (17)	0.074 (4)	0.066 (3)	0.0043 (19)	−0.0020 (16)	0.019 (3)
Cu55	0.0190 (14)	0.068 (2)	0.086 (2)	0.0124 (13)	0.0022 (14)	0.0393 (19)
Cu56	0.0181 (12)	0.0362 (15)	0.0267 (14)	−0.0022 (10)	0.0018 (10)	−0.0042 (12)
Cu57	0.0207 (12)	0.0295 (14)	0.0319 (15)	−0.0015 (10)	−0.0047 (10)	0.0087 (12)
Cu58	0.0210 (12)	0.0254 (13)	0.0271 (14)	−0.0043 (10)	−0.0023 (10)	−0.0033 (11)
Cu59	0.0249 (13)	0.0423 (16)	0.0357 (16)	0.0023 (11)	0.0079 (11)	0.0120 (13)
Cu60A	0.0173 (15)	0.033 (2)	0.050 (4)	0.0003 (15)	−0.004 (2)	0.000 (3)
Cu60B	0.0173 (15)	0.033 (2)	0.050 (4)	0.0003 (15)	−0.004 (2)	0.000 (3)
Cu61	0.0277 (16)	0.101 (3)	0.077 (2)	0.0009 (16)	−0.0182 (15)	−0.052 (2)
Cu62A	0.0234 (16)	0.0261 (19)	0.051 (3)	−0.0025 (13)	−0.0185 (16)	0.0017 (18)
Cu62B	0.0234 (16)	0.0261 (19)	0.051 (3)	−0.0025 (13)	−0.0185 (16)	0.0017 (18)
S1	0.009 (2)	0.014 (2)	0.015 (2)	0.0002 (16)	0.0011 (16)	−0.0002 (19)
S2	0.013 (2)	0.008 (2)	0.020 (3)	−0.0007 (16)	−0.0007 (17)	0.0054 (19)
S3	0.012 (2)	0.014 (2)	0.014 (2)	−0.0026 (17)	−0.0036 (17)	−0.0003 (19)
S4	0.015 (2)	0.017 (2)	0.013 (2)	0.0006 (18)	−0.0006 (17)	0.0037 (19)
S5	0.018 (2)	0.014 (2)	0.015 (2)	−0.0027 (17)	−0.0028 (17)	0.0001 (19)
S6	0.014 (2)	0.015 (2)	0.016 (2)	−0.0014 (18)	−0.0007 (17)	0.004 (2)
S7	0.015 (2)	0.013 (2)	0.011 (2)	−0.0028 (17)	−0.0038 (16)	0.0035 (19)
S8	0.018 (2)	0.015 (2)	0.019 (3)	−0.0024 (18)	−0.0019 (18)	−0.006 (2)
S9	0.014 (2)	0.013 (2)	0.011 (2)	0.0000 (17)	0.0012 (17)	0.0030 (18)
S10	0.011 (2)	0.017 (2)	0.018 (3)	0.0006 (17)	0.0014 (17)	0.001 (2)
S11	0.016 (2)	0.015 (2)	0.019 (3)	−0.0028 (18)	0.0025 (18)	−0.002 (2)
S12	0.010 (2)	0.016 (2)	0.016 (3)	0.0017 (17)	0.0024 (16)	0.0014 (19)
S13	0.010 (2)	0.014 (2)	0.007 (2)	−0.0007 (17)	−0.0004 (15)	−0.0001 (19)
S14	0.012 (2)	0.014 (2)	0.014 (2)	0.0017 (17)	−0.0018 (16)	0.0031 (19)
S15	0.020 (2)	0.023 (3)	0.014 (2)	0.0034 (19)	−0.0006 (18)	0.002 (2)
S16	0.010 (2)	0.014 (2)	0.012 (2)	−0.0001 (16)	−0.0051 (16)	0.0028 (19)
S17	0.016 (2)	0.015 (2)	0.019 (3)	−0.0034 (18)	0.0020 (17)	0.000 (2)
S18	0.011 (2)	0.015 (2)	0.023 (3)	0.0034 (17)	−0.0021 (17)	−0.003 (2)
S19	0.018 (2)	0.014 (2)	0.014 (2)	−0.0005 (18)	0.0021 (17)	0.001 (2)
S20	0.018 (2)	0.014 (2)	0.014 (2)	−0.0006 (18)	−0.0016 (17)	0.000 (2)
S21	0.016 (2)	0.015 (2)	0.027 (3)	−0.0002 (18)	−0.0015 (19)	−0.004 (2)
S22	0.047 (3)	0.015 (2)	0.013 (3)	−0.006 (2)	0.006 (2)	−0.004 (2)
S23	0.015 (2)	0.014 (2)	0.020 (3)	−0.0012 (17)	0.0019 (18)	−0.001 (2)
S24	0.013 (2)	0.016 (2)	0.019 (3)	−0.0003 (17)	−0.0010 (17)	0.007 (2)
S25	0.012 (2)	0.015 (2)	0.018 (3)	−0.0005 (17)	−0.0005 (17)	0.005 (2)
S26	0.021 (2)	0.021 (3)	0.017 (3)	−0.0040 (19)	0.0009 (18)	0.000 (2)
S27	0.015 (2)	0.012 (2)	0.014 (2)	−0.0024 (17)	0.0008 (17)	0.0075 (19)
S28	0.019 (2)	0.021 (3)	0.019 (3)	−0.0060 (19)	−0.0024 (18)	0.004 (2)
S29	0.016 (2)	0.017 (2)	0.012 (2)	−0.0006 (18)	0.0036 (17)	−0.0014 (19)
S30	0.018 (2)	0.014 (2)	0.020 (3)	0.0010 (18)	0.0029 (18)	−0.001 (2)
S31	0.014 (2)	0.015 (2)	0.017 (3)	−0.0023 (17)	0.0004 (17)	−0.0054 (19)
S32	0.017 (2)	0.016 (2)	0.015 (3)	0.0004 (18)	−0.0012 (17)	−0.006 (2)

### Geometric parameters (Å, º)

**Table:**

Cu1—S15^i^	2.285 (5)	Cu24—Cu34	2.644 (3)
Cu1—S14^i^	2.301 (5)	Cu24—Cu31^xi^	2.727 (3)
Cu1—S3	2.326 (5)	Cu25—S12^iv^	2.304 (4)
Cu1—Cu42^i^	2.697 (4)	Cu25—S2	2.335 (5)
Cu1—Cu22	2.734 (3)	Cu25—S6	2.338 (5)
Cu1—Cu44	2.745 (3)	Cu25—Cu30^iv^	2.612 (3)
Cu1—Cu33	2.858 (3)	Cu25—Cu26	2.788 (3)
Cu1—Cu51B^i^	2.872 (11)	Cu26—S11^iv^	2.290 (5)
Cu1—Cu23	2.884 (3)	Cu26—S6	2.301 (5)
Cu2—S15^i^	2.272 (5)	Cu26—S3	2.403 (5)
Cu2—S4	2.315 (5)	Cu26—Cu35	2.729 (3)
Cu2—S16^i^	2.336 (5)	Cu26—Cu29A^iv^	2.829 (5)
Cu2—Cu45A	2.705 (4)	Cu27—S10^iv^	2.265 (4)
Cu2—Cu21^ii^	2.720 (3)	Cu27—S7	2.411 (5)
Cu2—Cu45B	2.77 (3)	Cu27—S4	2.435 (5)
Cu2—Cu34	2.770 (3)	Cu27—Cu29A^iv^	2.539 (4)
Cu2—Cu23	2.779 (3)	Cu27—S8	2.586 (5)
Cu2—Cu24	2.847 (3)	Cu27—Cu36A	2.603 (4)
Cu2—Cu43^i^	2.870 (3)	Cu27—Cu36B	2.652 (17)
Cu3—S5	2.291 (5)	Cu27—Cu29B^iv^	2.682 (18)
Cu3—S2	2.346 (5)	Cu27—Cu28^iv^	2.790 (4)
Cu3—S1^iii^	2.357 (5)	Cu28—S8^iv^	2.228 (5)
Cu3—Cu62A	2.670 (4)	Cu28—S9^iii^	2.310 (5)
Cu3—Cu62B	2.706 (14)	Cu28—S10	2.694 (5)
Cu3—Cu32	2.729 (3)	Cu28—S5	2.727 (5)
Cu3—Cu31^iv^	2.735 (3)	Cu29A—S7^iv^	2.248 (5)
Cu3—Cu30^iv^	2.781 (3)	Cu29A—S6	2.343 (6)
Cu3—Cu21	2.816 (3)	Cu29A—S11	2.567 (7)
Cu3—Cu25	2.992 (3)	Cu29B—S6	2.059 (17)
Cu3—Cu37^iii^	3.058 (3)	Cu29B—S7^iv^	2.118 (16)
Cu4—S5^v^	2.262 (5)	Cu30—S5^iv^	2.267 (5)
Cu4—S8	2.278 (5)	Cu30—S12	2.302 (5)
Cu4—S9	2.305 (5)	Cu30—S9	2.370 (5)
Cu4—Cu28^v^	2.638 (3)	Cu30—Cu39	2.665 (3)
Cu4—Cu37	2.715 (3)	Cu30—Cu31	2.976 (3)
Cu4—Cu28^iv^	2.815 (3)	Cu31—S1^xi^	2.266 (4)
Cu4—Cu39	2.824 (3)	Cu31—S16	2.335 (4)
Cu4—Cu30	3.055 (3)	Cu31—S9	2.360 (5)
Cu4—Cu48^v^	3.060 (3)	Cu31—Cu40^v^	2.709 (3)
Cu5—S5	2.271 (5)	Cu32—S1^iii^	2.271 (5)
Cu5—S6	2.290 (5)	Cu32—S17	2.278 (4)
Cu5—S10	2.297 (5)	Cu32—S13^i^	2.388 (4)
Cu5—Cu29B	2.556 (16)	Cu32—Cu34^iii^	2.703 (3)
Cu5—Cu28	2.733 (3)	Cu32—Cu40^i^	2.742 (3)
Cu5—Cu29A	2.737 (5)	Cu32—Cu62B	2.968 (16)
Cu5—Cu46	2.748 (3)	Cu33—S18	2.263 (4)
Cu5—Cu49	2.856 (3)	Cu33—S2	2.335 (5)
Cu5—Cu25	2.928 (3)	Cu33—S14^i^	2.368 (5)
Cu5—Cu27^iv^	2.993 (3)	Cu33—Cu62B	2.491 (14)
Cu5—Cu47B	3.024 (14)	Cu33—Cu44	2.759 (3)
Cu6—S7	2.283 (5)	Cu33—Cu62A	2.845 (4)
Cu6—S11	2.339 (5)	Cu33—Cu41^i^	2.906 (3)
Cu6—S6	2.366 (5)	Cu34—S16^i^	2.295 (4)
Cu6—Cu29A	2.535 (4)	Cu34—S20	2.305 (4)
Cu6—Cu29B^iv^	2.539 (16)	Cu34—S1	2.334 (5)
Cu6—Cu29A^iv^	2.706 (4)	Cu34—Cu40^xii^	2.672 (3)
Cu6—Cu50	2.727 (3)	Cu34—Cu45A	2.985 (7)
Cu6—Cu29B	2.733 (18)	Cu35—S23	2.240 (5)
Cu6—Cu26	2.757 (3)	Cu35—S7	2.282 (5)
Cu6—Cu35	2.761 (3)	Cu35—S3	2.315 (5)
Cu6—Cu38	2.795 (3)	Cu35—Cu44	2.700 (3)
Cu6—Cu47A	2.875 (12)	Cu35—Cu47A	2.835 (12)
Cu7—S10	2.267 (5)	Cu36A—S24	2.236 (5)
Cu7—S9^iii^	2.307 (5)	Cu36A—S4	2.383 (8)
Cu7—S13	2.307 (4)	Cu36A—S8	2.384 (6)
Cu7—Cu40	2.612 (3)	Cu36A—Cu45A	2.562 (9)
Cu7—Cu28	2.690 (3)	Cu36A—S7	2.722 (7)
Cu7—Cu24^iv^	2.717 (3)	Cu36A—Cu37	2.989 (5)
Cu7—Cu31^iii^	2.793 (3)	Cu36B—S24	2.177 (17)
Cu7—Cu48	2.892 (3)	Cu36B—S8	2.21 (2)
Cu7—Cu41	2.907 (3)	Cu36B—S7	2.36 (2)
Cu8—S10	2.214 (5)	Cu36B—Cu39	2.93 (3)
Cu8—S11	2.273 (5)	Cu36B—Cu45B	2.99 (4)
Cu8—S14	2.342 (5)	Cu37—S8	2.267 (5)
Cu8—Cu42	2.566 (4)	Cu37—S21^v^	2.277 (5)
Cu8—Cu41	2.802 (3)	Cu37—S1	2.298 (5)
Cu8—Cu29A	2.815 (7)	Cu38—S27	2.285 (4)
Cu8—Cu23^iv^	2.838 (3)	Cu38—S7	2.285 (4)
Cu8—Cu27^iv^	2.892 (3)	Cu38—S12	2.296 (5)
Cu9—S11	2.297 (5)	Cu38—Cu50	2.765 (3)
Cu9—S15	2.299 (5)	Cu38—Cu39	2.771 (3)
Cu9—S12	2.327 (5)	Cu39—S8	2.268 (5)
Cu9—Cu22^iv^	2.715 (3)	Cu39—S28	2.274 (5)
Cu9—Cu26^iv^	2.774 (3)	Cu39—S12	2.328 (5)
Cu9—Cu42	2.778 (4)	Cu40—S13	2.295 (5)
Cu9—Cu25^iv^	2.781 (3)	Cu40—S29^iii^	2.317 (4)
Cu9—Cu43	2.850 (3)	Cu40—S16^iii^	2.352 (5)
Cu9—Cu51A	2.881 (7)	Cu41—S30	2.266 (5)
Cu9—Cu38	2.902 (3)	Cu41—S13	2.267 (5)
Cu10—S20^vi^	2.284 (5)	Cu41—S14	2.332 (5)
Cu10—S17^vii^	2.294 (5)	Cu42—S31	2.233 (5)
Cu10—S29	2.326 (5)	Cu42—S14	2.295 (5)
Cu10—Cu40^v^	2.687 (3)	Cu42—Cu51B	2.405 (13)
Cu10—Cu32^vii^	2.704 (3)	Cu42—S11	2.661 (5)
Cu10—Cu56^viii^	2.754 (3)	Cu42—Cu51A	2.759 (9)
Cu10—Cu34^vi^	2.760 (3)	Cu43—S32	2.246 (4)
Cu10—Cu61	2.895 (4)	Cu43—S12	2.274 (5)
Cu11—S17^vi^	2.233 (5)	Cu43—S16	2.281 (5)
Cu11—S30	2.273 (5)	Cu43—Cu51A	2.707 (9)
Cu11—S18^vi^	2.337 (5)	Cu44—S3	2.285 (4)
Cu11—Cu59	2.760 (3)	Cu44—S18	2.342 (5)
Cu11—Cu41	2.778 (3)	Cu44—S19	2.344 (5)
Cu11—Cu52^vi^	2.790 (3)	Cu44—Cu53	2.701 (3)
Cu11—Cu33^vi^	2.843 (3)	Cu44—Cu51B^i^	2.971 (11)
Cu11—Cu62B^vi^	2.936 (19)	Cu45A—S4	2.279 (6)
Cu11—Cu17^ix^	3.033 (3)	Cu45A—S19	2.312 (6)
Cu12—S32	2.185 (8)	Cu45A—S20	2.419 (10)
Cu12—Cu45A^vi^	2.210 (8)	Cu45B—S4	2.08 (2)
Cu12—S19^vi^	2.217 (7)	Cu45B—S19	2.17 (3)
Cu12—S20^vi^	2.229 (8)	Cu46—S5	2.236 (5)
Cu12—Cu61	2.235 (7)	Cu46—S22	2.279 (5)
Cu12—Cu51A	2.352 (10)	Cu46—S25	2.423 (5)
Cu12—Cu60B	2.591 (14)	Cu46—Cu54A	2.592 (4)
Cu12—Cu45B^vi^	2.64 (4)	Cu46—Cu49	2.850 (4)
Cu12—Cu54B^viii^	2.653 (15)	Cu46—Cu62A	2.901 (5)
Cu12—Cu51B	2.709 (14)	Cu46—Cu54B	2.910 (13)
Cu12—Cu60A	2.742 (8)	Cu46—Cu47B	2.926 (14)
Cu13—S17	2.231 (5)	Cu47A—S6	2.228 (11)
Cu13—S21	2.245 (5)	Cu47A—S26	2.360 (13)
Cu13—Cu62B	2.363 (13)	Cu47A—S23	2.399 (13)
Cu13—S22	2.470 (5)	Cu47A—Cu55	2.564 (11)
Cu13—Cu54A	2.714 (5)	Cu47A—S22	2.792 (13)
Cu13—Cu62A	2.722 (4)	Cu47A—Cu50	3.046 (13)
Cu13—Cu54B	2.728 (15)	Cu47B—S6	2.215 (15)
Cu13—Cu52	2.829 (4)	Cu47B—S26	2.348 (17)
Cu13—Cu46	2.958 (4)	Cu47B—S22	2.373 (16)
Cu14—S24	2.264 (5)	Cu47B—Cu55	2.621 (14)
Cu14—S19	2.319 (5)	Cu47B—Cu49	2.943 (16)
Cu14—S23	2.337 (5)	Cu48—S9^iii^	2.257 (4)
Cu14—Cu45B	2.54 (3)	Cu48—S25	2.447 (5)
Cu14—Cu54B^x^	2.682 (15)	Cu48—S29^iii^	2.531 (5)
Cu14—Cu53	2.760 (3)	Cu48—S28^iii^	2.626 (5)
Cu14—Cu35	2.806 (3)	Cu49—S26	2.240 (5)
Cu14—Cu56	2.811 (3)	Cu49—S10	2.290 (5)
Cu14—Cu45A	2.852 (6)	Cu49—S25	2.344 (5)
Cu14—Cu44	2.876 (3)	Cu50—S11	2.280 (5)
Cu14—Cu54A^x^	2.973 (5)	Cu50—S26	2.301 (5)
Cu15—S24	2.282 (5)	Cu50—S27	2.310 (5)
Cu15—S20	2.301 (5)	Cu50—Cu57	2.658 (3)
Cu15—S21^v^	2.310 (5)	Cu51A—S15	2.208 (7)
Cu15—Cu53^x^	2.702 (3)	Cu51A—S32	2.494 (9)
Cu15—Cu37	2.740 (3)	Cu51A—S19^vi^	2.539 (9)
Cu15—Cu55^x^	2.788 (4)	Cu51A—S31	2.593 (9)
Cu15—Cu52^x^	2.790 (3)	Cu51A—Cu60A	2.616 (8)
Cu15—Cu36A	2.794 (5)	Cu51B—S19^vi^	2.249 (12)
Cu15—Cu45A	2.846 (6)	Cu51B—S15	2.282 (10)
Cu16—S21^v^	2.225 (5)	Cu51B—S31	2.325 (12)
Cu16—S28	2.258 (5)	Cu51B—Cu60B	2.531 (14)
Cu16—S25^v^	2.327 (5)	Cu52—S18	2.290 (5)
Cu16—Cu48^v^	2.622 (4)	Cu52—S24^xiii^	2.292 (5)
Cu16—Cu54A^v^	2.752 (5)	Cu52—S22	2.303 (5)
Cu16—Cu59^viii^	2.920 (3)	Cu52—Cu62B	2.489 (13)
Cu16—Cu46^v^	2.973 (4)	Cu52—Cu53	2.748 (3)
Cu17—S24	2.243 (5)	Cu52—Cu62A	2.761 (4)
Cu17—S28	2.277 (5)	Cu53—S21^x^	2.267 (5)
Cu17—S27	2.297 (5)	Cu53—S23	2.286 (5)
Cu17—Cu58	2.768 (3)	Cu53—S18	2.344 (5)
Cu17—Cu39	2.785 (3)	Cu53—Cu55	2.899 (4)
Cu17—Cu56	2.818 (3)	Cu54A—S19^xiii^	2.224 (5)
Cu17—Cu36B	2.82 (2)	Cu54A—S22	2.467 (7)
Cu17—Cu38	2.863 (3)	Cu54A—S25	2.604 (6)
Cu18—S30	2.232 (5)	Cu54A—S21	2.771 (6)
Cu18—S29^iii^	2.303 (5)	Cu54A—Cu60A^ix^	2.833 (8)
Cu18—S25	2.384 (5)	Cu54B—S22	1.942 (14)
Cu18—Cu48	2.624 (3)	Cu54B—S19^xiii^	2.206 (13)
Cu18—Cu57^ix^	2.798 (3)	Cu54B—Cu60B^ix^	2.72 (2)
Cu18—Cu58^ix^	2.799 (3)	Cu55—S20^xiii^	2.241 (5)
Cu18—Cu60A^ix^	2.840 (6)	Cu55—S23	2.276 (5)
Cu18—Cu49	2.879 (3)	Cu55—S22	2.452 (6)
Cu19—S30	2.270 (5)	Cu55—Cu61^ix^	2.645 (4)
Cu19—S26	2.291 (5)	Cu55—S26	2.764 (5)
Cu19—S31	2.325 (5)	Cu56—S23	2.260 (5)
Cu19—Cu50	2.779 (3)	Cu56—S17^x^	2.268 (4)
Cu19—Cu57	2.798 (3)	Cu56—S27	2.282 (5)
Cu19—Cu42	2.805 (4)	Cu57—S29^ix^	2.274 (4)
Cu19—Cu59	2.903 (3)	Cu57—S31	2.292 (5)
Cu19—Cu49	2.933 (3)	Cu57—S27	2.327 (5)
Cu20—S28	2.250 (5)	Cu57—Cu58	2.754 (3)
Cu20—S29	2.258 (5)	Cu57—Cu60A	2.892 (7)
Cu20—S32	2.288 (5)	Cu58—S27	2.282 (5)
Cu20—Cu48^v^	2.722 (3)	Cu58—S30^viii^	2.289 (5)
Cu20—Cu61	2.735 (4)	Cu58—S32	2.297 (5)
Cu20—Cu58	2.818 (3)	Cu58—Cu60A	2.906 (8)
Cu20—Cu43	2.896 (3)	Cu59—S28^ix^	2.258 (5)
Cu21—S16^iv^	2.275 (4)	Cu59—S31	2.266 (5)
Cu21—S13^i^	2.327 (4)	Cu59—S18^vi^	2.277 (5)
Cu21—S2	2.341 (5)	Cu60A—S25^viii^	2.240 (6)
Cu21—Cu31^iv^	2.631 (3)	Cu60A—S32	2.272 (7)
Cu21—Cu32	2.724 (3)	Cu60A—S31	2.380 (6)
Cu21—Cu24^ii^	2.746 (3)	Cu60B—S25^viii^	2.222 (11)
Cu22—S3	2.264 (5)	Cu60B—S31	2.396 (13)
Cu22—S15^iv^	2.269 (5)	Cu60B—S19^vi^	2.452 (15)
Cu22—S2	2.346 (4)	Cu60B—S32	2.724 (14)
Cu22—Cu33	2.699 (3)	Cu61—S26^viii^	2.230 (5)
Cu22—Cu26	2.714 (3)	Cu61—S32	2.268 (5)
Cu22—Cu25	2.742 (3)	Cu61—S20^vi^	2.411 (5)
Cu23—S14^iv^	2.265 (4)	Cu62A—S22	2.141 (6)
Cu23—S4	2.267 (5)	Cu62A—S2	2.204 (5)
Cu23—S3	2.284 (5)	Cu62B—S2	2.199 (13)
Cu24—S13^iv^	2.272 (4)	Cu62B—S22	2.592 (16)
Cu24—S4	2.316 (5)	Cu62B—S17	2.607 (19)
Cu24—S1	2.364 (5)		
			
S15^i^—Cu1—S14^i^	118.62 (18)	S16^iii^—Cu40—Cu34^xv^	53.90 (11)
S15^i^—Cu1—S3	113.59 (18)	Cu7—Cu40—Cu34^xv^	154.35 (11)
S14^i^—Cu1—S3	127.51 (18)	S13—Cu40—Cu10^iii^	107.31 (13)
S15^i^—Cu1—Cu42^i^	72.74 (14)	S29^iii^—Cu40—Cu10^iii^	54.80 (12)
S14^i^—Cu1—Cu42^i^	53.96 (13)	S16^iii^—Cu40—Cu10^iii^	106.23 (13)
S3—Cu1—Cu42^i^	146.74 (14)	Cu7—Cu40—Cu10^iii^	136.81 (11)
S15^i^—Cu1—Cu22	151.82 (15)	Cu34^xv^—Cu40—Cu10^iii^	61.99 (8)
S14^i^—Cu1—Cu22	81.69 (13)	S13—Cu40—Cu31^iii^	82.46 (12)
S3—Cu1—Cu22	52.39 (12)	S29^iii^—Cu40—Cu31^iii^	134.11 (15)
Cu42^i^—Cu1—Cu22	133.56 (12)	S16^iii^—Cu40—Cu31^iii^	54.39 (11)
S15^i^—Cu1—Cu44	105.19 (14)	Cu7—Cu40—Cu31^iii^	63.29 (8)
S14^i^—Cu1—Cu44	106.48 (13)	Cu34^xv^—Cu40—Cu31^iii^	98.60 (9)
S3—Cu1—Cu44	52.77 (11)	Cu10^iii^—Cu40—Cu31^iii^	159.83 (11)
Cu42^i^—Cu1—Cu44	94.00 (10)	S13—Cu40—Cu32^vi^	55.75 (12)
Cu22—Cu1—Cu44	85.29 (9)	S29^iii^—Cu40—Cu32^vi^	108.73 (14)
S15^i^—Cu1—Cu33	149.78 (15)	S16^iii^—Cu40—Cu32^vi^	107.80 (14)
S14^i^—Cu1—Cu33	53.31 (12)	Cu7—Cu40—Cu32^vi^	110.34 (10)
S3—Cu1—Cu33	78.71 (13)	Cu34^xv^—Cu40—Cu32^vi^	59.88 (8)
Cu42^i^—Cu1—Cu33	82.41 (10)	Cu10^iii^—Cu40—Cu32^vi^	59.73 (8)
Cu22—Cu1—Cu33	57.65 (8)	Cu31^iii^—Cu40—Cu32^vi^	117.08 (10)
Cu44—Cu1—Cu33	58.95 (8)	S30—Cu41—S13	128.41 (18)
S15^i^—Cu1—Cu51B^i^	51.0 (2)	S30—Cu41—S14	119.03 (18)
S14^i^—Cu1—Cu51B^i^	102.5 (3)	S13—Cu41—S14	111.51 (17)
S3—Cu1—Cu51B^i^	105.8 (3)	S30—Cu41—Cu11	52.38 (13)
Cu42^i^—Cu1—Cu51B^i^	51.0 (3)	S13—Cu41—Cu11	105.11 (14)
Cu22—Cu1—Cu51B^i^	148.9 (2)	S14—Cu41—Cu11	105.95 (14)
Cu44—Cu1—Cu51B^i^	63.8 (2)	S30—Cu41—Cu8	99.18 (15)
Cu33—Cu1—Cu51B^i^	99.8 (2)	S13—Cu41—Cu8	119.45 (14)
S15^i^—Cu1—Cu23	68.99 (13)	S14—Cu41—Cu8	53.34 (12)
S14^i^—Cu1—Cu23	161.14 (14)	Cu11—Cu41—Cu8	134.99 (11)
S3—Cu1—Cu23	50.63 (12)	S30—Cu41—Cu33^vi^	102.98 (14)
Cu42^i^—Cu1—Cu23	140.42 (12)	S13—Cu41—Cu33^vi^	99.36 (14)
Cu22—Cu1—Cu23	85.98 (9)	S14—Cu41—Cu33^vi^	52.35 (12)
Cu44—Cu1—Cu23	86.57 (9)	Cu11—Cu41—Cu33^vi^	59.97 (8)
Cu33—Cu1—Cu23	129.33 (11)	Cu8—Cu41—Cu33^vi^	104.11 (10)
Cu51B^i^—Cu1—Cu23	95.4 (3)	S30—Cu41—Cu7	104.11 (15)
S15^i^—Cu2—S4	118.77 (18)	S13—Cu41—Cu7	51.16 (12)
S15^i^—Cu2—S16^i^	116.36 (18)	S14—Cu41—Cu7	123.07 (13)
S4—Cu2—S16^i^	124.78 (18)	Cu11—Cu41—Cu7	130.17 (10)
S15^i^—Cu2—Cu45A	106.66 (18)	Cu8—Cu41—Cu7	86.37 (9)
S4—Cu2—Cu45A	53.31 (16)	Cu33^vi^—Cu41—Cu7	148.89 (11)
S16^i^—Cu2—Cu45A	112.4 (2)	S31—Cu42—S14	135.4 (2)
S15^i^—Cu2—Cu21^ii^	106.64 (14)	S31—Cu42—Cu51B	60.1 (3)
S4—Cu2—Cu21^ii^	106.83 (14)	S14—Cu42—Cu51B	119.0 (3)
S16^i^—Cu2—Cu21^ii^	52.81 (11)	S31—Cu42—Cu8	130.62 (18)
Cu45A—Cu2—Cu21^ii^	146.60 (16)	S14—Cu42—Cu8	57.29 (13)
S15^i^—Cu2—Cu45B	97.9 (7)	Cu51B—Cu42—Cu8	168.9 (3)
S4—Cu2—Cu45B	47.3 (6)	S31—Cu42—S11	115.95 (18)
S16^i^—Cu2—Cu45B	126.8 (9)	S14—Cu42—S11	100.75 (16)
Cu21^ii^—Cu2—Cu45B	151.7 (6)	Cu51B—Cu42—S11	123.8 (3)
S15^i^—Cu2—Cu34	154.81 (15)	Cu8—Cu42—S11	51.52 (12)
S4—Cu2—Cu34	77.46 (13)	S31—Cu42—Cu1^vi^	119.78 (17)
S16^i^—Cu2—Cu34	52.59 (12)	S14—Cu42—Cu1^vi^	54.16 (13)
Cu45A—Cu2—Cu34	66.07 (18)	Cu8—Cu42—Cu1^vi^	104.08 (11)
Cu21^ii^—Cu2—Cu34	84.50 (9)	S11—Cu42—Cu1^vi^	117.42 (13)
Cu45B—Cu2—Cu34	78.9 (9)	S31—Cu42—Cu51A	61.52 (18)
S15^i^—Cu2—Cu23	71.21 (14)	S14—Cu42—Cu51A	129.1 (2)
S4—Cu2—Cu23	51.88 (12)	Cu8—Cu42—Cu51A	159.7 (2)
S16^i^—Cu2—Cu23	156.06 (14)	S11—Cu42—Cu51A	110.0 (2)
Cu45A—Cu2—Cu23	84.7 (2)	Cu1^vi^—Cu42—Cu51A	75.67 (17)
Cu21^ii^—Cu2—Cu23	103.66 (9)	S31—Cu42—Cu9	103.80 (16)
Cu45B—Cu2—Cu23	70.9 (9)	S14—Cu42—Cu9	119.50 (14)
Cu34—Cu2—Cu23	129.09 (11)	Cu51B—Cu42—Cu9	75.4 (3)
S15^i^—Cu2—Cu24	148.85 (15)	Cu8—Cu42—Cu9	97.08 (11)
S4—Cu2—Cu24	52.09 (12)	S11—Cu42—Cu9	49.90 (12)
S16^i^—Cu2—Cu24	78.67 (13)	Cu1^vi^—Cu42—Cu9	90.34 (11)
Cu45A—Cu2—Cu24	90.27 (11)	Cu51A—Cu42—Cu9	62.69 (18)
Cu21^ii^—Cu2—Cu24	59.05 (8)	S31—Cu42—Cu19	53.52 (13)
Cu45B—Cu2—Cu24	92.6 (6)	S14—Cu42—Cu19	105.79 (15)
Cu34—Cu2—Cu24	56.15 (7)	Cu51B—Cu42—Cu19	113.5 (3)
Cu23—Cu2—Cu24	84.94 (9)	Cu8—Cu42—Cu19	77.38 (11)
S15^i^—Cu2—Cu43^i^	72.77 (14)	S11—Cu42—Cu19	89.11 (13)
S4—Cu2—Cu43^i^	155.55 (14)	Cu1^vi^—Cu42—Cu19	147.79 (14)
S16^i^—Cu2—Cu43^i^	50.72 (12)	Cu51A—Cu42—Cu19	113.96 (16)
Cu45A—Cu2—Cu43^i^	103.81 (16)	Cu9—Cu42—Cu19	121.68 (12)
Cu21^ii^—Cu2—Cu43^i^	88.39 (9)	S32—Cu43—S12	121.51 (18)
Cu34—Cu2—Cu43^i^	85.31 (9)	S32—Cu43—S16	125.13 (17)
Cu23—Cu2—Cu43^i^	143.93 (11)	S12—Cu43—S16	109.47 (16)
Cu24—Cu2—Cu43^i^	129.20 (10)	S32—Cu43—Cu51A	59.60 (19)
S5—Cu3—S2	122.84 (18)	S12—Cu43—Cu51A	112.9 (2)
S5—Cu3—S1^iii^	111.37 (18)	S16—Cu43—Cu51A	118.2 (2)
S2—Cu3—S1^iii^	125.14 (17)	S32—Cu43—Cu9	104.52 (14)
S5—Cu3—Cu62A	94.27 (16)	S12—Cu43—Cu9	52.56 (12)
S2—Cu3—Cu62A	51.63 (12)	S16—Cu43—Cu9	122.71 (14)
S1^iii^—Cu3—Cu62A	135.32 (16)	Cu51A—Cu43—Cu9	62.38 (17)
S5—Cu3—Cu62B	112.4 (4)	S32—Cu43—Cu2^vi^	108.65 (15)
S2—Cu3—Cu62B	51.0 (3)	S12—Cu43—Cu2^vi^	121.13 (14)
S1^iii^—Cu3—Cu62B	115.7 (4)	S16—Cu43—Cu2^vi^	52.44 (12)
S5—Cu3—Cu32	149.95 (15)	Cu51A—Cu43—Cu2^vi^	67.46 (17)
S2—Cu3—Cu32	80.96 (13)	Cu9—Cu43—Cu2^vi^	88.32 (9)
S1^iii^—Cu3—Cu32	52.43 (12)	S32—Cu43—Cu20	50.95 (13)
Cu62A—Cu3—Cu32	87.19 (12)	S12—Cu43—Cu20	102.26 (14)
Cu62B—Cu3—Cu32	66.2 (4)	S16—Cu43—Cu20	101.80 (14)
S5—Cu3—Cu31^iv^	103.45 (14)	Cu51A—Cu43—Cu20	110.55 (16)
S2—Cu3—Cu31^iv^	104.08 (13)	Cu9—Cu43—Cu20	133.25 (10)
S1^iii^—Cu3—Cu31^iv^	52.19 (11)	Cu2^vi^—Cu43—Cu20	134.15 (10)
Cu62A—Cu3—Cu31^iv^	155.56 (12)	S3—Cu44—S18	131.19 (18)
Cu32—Cu3—Cu31^iv^	85.77 (9)	S3—Cu44—S19	119.41 (17)
S5—Cu3—Cu30^iv^	52.01 (12)	S18—Cu44—S19	107.88 (17)
S2—Cu3—Cu30^iv^	98.65 (13)	S3—Cu44—Cu35	54.59 (12)
S1^iii^—Cu3—Cu30^iv^	108.50 (13)	S18—Cu44—Cu35	124.32 (15)
Cu62A—Cu3—Cu30^iv^	116.09 (13)	S19—Cu44—Cu35	105.48 (14)
Cu32—Cu3—Cu30^iv^	150.23 (11)	S3—Cu44—Cu53	140.57 (16)
Cu31^iv^—Cu3—Cu30^iv^	65.30 (8)	S18—Cu44—Cu53	54.83 (12)
S5—Cu3—Cu21	149.03 (14)	S19—Cu44—Cu53	81.10 (13)
S2—Cu3—Cu21	52.99 (12)	Cu35—Cu44—Cu53	88.85 (10)
S1^iii^—Cu3—Cu21	76.59 (13)	S3—Cu44—Cu1	54.16 (12)
Cu62A—Cu3—Cu21	100.06 (11)	S18—Cu44—Cu1	105.34 (14)
Cu62B—Cu3—Cu21	88.3 (3)	S19—Cu44—Cu1	103.49 (14)
Cu32—Cu3—Cu21	58.81 (7)	Cu35—Cu44—Cu1	108.54 (10)
Cu31^iv^—Cu3—Cu21	56.55 (7)	Cu53—Cu44—Cu1	159.63 (12)
Cu30^iv^—Cu3—Cu21	97.04 (9)	S3—Cu44—Cu33	81.54 (13)
S5—Cu3—Cu25	76.43 (13)	S18—Cu44—Cu33	51.88 (12)
S2—Cu3—Cu25	50.10 (12)	S19—Cu44—Cu33	143.21 (15)
S1^iii^—Cu3—Cu25	151.73 (13)	Cu35—Cu44—Cu33	111.21 (11)
Cu62A—Cu3—Cu25	67.77 (10)	Cu53—Cu44—Cu33	101.79 (10)
Cu62B—Cu3—Cu25	83.3 (4)	Cu1—Cu44—Cu33	62.58 (8)
Cu32—Cu3—Cu25	130.80 (10)	S3—Cu44—Cu14	105.33 (14)
Cu31^iv^—Cu3—Cu25	99.91 (9)	S18—Cu44—Cu14	113.33 (13)
Cu30^iv^—Cu3—Cu25	53.64 (7)	S19—Cu44—Cu14	51.51 (12)
Cu21—Cu3—Cu25	83.79 (8)	Cu35—Cu44—Cu14	60.34 (8)
S5—Cu3—Cu37^iii^	69.95 (13)	Cu53—Cu44—Cu14	59.22 (8)
S2—Cu3—Cu37^iii^	160.70 (14)	Cu1—Cu44—Cu14	138.67 (11)
S1^iii^—Cu3—Cu37^iii^	48.10 (12)	Cu33—Cu44—Cu14	157.56 (11)
Cu62A—Cu3—Cu37^iii^	117.38 (10)	S3—Cu44—Cu51B^i^	103.9 (2)
Cu32—Cu3—Cu37^iii^	82.73 (9)	S18—Cu44—Cu51B^i^	98.3 (3)
Cu31^iv^—Cu3—Cu37^iii^	84.87 (8)	S19—Cu44—Cu51B^i^	48.3 (2)
Cu30^iv^—Cu3—Cu37^iii^	100.63 (9)	Cu35—Cu44—Cu51B^i^	136.8 (3)
Cu21—Cu3—Cu37^iii^	124.68 (10)	Cu53—Cu44—Cu51B^i^	114.0 (2)
Cu25—Cu3—Cu37^iii^	146.20 (10)	Cu1—Cu44—Cu51B^i^	60.2 (2)
S5^v^—Cu4—S8	124.53 (19)	Cu33—Cu44—Cu51B^i^	99.8 (3)
S5^v^—Cu4—S9	115.93 (18)	Cu14—Cu44—Cu51B^i^	99.2 (2)
S8—Cu4—S9	119.18 (18)	Cu12^i^—Cu45A—S4	135.6 (3)
S5^v^—Cu4—Cu28^v^	67.13 (14)	Cu12^i^—Cu45A—S19	58.7 (2)
S8—Cu4—Cu28^v^	148.38 (16)	S4—Cu45A—S19	128.1 (4)
S9—Cu4—Cu28^v^	55.22 (13)	Cu12^i^—Cu45A—S20	57.3 (3)
S5^v^—Cu4—Cu37	77.34 (14)	S4—Cu45A—S20	120.5 (3)
S8—Cu4—Cu37	53.14 (13)	S19—Cu45A—S20	107.4 (3)
S9—Cu4—Cu37	157.98 (15)	Cu12^i^—Cu45A—Cu36A	163.2 (3)
Cu28^v^—Cu4—Cu37	143.61 (12)	S4—Cu45A—Cu36A	58.63 (19)
S5^v^—Cu4—Cu28^iv^	104.98 (14)	S19—Cu45A—Cu36A	123.2 (3)
S8—Cu4—Cu28^iv^	50.55 (12)	S20—Cu45A—Cu36A	109.7 (2)
S9—Cu4—Cu28^iv^	109.42 (14)	Cu12^i^—Cu45A—Cu2	81.2 (2)
Cu28^v^—Cu4—Cu28^iv^	99.50 (10)	S4—Cu45A—Cu2	54.54 (14)
Cu37—Cu4—Cu28^iv^	81.84 (10)	S19—Cu45A—Cu2	102.7 (2)
S5^v^—Cu4—Cu39	156.55 (16)	S20—Cu45A—Cu2	97.9 (3)
S8—Cu4—Cu39	51.45 (13)	Cu36A—Cu45A—Cu2	112.83 (19)
S9—Cu4—Cu39	75.67 (13)	Cu12^i^—Cu45A—Cu15	102.0 (3)
Cu28^v^—Cu4—Cu39	130.23 (12)	S4—Cu45A—Cu15	105.71 (18)
Cu37—Cu4—Cu39	86.04 (10)	S19—Cu45A—Cu15	119.97 (19)
Cu28^iv^—Cu4—Cu39	88.65 (9)	S20—Cu45A—Cu15	51.04 (17)
S5^v^—Cu4—Cu30	149.50 (15)	Cu36A—Cu45A—Cu15	61.95 (13)
S8—Cu4—Cu30	73.16 (14)	Cu2—Cu45A—Cu15	132.0 (2)
S9—Cu4—Cu30	50.12 (12)	Cu12^i^—Cu45A—Cu14	102.5 (2)
Cu28^v^—Cu4—Cu30	85.50 (10)	S4—Cu45A—Cu14	113.2 (3)
Cu37—Cu4—Cu30	126.15 (11)	S19—Cu45A—Cu14	52.09 (16)
Cu28^iv^—Cu4—Cu30	65.16 (9)	S20—Cu45A—Cu14	117.3 (2)
Cu39—Cu4—Cu30	53.73 (7)	Cu36A—Cu45A—Cu14	72.9 (2)
S5^v^—Cu4—Cu48^v^	107.41 (14)	Cu2—Cu45A—Cu14	140.6 (3)
S8—Cu4—Cu48^v^	113.77 (14)	Cu15—Cu45A—Cu14	86.13 (12)
S9—Cu4—Cu48^v^	47.22 (11)	Cu12^i^—Cu45A—Cu34	81.1 (3)
Cu28^v^—Cu4—Cu48^v^	85.30 (9)	S4—Cu45A—Cu34	73.52 (16)
Cu37—Cu4—Cu48^v^	113.56 (10)	S19—Cu45A—Cu34	138.6 (3)
Cu28^iv^—Cu4—Cu48^v^	146.45 (11)	S20—Cu45A—Cu34	49.13 (17)
Cu39—Cu4—Cu48^v^	64.26 (8)	Cu36A—Cu45A—Cu34	98.13 (18)
Cu30—Cu4—Cu48^v^	82.32 (9)	Cu2—Cu45A—Cu34	58.00 (12)
S5—Cu5—S6	121.35 (18)	Cu15—Cu45A—Cu34	75.02 (19)
S5—Cu5—S10	116.61 (18)	Cu14—Cu45A—Cu34	161.1 (2)
S6—Cu5—S10	120.26 (18)	S4—Cu45B—S19	153.1 (16)
S5—Cu5—Cu29B	143.1 (6)	S4—Cu45B—Cu14	136.7 (19)
S6—Cu5—Cu29B	49.9 (4)	S19—Cu45B—Cu14	58.4 (7)
S10—Cu5—Cu29B	89.9 (6)	S4—Cu45B—Cu12^i^	123.0 (17)
S5—Cu5—Cu28	65.30 (13)	S19—Cu45B—Cu12^i^	53.9 (8)
S6—Cu5—Cu28	157.37 (14)	Cu14—Cu45B—Cu12^i^	100.0 (9)
S10—Cu5—Cu28	64.07 (13)	S4—Cu45B—Cu2	54.9 (6)
Cu29B—Cu5—Cu28	110.6 (4)	S19—Cu45B—Cu2	104.8 (12)
S5—Cu5—Cu29A	157.31 (19)	Cu14—Cu45B—Cu2	160.9 (13)
S6—Cu5—Cu29A	54.69 (14)	Cu12^i^—Cu45B—Cu2	73.0 (10)
S10—Cu5—Cu29A	76.82 (17)	S4—Cu45B—Cu36B	69.2 (9)
Cu28—Cu5—Cu29A	109.95 (12)	S19—Cu45B—Cu36B	132.1 (14)
S5—Cu5—Cu46	51.85 (12)	Cu14—Cu45B—Cu36B	73.8 (9)
S6—Cu5—Cu46	100.82 (14)	Cu12^i^—Cu45B—Cu36B	141.9 (15)
S10—Cu5—Cu46	103.37 (14)	Cu2—Cu45B—Cu36B	122.7 (9)
Cu29B—Cu5—Cu46	150.1 (4)	S5—Cu46—S22	136.4 (2)
Cu28—Cu5—Cu46	99.34 (10)	S5—Cu46—S25	113.66 (18)
Cu29A—Cu5—Cu46	146.70 (13)	S22—Cu46—S25	109.83 (18)
S5—Cu5—Cu49	104.86 (13)	S5—Cu46—Cu54A	147.8 (2)
S6—Cu5—Cu49	99.29 (13)	S22—Cu46—Cu54A	60.44 (18)
S10—Cu5—Cu49	51.38 (12)	S25—Cu46—Cu54A	62.47 (15)
Cu29B—Cu5—Cu49	111.8 (6)	S5—Cu46—Cu5	53.00 (13)
Cu28—Cu5—Cu49	99.38 (10)	S22—Cu46—Cu5	114.57 (17)
Cu29A—Cu5—Cu49	97.79 (15)	S25—Cu46—Cu5	102.25 (15)
Cu46—Cu5—Cu49	61.10 (9)	Cu54A—Cu46—Cu5	156.44 (16)
S5—Cu5—Cu25	78.08 (13)	S5—Cu46—Cu49	106.02 (16)
S6—Cu5—Cu25	51.49 (12)	S22—Cu46—Cu49	97.91 (16)
S10—Cu5—Cu25	160.32 (15)	S25—Cu46—Cu49	52.01 (13)
Cu29B—Cu5—Cu25	71.4 (6)	Cu54A—Cu46—Cu49	95.69 (14)
Cu28—Cu5—Cu25	116.11 (10)	Cu5—Cu46—Cu49	61.31 (9)
Cu29A—Cu5—Cu25	85.24 (15)	S5—Cu46—Cu62A	89.44 (15)
Cu46—Cu5—Cu25	96.06 (10)	S22—Cu46—Cu62A	46.96 (15)
Cu49—Cu5—Cu25	141.06 (11)	S25—Cu46—Cu62A	156.65 (15)
S5—Cu5—Cu27^iv^	121.34 (15)	Cu54A—Cu46—Cu62A	99.96 (15)
S6—Cu5—Cu27^iv^	106.43 (14)	Cu5—Cu46—Cu62A	88.75 (12)
S10—Cu5—Cu27^iv^	48.55 (12)	Cu49—Cu46—Cu62A	120.35 (14)
Cu28—Cu5—Cu27^iv^	58.11 (9)	S5—Cu46—Cu54B	159.8 (4)
Cu29A—Cu5—Cu27^iv^	52.39 (10)	S22—Cu46—Cu54B	41.8 (4)
Cu46—Cu5—Cu27^iv^	148.36 (12)	S25—Cu46—Cu54B	74.6 (3)
Cu49—Cu5—Cu27^iv^	98.34 (10)	Cu5—Cu46—Cu54B	145.7 (4)
Cu25—Cu5—Cu27^iv^	113.37 (9)	Cu49—Cu46—Cu54B	93.5 (3)
S5—Cu5—Cu47B	105.6 (3)	S5—Cu46—Cu47B	109.8 (3)
S6—Cu5—Cu47B	46.8 (3)	S22—Cu46—Cu47B	52.5 (3)
S10—Cu5—Cu47B	105.1 (4)	S25—Cu46—Cu47B	106.9 (4)
Cu29B—Cu5—Cu47B	90.1 (5)	Cu5—Cu46—Cu47B	64.3 (3)
Cu28—Cu5—Cu47B	155.8 (3)	Cu49—Cu46—Cu47B	61.3 (4)
Cu46—Cu5—Cu47B	60.7 (3)	Cu54B—Cu46—Cu47B	83.6 (5)
Cu49—Cu5—Cu47B	60.0 (3)	S5—Cu46—Cu13	105.74 (16)
Cu25—Cu5—Cu47B	81.6 (3)	S22—Cu46—Cu13	54.43 (14)
Cu27^iv^—Cu5—Cu47B	132.4 (3)	S25—Cu46—Cu13	117.13 (15)
S7—Cu6—S11	125.84 (18)	Cu54A—Cu46—Cu13	58.11 (12)
S7—Cu6—S6	125.68 (18)	Cu5—Cu46—Cu13	140.60 (13)
S11—Cu6—S6	108.42 (17)	Cu49—Cu46—Cu13	147.87 (11)
S7—Cu6—Cu29A	150.24 (17)	Cu62A—Cu46—Cu13	55.35 (11)
S11—Cu6—Cu29A	63.41 (19)	Cu54B—Cu46—Cu13	55.4 (3)
S6—Cu6—Cu29A	56.99 (16)	Cu47B—Cu46—Cu13	103.0 (4)
S7—Cu6—Cu29B^iv^	51.8 (4)	S5—Cu46—Cu16^iii^	93.36 (15)
S11—Cu6—Cu29B^iv^	100.4 (5)	S22—Cu46—Cu16^iii^	117.57 (16)
S6—Cu6—Cu29B^iv^	125.3 (6)	S25—Cu46—Cu16^iii^	49.82 (12)
Cu29A—Cu6—Cu29B^iv^	100.6 (4)	Cu54A—Cu46—Cu16^iii^	58.78 (13)
S7—Cu6—Cu29A^iv^	52.73 (13)	Cu5—Cu46—Cu16^iii^	126.70 (12)
S11—Cu6—Cu29A^iv^	112.43 (18)	Cu49—Cu46—Cu16^iii^	100.73 (11)
S6—Cu6—Cu29A^iv^	111.25 (18)	Cu62A—Cu46—Cu16^iii^	136.23 (14)
Cu29A—Cu6—Cu29A^iv^	97.65 (12)	Cu13—Cu46—Cu16^iii^	82.06 (10)
S7—Cu6—Cu50	104.96 (13)	S6—Cu47A—S26	121.5 (6)
S11—Cu6—Cu50	52.84 (12)	S6—Cu47A—S23	128.3 (5)
S6—Cu6—Cu50	105.47 (13)	S26—Cu47A—S23	102.1 (4)
Cu29A—Cu6—Cu50	101.80 (16)	S6—Cu47A—Cu55	164.0 (6)
Cu29B^iv^—Cu6—Cu50	128.9 (6)	S26—Cu47A—Cu55	68.1 (3)
Cu29A^iv^—Cu6—Cu50	143.27 (17)	S23—Cu47A—Cu55	54.5 (3)
S7—Cu6—Cu29B	141.3 (5)	S6—Cu47A—S22	110.8 (5)
S11—Cu6—Cu29B	78.2 (5)	S26—Cu47A—S22	90.4 (4)
S6—Cu6—Cu29B	46.9 (4)	S23—Cu47A—S22	94.0 (4)
Cu29B^iv^—Cu6—Cu29B	98.3 (5)	Cu55—Cu47A—S22	54.3 (3)
Cu50—Cu6—Cu29B	113.6 (5)	S6—Cu47A—Cu35	78.8 (3)
S7—Cu6—Cu26	80.21 (13)	S26—Cu47A—Cu35	134.8 (5)
S11—Cu6—Cu26	145.43 (14)	S23—Cu47A—Cu35	49.8 (3)
S6—Cu6—Cu26	52.70 (12)	Cu55—Cu47A—Cu35	103.6 (4)
Cu29A—Cu6—Cu26	82.89 (17)	S22—Cu47A—Cu35	121.7 (5)
Cu29B^iv^—Cu6—Cu26	77.4 (6)	S6—Cu47A—Cu6	53.4 (3)
Cu29A^iv^—Cu6—Cu26	62.35 (15)	S26—Cu47A—Cu6	99.8 (5)
Cu50—Cu6—Cu26	150.62 (12)	S23—Cu47A—Cu6	95.7 (4)
Cu29B—Cu6—Cu26	68.2 (5)	Cu55—Cu47A—Cu6	141.1 (5)
S7—Cu6—Cu35	52.77 (13)	S22—Cu47A—Cu6	164.1 (5)
S11—Cu6—Cu35	153.06 (14)	Cu35—Cu47A—Cu6	57.8 (2)
S6—Cu6—Cu35	78.19 (13)	S6—Cu47A—Cu50	99.5 (4)
Cu29A—Cu6—Cu35	133.80 (17)	S26—Cu47A—Cu50	48.4 (3)
Cu29B^iv^—Cu6—Cu35	96.1 (5)	S23—Cu47A—Cu50	89.0 (4)
Cu29A^iv^—Cu6—Cu35	87.85 (12)	Cu55—Cu47A—Cu50	96.3 (4)
Cu50—Cu6—Cu35	100.29 (10)	S22—Cu47A—Cu50	138.1 (4)
Cu29B—Cu6—Cu35	120.4 (5)	Cu35—Cu47A—Cu50	91.4 (4)
Cu26—Cu6—Cu35	59.29 (8)	Cu6—Cu47A—Cu50	54.7 (2)
S7—Cu6—Cu38	52.31 (12)	S6—Cu47B—S26	122.6 (8)
S11—Cu6—Cu38	76.78 (13)	S6—Cu47B—S22	129.1 (7)
S6—Cu6—Cu38	158.28 (14)	S26—Cu47B—S22	102.0 (6)
Cu29A—Cu6—Cu38	137.90 (18)	S6—Cu47B—Cu55	157.7 (7)
Cu29B^iv^—Cu6—Cu38	72.5 (5)	S26—Cu47B—Cu55	67.3 (4)
Cu29A^iv^—Cu6—Cu38	84.73 (14)	S22—Cu47B—Cu55	58.5 (4)
Cu50—Cu6—Cu38	60.08 (8)	S6—Cu47B—Cu46	97.5 (5)
Cu29B—Cu6—Cu38	151.1 (5)	S26—Cu47B—Cu46	97.8 (5)
Cu26—Cu6—Cu38	132.51 (11)	S22—Cu47B—Cu46	49.6 (3)
Cu35—Cu6—Cu38	88.20 (9)	Cu55—Cu47B—Cu46	100.7 (5)
S7—Cu6—Cu47A	109.3 (3)	S6—Cu47B—Cu49	98.6 (5)
S11—Cu6—Cu47A	103.5 (3)	S26—Cu47B—Cu49	48.5 (3)
S6—Cu6—Cu47A	49.1 (3)	S22—Cu47B—Cu49	93.3 (5)
Cu29A—Cu6—Cu47A	93.4 (3)	Cu55—Cu47B—Cu49	101.9 (5)
Cu29A^iv^—Cu6—Cu47A	143.7 (3)	Cu46—Cu47B—Cu49	58.1 (3)
Cu50—Cu6—Cu47A	65.8 (3)	S6—Cu47B—Cu5	48.9 (3)
Cu26—Cu6—Cu47A	85.1 (3)	S26—Cu47B—Cu5	101.8 (6)
Cu35—Cu6—Cu47A	60.4 (3)	S22—Cu47B—Cu5	102.8 (5)
Cu38—Cu6—Cu47A	109.3 (2)	Cu55—Cu47B—Cu5	153.1 (6)
S10—Cu7—S9^iii^	112.87 (17)	Cu46—Cu47B—Cu5	55.0 (2)
S10—Cu7—S13	114.61 (18)	Cu49—Cu47B—Cu5	57.2 (3)
S9^iii^—Cu7—S13	131.38 (18)	S9^iii^—Cu48—S25	121.44 (17)
S10—Cu7—Cu40	154.48 (15)	S9^iii^—Cu48—S29^iii^	118.93 (17)
S9^iii^—Cu7—Cu40	84.69 (13)	S25—Cu48—S29^iii^	101.40 (16)
S13—Cu7—Cu40	55.21 (12)	S9^iii^—Cu48—Cu16^iii^	114.57 (15)
S10—Cu7—Cu28	65.19 (13)	S25—Cu48—Cu16^iii^	54.53 (13)
S9^iii^—Cu7—Cu28	54.42 (12)	S29^iii^—Cu48—Cu16^iii^	125.78 (13)
S13—Cu7—Cu28	145.77 (14)	S9^iii^—Cu48—Cu18	118.99 (15)
Cu40—Cu7—Cu28	137.76 (11)	S25—Cu48—Cu18	55.95 (13)
S10—Cu7—Cu24^iv^	103.31 (14)	S29^iii^—Cu48—Cu18	53.03 (12)
S9^iii^—Cu7—Cu24^iv^	106.09 (13)	Cu16^iii^—Cu48—Cu18	106.60 (11)
S13—Cu7—Cu24^iv^	52.99 (11)	S9^iii^—Cu48—S28^iii^	115.36 (17)
Cu40—Cu7—Cu24^iv^	88.36 (9)	S25—Cu48—S28^iii^	98.47 (16)
Cu28—Cu7—Cu24^iv^	92.97 (10)	S29^iii^—Cu48—S28^iii^	96.73 (15)
S10—Cu7—Cu31^iii^	145.21 (14)	Cu16^iii^—Cu48—S28^iii^	50.97 (12)
S9^iii^—Cu7—Cu31^iii^	54.12 (12)	Cu18—Cu48—S28^iii^	125.48 (13)
S13—Cu7—Cu31^iii^	80.41 (13)	S9^iii^—Cu48—Cu20^iii^	117.60 (15)
Cu40—Cu7—Cu31^iii^	60.06 (8)	S25—Cu48—Cu20^iii^	120.74 (13)
Cu28—Cu7—Cu31^iii^	84.69 (9)	S29^iii^—Cu48—Cu20^iii^	50.76 (12)
Cu24^iv^—Cu7—Cu31^iii^	59.32 (8)	Cu16^iii^—Cu48—Cu20^iii^	96.75 (10)
S10—Cu7—Cu48	106.11 (14)	Cu18—Cu48—Cu20^iii^	99.09 (10)
S9^iii^—Cu7—Cu48	49.92 (11)	S28^iii^—Cu48—Cu20^iii^	49.73 (12)
S13—Cu7—Cu48	122.26 (14)	S9^iii^—Cu48—Cu7	51.45 (12)
Cu40—Cu7—Cu48	70.28 (9)	S25—Cu48—Cu7	94.02 (13)
Cu28—Cu7—Cu48	87.80 (9)	S29^iii^—Cu48—Cu7	87.38 (13)
Cu24^iv^—Cu7—Cu48	147.85 (11)	Cu16^iii^—Cu48—Cu7	135.45 (12)
Cu31^iii^—Cu7—Cu48	88.82 (9)	Cu18—Cu48—Cu7	67.56 (9)
S10—Cu7—Cu41	71.47 (13)	S28^iii^—Cu48—Cu7	165.79 (14)
S9^iii^—Cu7—Cu41	165.26 (14)	Cu20^iii^—Cu48—Cu7	127.69 (12)
S13—Cu7—Cu41	49.95 (12)	S9^iii^—Cu48—Cu4^iii^	48.56 (12)
Cu40—Cu7—Cu41	87.11 (9)	S25—Cu48—Cu4^iii^	90.91 (13)
Cu28—Cu7—Cu41	135.12 (11)	S29^iii^—Cu48—Cu4^iii^	166.55 (14)
Cu24^iv^—Cu7—Cu41	85.86 (9)	Cu16^iii^—Cu48—Cu4^iii^	66.08 (9)
Cu31^iii^—Cu7—Cu41	130.35 (10)	Cu18—Cu48—Cu4^iii^	133.96 (11)
Cu48—Cu7—Cu41	115.61 (9)	S28^iii^—Cu48—Cu4^iii^	86.67 (12)
S10—Cu8—S11	126.73 (19)	Cu20^iii^—Cu48—Cu4^iii^	126.50 (11)
S10—Cu8—S14	121.34 (19)	Cu7—Cu48—Cu4^iii^	86.37 (8)
S11—Cu8—S14	111.90 (18)	S26—Cu49—S10	130.46 (19)
S10—Cu8—Cu42	147.53 (16)	S26—Cu49—S25	119.09 (18)
S11—Cu8—Cu42	66.39 (14)	S10—Cu49—S25	110.08 (17)
S14—Cu8—Cu42	55.52 (13)	S26—Cu49—Cu46	102.71 (16)
S10—Cu8—Cu41	74.32 (14)	S10—Cu49—Cu46	100.52 (14)
S11—Cu8—Cu41	147.73 (15)	S25—Cu49—Cu46	54.56 (13)
S14—Cu8—Cu41	53.00 (12)	S26—Cu49—Cu5	110.12 (15)
Cu42—Cu8—Cu41	83.40 (11)	S10—Cu49—Cu5	51.62 (13)
S10—Cu8—Cu29A	76.42 (16)	S25—Cu49—Cu5	101.19 (14)
S11—Cu8—Cu29A	59.46 (15)	Cu46—Cu49—Cu5	57.58 (8)
S14—Cu8—Cu29A	146.07 (15)	S26—Cu49—Cu18	123.19 (15)
Cu42—Cu8—Cu29A	125.71 (14)	S10—Cu49—Cu18	90.51 (14)
Cu41—Cu8—Cu29A	150.14 (14)	S25—Cu49—Cu18	53.12 (13)
S10—Cu8—Cu23^iv^	108.35 (15)	Cu46—Cu49—Cu18	106.08 (11)
S11—Cu8—Cu23^iv^	105.92 (14)	Cu5—Cu49—Cu18	126.68 (10)
S14—Cu8—Cu23^iv^	50.75 (11)	S26—Cu49—Cu19	50.42 (13)
Cu42—Cu8—Cu23^iv^	92.98 (11)	S10—Cu49—Cu19	105.83 (15)
Cu41—Cu8—Cu23^iv^	85.89 (9)	S25—Cu49—Cu19	123.87 (14)
Cu29A—Cu8—Cu23^iv^	97.77 (11)	Cu46—Cu49—Cu19	151.08 (11)
S10—Cu8—Cu27^iv^	50.57 (12)	Cu5—Cu49—Cu19	134.94 (10)
S11—Cu8—Cu27^iv^	109.88 (14)	Cu18—Cu49—Cu19	85.61 (9)
S14—Cu8—Cu27^iv^	113.38 (14)	S26—Cu49—Cu47B	51.7 (3)
Cu42—Cu8—Cu27^iv^	160.70 (13)	S10—Cu49—Cu47B	107.9 (3)
Cu41—Cu8—Cu27^iv^	102.37 (10)	S25—Cu49—Cu47B	108.5 (3)
Cu29A—Cu8—Cu27^iv^	52.82 (10)	Cu46—Cu49—Cu47B	60.6 (3)
Cu23^iv^—Cu8—Cu27^iv^	69.37 (9)	Cu5—Cu49—Cu47B	62.8 (3)
S11—Cu9—S15	123.74 (19)	Cu18—Cu49—Cu47B	158.5 (3)
S11—Cu9—S12	120.40 (18)	Cu19—Cu49—Cu47B	99.4 (3)
S15—Cu9—S12	115.39 (18)	S11—Cu50—S26	116.56 (18)
S11—Cu9—Cu22^iv^	102.46 (13)	S11—Cu50—S27	127.28 (18)
S15—Cu9—Cu22^iv^	53.03 (12)	S26—Cu50—S27	115.94 (18)
S12—Cu9—Cu22^iv^	106.80 (13)	S11—Cu50—Cu57	135.92 (17)
S11—Cu9—Cu26^iv^	52.66 (12)	S26—Cu50—Cu57	83.56 (13)
S15—Cu9—Cu26^iv^	108.17 (14)	S27—Cu50—Cu57	55.31 (12)
S12—Cu9—Cu26^iv^	103.62 (13)	S11—Cu50—Cu6	54.82 (13)
Cu22^iv^—Cu9—Cu26^iv^	59.26 (8)	S26—Cu50—Cu6	105.77 (15)
S11—Cu9—Cu42	62.40 (14)	S27—Cu50—Cu6	105.83 (14)
S15—Cu9—Cu42	70.98 (14)	Cu57—Cu50—Cu6	161.03 (12)
S12—Cu9—Cu42	154.11 (14)	S11—Cu50—Cu38	78.32 (13)
Cu22^iv^—Cu9—Cu42	96.87 (10)	S26—Cu50—Cu38	151.34 (17)
Cu26^iv^—Cu9—Cu42	97.36 (9)	S27—Cu50—Cu38	52.59 (12)
S11—Cu9—Cu25^iv^	106.96 (14)	Cu57—Cu50—Cu38	102.80 (10)
S15—Cu9—Cu25^iv^	100.44 (14)	Cu6—Cu50—Cu38	61.18 (8)
S12—Cu9—Cu25^iv^	52.72 (11)	S11—Cu50—Cu19	98.13 (15)
Cu22^iv^—Cu9—Cu25^iv^	59.85 (8)	S26—Cu50—Cu19	52.59 (13)
Cu26^iv^—Cu9—Cu25^iv^	60.26 (8)	S27—Cu50—Cu19	117.20 (14)
Cu42—Cu9—Cu25^iv^	153.13 (11)	Cu57—Cu50—Cu19	61.90 (8)
S11—Cu9—Cu43	155.01 (14)	Cu6—Cu50—Cu19	136.82 (11)
S15—Cu9—Cu43	72.82 (14)	Cu38—Cu50—Cu19	154.13 (12)
S12—Cu9—Cu43	50.90 (12)	S11—Cu50—Cu47A	99.9 (2)
Cu22^iv^—Cu9—Cu43	102.52 (9)	S26—Cu50—Cu47A	50.0 (2)
Cu26^iv^—Cu9—Cu43	145.87 (10)	S27—Cu50—Cu47A	109.7 (3)
Cu42—Cu9—Cu43	114.27 (10)	Cu57—Cu50—Cu47A	121.3 (2)
Cu25^iv^—Cu9—Cu43	85.76 (8)	Cu6—Cu50—Cu47A	59.4 (2)
S11—Cu9—Cu51A	117.6 (2)	Cu38—Cu50—Cu47A	105.4 (2)
S15—Cu9—Cu51A	48.91 (18)	Cu19—Cu50—Cu47A	100.4 (2)
S12—Cu9—Cu51A	105.5 (2)	S15—Cu51A—Cu12	117.7 (4)
Cu22^iv^—Cu9—Cu51A	101.92 (16)	S15—Cu51A—S32	128.0 (4)
Cu26^iv^—Cu9—Cu51A	149.1 (2)	Cu12—Cu51A—S32	53.5 (3)
Cu42—Cu9—Cu51A	58.33 (19)	S15—Cu51A—S19^vi^	110.5 (3)
Cu25^iv^—Cu9—Cu51A	134.84 (19)	Cu12—Cu51A—S19^vi^	53.8 (2)
Cu43—Cu9—Cu51A	56.38 (19)	S32—Cu51A—S19^vi^	100.5 (3)
S11—Cu9—Cu38	75.19 (13)	S15—Cu51A—S31	121.8 (4)
S15—Cu9—Cu38	156.41 (15)	Cu12—Cu51A—S31	119.0 (3)
S12—Cu9—Cu38	50.65 (12)	S32—Cu51A—S31	96.5 (3)
Cu22^iv^—Cu9—Cu38	142.97 (10)	S19^vi^—Cu51A—S31	92.5 (3)
Cu26^iv^—Cu9—Cu38	94.54 (9)	S15—Cu51A—Cu60A	175.3 (4)
Cu42—Cu9—Cu38	113.23 (10)	Cu12—Cu51A—Cu60A	66.8 (3)
Cu25^iv^—Cu9—Cu38	85.05 (9)	S32—Cu51A—Cu60A	52.7 (2)
Cu43—Cu9—Cu38	84.86 (9)	S19^vi^—Cu51A—Cu60A	73.1 (3)
Cu51A—Cu9—Cu38	111.91 (17)	S31—Cu51A—Cu60A	54.38 (19)
S20^vi^—Cu10—S17^vii^	126.68 (18)	S15—Cu51A—Cu43	77.1 (3)
S20^vi^—Cu10—S29	116.99 (18)	Cu12—Cu51A—Cu43	75.9 (3)
S17^vii^—Cu10—S29	116.15 (18)	S32—Cu51A—Cu43	51.0 (2)
S20^vi^—Cu10—Cu40^v^	105.08 (13)	S19^vi^—Cu51A—Cu43	127.1 (4)
S17^vii^—Cu10—Cu40^v^	101.65 (13)	S31—Cu51A—Cu43	129.1 (3)
S29—Cu10—Cu40^v^	54.50 (12)	Cu60A—Cu51A—Cu43	103.3 (3)
S20^vi^—Cu10—Cu32^vii^	103.44 (14)	S15—Cu51A—Cu42	72.6 (3)
S17^vii^—Cu10—Cu32^vii^	53.46 (12)	Cu12—Cu51A—Cu42	163.9 (4)
S29—Cu10—Cu32^vii^	109.76 (14)	S32—Cu51A—Cu42	131.7 (3)
Cu40^v^—Cu10—Cu32^vii^	61.16 (8)	S19^vi^—Cu51A—Cu42	112.1 (3)
S20^vi^—Cu10—Cu56^viii^	111.56 (14)	S31—Cu51A—Cu42	49.19 (19)
S17^vii^—Cu10—Cu56^viii^	52.44 (12)	Cu60A—Cu51A—Cu42	103.5 (3)
S29—Cu10—Cu56^viii^	109.09 (14)	Cu43—Cu51A—Cu42	119.7 (3)
Cu40^v^—Cu10—Cu56^viii^	143.15 (11)	S15—Cu51A—Cu9	51.67 (18)
Cu32^vii^—Cu10—Cu56^viii^	105.24 (10)	Cu12—Cu51A—Cu9	137.0 (4)
S20^vi^—Cu10—Cu34^vi^	53.38 (12)	S32—Cu51A—Cu9	97.4 (3)
S17^vii^—Cu10—Cu34^vi^	109.44 (13)	S19^vi^—Cu51A—Cu9	160.7 (3)
S29—Cu10—Cu34^vi^	103.53 (13)	S31—Cu51A—Cu9	92.5 (3)
Cu40^v^—Cu10—Cu34^vi^	58.75 (8)	Cu60A—Cu51A—Cu9	124.3 (3)
Cu32^vii^—Cu10—Cu34^vi^	59.29 (7)	Cu43—Cu51A—Cu9	61.24 (17)
Cu56^viii^—Cu10—Cu34^vi^	147.21 (11)	Cu42—Cu51A—Cu9	58.98 (17)
S20^vi^—Cu10—Cu61	53.93 (14)	S19^vi^—Cu51B—S15	119.0 (5)
S17^vii^—Cu10—Cu61	154.86 (14)	S19^vi^—Cu51B—S31	108.3 (4)
S29—Cu10—Cu61	71.78 (14)	S15—Cu51B—S31	131.3 (5)
Cu40^v^—Cu10—Cu61	101.88 (10)	S19^vi^—Cu51B—Cu42	141.6 (6)
Cu32^vii^—Cu10—Cu61	149.48 (11)	S15—Cu51B—Cu42	78.7 (3)
Cu56^viii^—Cu10—Cu61	102.64 (10)	S31—Cu51B—Cu42	56.3 (3)
Cu34^vi^—Cu10—Cu61	90.37 (10)	S19^vi^—Cu51B—Cu60B	61.4 (4)
S17^vi^—Cu11—S30	120.37 (19)	S15—Cu51B—Cu60B	158.1 (6)
S17^vi^—Cu11—S18^vi^	122.06 (19)	S31—Cu51B—Cu60B	58.9 (4)
S30—Cu11—S18^vi^	116.73 (18)	Cu42—Cu51B—Cu60B	115.2 (5)
S17^vi^—Cu11—Cu59	158.95 (15)	S19^vi^—Cu51B—Cu12	52.1 (3)
S30—Cu11—Cu59	72.47 (14)	S15—Cu51B—Cu12	102.5 (5)
S18^vi^—Cu11—Cu59	52.26 (13)	S31—Cu51B—Cu12	115.5 (4)
S17^vi^—Cu11—Cu41	101.87 (14)	Cu42—Cu51B—Cu12	163.5 (5)
S30—Cu11—Cu41	52.15 (12)	Cu60B—Cu51B—Cu12	59.1 (4)
S18^vi^—Cu11—Cu41	104.95 (13)	S19^vi^—Cu51B—Cu1^vi^	102.2 (4)
Cu59—Cu11—Cu41	99.14 (10)	S15—Cu51B—Cu1^vi^	51.1 (2)
S17^vi^—Cu11—Cu52^vi^	75.77 (14)	S31—Cu51B—Cu1^vi^	110.1 (5)
S30—Cu11—Cu52^vi^	159.21 (15)	Cu42—Cu51B—Cu1^vi^	60.7 (3)
S18^vi^—Cu11—Cu52^vi^	52.15 (12)	Cu12—Cu51B—Cu1^vi^	132.7 (5)
Cu59—Cu11—Cu52^vi^	88.40 (10)	S19^vi^—Cu51B—Cu44^vi^	51.1 (3)
Cu41—Cu11—Cu52^vi^	142.32 (11)	S15—Cu51B—Cu44^vi^	98.5 (4)
S17^vi^—Cu11—Cu33^vi^	104.24 (14)	S31—Cu51B—Cu44^vi^	102.5 (4)
S30—Cu11—Cu33^vi^	104.74 (14)	Cu42—Cu51B—Cu44^vi^	95.0 (4)
S18^vi^—Cu11—Cu33^vi^	50.66 (11)	Cu12—Cu51B—Cu44^vi^	101.1 (4)
Cu59—Cu11—Cu33^vi^	86.72 (9)	Cu1^vi^—Cu51B—Cu44^vi^	56.0 (2)
Cu41—Cu11—Cu33^vi^	62.25 (8)	S18—Cu52—S24^xiii^	120.90 (19)
Cu52^vi^—Cu11—Cu33^vi^	81.60 (9)	S18—Cu52—S22	122.88 (19)
S17^vi^—Cu11—Cu62B^vi^	58.7 (3)	S24^xiii^—Cu52—S22	114.8 (2)
S30—Cu11—Cu62B^vi^	146.6 (3)	S18—Cu52—Cu62B	78.1 (3)
S18^vi^—Cu11—Cu62B^vi^	68.7 (3)	S24^xiii^—Cu52—Cu62B	142.1 (4)
Cu59—Cu11—Cu62B^vi^	121.0 (3)	S22—Cu52—Cu62B	65.4 (4)
Cu41—Cu11—Cu62B^vi^	94.5 (3)	S18—Cu52—Cu53	54.54 (13)
Cu52^vi^—Cu11—Cu62B^vi^	51.5 (3)	S24^xiii^—Cu52—Cu53	103.72 (14)
Cu33^vi^—Cu11—Cu62B^vi^	51.0 (3)	S22—Cu52—Cu53	101.66 (15)
S17^vi^—Cu11—Cu17^ix^	91.94 (13)	Cu62B—Cu52—Cu53	113.5 (4)
S30—Cu11—Cu17^ix^	93.99 (13)	S18—Cu52—Cu62A	82.72 (14)
S18^vi^—Cu11—Cu17^ix^	93.28 (12)	S24^xiii^—Cu52—Cu62A	152.50 (17)
Cu59—Cu11—Cu17^ix^	69.53 (8)	S22—Cu52—Cu62A	49.01 (15)
Cu41—Cu11—Cu17^ix^	145.91 (11)	Cu53—Cu52—Cu62A	101.74 (12)
Cu52^vi^—Cu11—Cu17^ix^	71.16 (8)	S18—Cu52—Cu15^xiii^	105.50 (14)
Cu33^vi^—Cu11—Cu17^ix^	143.79 (10)	S24^xiii^—Cu52—Cu15^xiii^	52.24 (12)
S32—Cu12—Cu45A^vi^	155.4 (3)	S22—Cu52—Cu15^xiii^	98.02 (15)
S32—Cu12—S19^vi^	123.0 (3)	Cu53—Cu52—Cu15^xiii^	58.39 (8)
Cu45A^vi^—Cu12—S19^vi^	62.9 (3)	Cu62A—Cu52—Cu15^xiii^	140.14 (14)
S32—Cu12—S20^vi^	118.0 (3)	S18—Cu52—Cu11^i^	53.67 (13)
Cu45A^vi^—Cu12—S20^vi^	66.0 (3)	S24^xiii^—Cu52—Cu11^i^	96.58 (14)
S19^vi^—Cu12—S20^vi^	118.2 (3)	S22—Cu52—Cu11^i^	131.61 (16)
S32—Cu12—Cu61	61.7 (2)	Cu53—Cu52—Cu11^i^	105.62 (11)
Cu45A^vi^—Cu12—Cu61	131.0 (4)	Cu62A—Cu52—Cu11^i^	86.38 (11)
S19^vi^—Cu12—Cu61	141.5 (3)	Cu15^xiii^—Cu52—Cu11^i^	130.31 (11)
S20^vi^—Cu12—Cu61	65.4 (2)	S18—Cu52—Cu13	126.84 (15)
S32—Cu12—Cu51A	66.6 (3)	S24^xiii^—Cu52—Cu13	94.51 (15)
Cu45A^vi^—Cu12—Cu51A	99.2 (3)	S22—Cu52—Cu13	56.43 (14)
S19^vi^—Cu12—Cu51A	67.4 (3)	Cu62B—Cu52—Cu13	52.3 (3)
S20^vi^—Cu12—Cu51A	153.9 (4)	Cu53—Cu52—Cu13	156.41 (12)
Cu61—Cu12—Cu51A	128.0 (3)	Cu62A—Cu52—Cu13	58.26 (10)
S32—Cu12—Cu60B	69.0 (4)	Cu15^xiii^—Cu52—Cu13	127.65 (11)
S19^vi^—Cu12—Cu60B	60.7 (4)	Cu11^i^—Cu52—Cu13	86.70 (10)
S20^vi^—Cu12—Cu60B	144.0 (4)	S21^x^—Cu53—S23	122.59 (19)
Cu61—Cu12—Cu60B	93.5 (4)	S21^x^—Cu53—S18	120.59 (19)
S32—Cu12—Cu45B^vi^	150.9 (7)	S23—Cu53—S18	116.79 (17)
Cu45A^vi^—Cu12—Cu45B^vi^	12.7 (7)	S21^x^—Cu53—Cu44	138.96 (16)
S19^vi^—Cu12—Cu45B^vi^	52.0 (8)	S23—Cu53—Cu44	78.56 (13)
S20^vi^—Cu12—Cu45B^vi^	78.6 (8)	S18—Cu53—Cu44	54.78 (12)
Cu61—Cu12—Cu45B^vi^	143.2 (8)	S21^x^—Cu53—Cu15^xiii^	54.56 (13)
Cu51A—Cu12—Cu45B^vi^	88.1 (8)	S23—Cu53—Cu15^xiii^	107.06 (15)
S32—Cu12—Cu54B^viii^	121.7 (4)	S18—Cu53—Cu15^xiii^	106.76 (14)
Cu45A^vi^—Cu12—Cu54B^viii^	81.7 (4)	Cu44—Cu53—Cu15^xiii^	159.78 (12)
S19^vi^—Cu12—Cu54B^viii^	53.0 (4)	S21^x^—Cu53—Cu52	107.74 (15)
S20^vi^—Cu12—Cu54B^viii^	87.9 (4)	S23—Cu53—Cu52	104.57 (15)
Cu61—Cu12—Cu54B^viii^	90.7 (4)	S18—Cu53—Cu52	52.74 (12)
Cu51A—Cu12—Cu54B^viii^	112.1 (5)	Cu44—Cu53—Cu52	98.28 (10)
S32—Cu12—Cu51B	78.2 (3)	Cu15^xiii^—Cu53—Cu52	61.59 (8)
S19^vi^—Cu12—Cu51B	53.2 (3)	S21^x^—Cu53—Cu14	98.69 (14)
S20^vi^—Cu12—Cu51B	155.2 (4)	S23—Cu53—Cu14	54.19 (13)
Cu61—Cu12—Cu51B	137.5 (4)	S18—Cu53—Cu14	117.55 (14)
Cu60B—Cu12—Cu51B	57.0 (3)	Cu44—Cu53—Cu14	63.56 (8)
Cu45B^vi^—Cu12—Cu51B	79.3 (8)	Cu15^xiii^—Cu53—Cu14	135.67 (11)
Cu54B^viii^—Cu12—Cu51B	99.3 (5)	Cu52—Cu53—Cu14	152.91 (11)
S32—Cu12—Cu60A	53.5 (2)	S21^x^—Cu53—Cu55	102.09 (15)
Cu45A^vi^—Cu12—Cu60A	138.6 (4)	S23—Cu53—Cu55	50.39 (13)
S19^vi^—Cu12—Cu60A	75.7 (3)	S18—Cu53—Cu55	113.91 (15)
S20^vi^—Cu12—Cu60A	143.8 (3)	Cu44—Cu53—Cu55	117.02 (11)
Cu61—Cu12—Cu60A	83.2 (2)	Cu15^xiii^—Cu53—Cu55	59.57 (8)
Cu51A—Cu12—Cu60A	61.2 (2)	Cu52—Cu53—Cu55	68.25 (10)
S17—Cu13—S21	121.6 (2)	Cu14—Cu53—Cu55	100.91 (11)
S17—Cu13—Cu62B	69.1 (5)	S19^xiii^—Cu54A—S22	123.2 (3)
S21—Cu13—Cu62B	142.2 (4)	S19^xiii^—Cu54A—Cu46	172.1 (3)
S17—Cu13—S22	122.09 (19)	S22—Cu54A—Cu46	53.47 (15)
S21—Cu13—S22	116.29 (19)	S19^xiii^—Cu54A—S25	121.4 (2)
Cu62B—Cu13—S22	64.8 (4)	S22—Cu54A—S25	98.68 (19)
S17—Cu13—Cu54A	154.19 (18)	Cu46—Cu54A—S25	55.57 (13)
S21—Cu13—Cu54A	67.17 (17)	S19^xiii^—Cu54A—Cu13	117.5 (2)
S22—Cu13—Cu54A	56.59 (16)	S22—Cu54A—Cu13	56.70 (15)
S17—Cu13—Cu62A	89.77 (16)	Cu46—Cu54A—Cu13	67.70 (12)
S21—Cu13—Cu62A	130.95 (18)	S25—Cu54A—Cu13	119.68 (16)
S22—Cu13—Cu62A	48.42 (14)	S19^xiii^—Cu54A—Cu16^iii^	117.1 (2)
Cu54A—Cu13—Cu62A	101.56 (15)	S22—Cu54A—Cu16^iii^	119.34 (17)
S17—Cu13—Cu54B	138.4 (4)	Cu46—Cu54A—Cu16^iii^	67.54 (12)
S21—Cu13—Cu54B	87.2 (4)	S25—Cu54A—Cu16^iii^	51.41 (14)
Cu62B—Cu13—Cu54B	107.7 (5)	Cu13—Cu54A—Cu16^iii^	90.84 (15)
S22—Cu13—Cu54B	43.5 (3)	S19^xiii^—Cu54A—S21	113.5 (2)
S17—Cu13—Cu52	74.98 (14)	S22—Cu54A—S21	99.63 (19)
S21—Cu13—Cu52	156.28 (17)	Cu46—Cu54A—S21	74.38 (15)
Cu62B—Cu13—Cu52	56.4 (3)	S25—Cu54A—S21	95.07 (19)
S22—Cu13—Cu52	50.96 (13)	Cu13—Cu54A—S21	48.29 (13)
Cu54A—Cu13—Cu52	90.73 (14)	Cu16^iii^—Cu54A—S21	47.51 (14)
Cu62A—Cu13—Cu52	59.61 (10)	S19^xiii^—Cu54A—Cu60A^ix^	73.67 (18)
Cu54B—Cu13—Cu52	70.3 (4)	S22—Cu54A—Cu60A^ix^	122.7 (2)
S17—Cu13—Cu46	148.03 (16)	Cu46—Cu54A—Cu60A^ix^	101.81 (18)
S21—Cu13—Cu46	75.80 (15)	S25—Cu54A—Cu60A^ix^	48.43 (15)
Cu62B—Cu13—Cu46	81.2 (5)	Cu13—Cu54A—Cu60A^ix^	167.86 (19)
S22—Cu13—Cu46	48.63 (13)	Cu16^iii^—Cu54A—Cu60A^ix^	79.12 (16)
Cu54A—Cu13—Cu46	54.18 (10)	S21—Cu54A—Cu60A^ix^	124.4 (2)
Cu62A—Cu13—Cu46	61.26 (12)	S19^xiii^—Cu54A—Cu14^xiii^	50.51 (14)
Cu54B—Cu13—Cu46	61.4 (3)	S22—Cu54A—Cu14^xiii^	92.29 (19)
Cu52—Cu13—Cu46	98.73 (11)	Cu46—Cu54A—Cu14^xiii^	133.60 (17)
S24—Cu14—S19	119.67 (18)	S25—Cu54A—Cu14^xiii^	169.0 (2)
S24—Cu14—S23	116.30 (18)	Cu13—Cu54A—Cu14^xiii^	67.10 (12)
S19—Cu14—S23	123.10 (18)	Cu16^iii^—Cu54A—Cu14^xiii^	122.79 (18)
S24—Cu14—Cu45B	80.1 (7)	S21—Cu54A—Cu14^xiii^	83.48 (16)
S19—Cu14—Cu45B	52.7 (7)	Cu60A^ix^—Cu54A—Cu14^xiii^	124.18 (15)
S23—Cu14—Cu45B	132.9 (10)	S22—Cu54B—S19^xiii^	168.6 (9)
S24—Cu14—Cu54B^x^	90.5 (4)	S22—Cu54B—Cu12^ix^	125.6 (8)
S19—Cu14—Cu54B^x^	51.7 (3)	S19^xiii^—Cu54B—Cu12^ix^	53.3 (3)
S23—Cu14—Cu54B^x^	137.5 (4)	S22—Cu54B—Cu14^xiii^	115.9 (7)
S24—Cu14—Cu53	152.39 (15)	S19^xiii^—Cu54B—Cu14^xiii^	55.6 (3)
S19—Cu14—Cu53	80.28 (14)	Cu12^ix^—Cu54B—Cu14^xiii^	96.1 (4)
S23—Cu14—Cu53	52.51 (13)	S22—Cu54B—Cu60B^ix^	131.9 (7)
Cu45B—Cu14—Cu53	126.9 (7)	S19^xiii^—Cu54B—Cu60B^ix^	58.6 (4)
Cu54B^x^—Cu14—Cu53	88.2 (4)	Cu12^ix^—Cu54B—Cu60B^ix^	57.7 (4)
S24—Cu14—Cu35	106.15 (14)	Cu14^xiii^—Cu54B—Cu60B^ix^	110.7 (6)
S19—Cu14—Cu35	102.93 (14)	S22—Cu54B—Cu13	61.2 (4)
S23—Cu14—Cu35	50.65 (12)	S19^xiii^—Cu54B—Cu13	117.6 (7)
Cu45B—Cu14—Cu35	82.9 (10)	Cu12^ix^—Cu54B—Cu13	167.2 (6)
Cu54B^x^—Cu14—Cu35	154.6 (3)	Cu14^xiii^—Cu54B—Cu13	71.2 (4)
Cu53—Cu14—Cu35	85.55 (9)	Cu60B^ix^—Cu54B—Cu13	128.1 (7)
S24—Cu14—Cu56	71.46 (13)	S22—Cu54B—Cu46	51.4 (3)
S19—Cu14—Cu56	162.78 (15)	S19^xiii^—Cu54B—Cu46	139.4 (8)
S23—Cu14—Cu56	51.06 (12)	Cu12^ix^—Cu54B—Cu46	129.6 (6)
Cu45B—Cu14—Cu56	144.4 (7)	Cu14^xiii^—Cu54B—Cu46	132.4 (6)
Cu54B^x^—Cu14—Cu56	119.0 (3)	Cu60B^ix^—Cu54B—Cu46	88.2 (5)
Cu53—Cu14—Cu56	85.14 (10)	Cu13—Cu54B—Cu46	63.2 (3)
Cu35—Cu14—Cu56	84.91 (9)	S20^xiii^—Cu55—S23	133.3 (2)
S24—Cu14—Cu45A	73.51 (16)	S20^xiii^—Cu55—S22	114.4 (2)
S19—Cu14—Cu45A	51.88 (14)	S23—Cu55—S22	107.20 (19)
S23—Cu14—Cu45A	145.9 (2)	S20^xiii^—Cu55—Cu47A	158.8 (3)
Cu53—Cu14—Cu45A	131.25 (13)	S23—Cu55—Cu47A	59.1 (3)
Cu35—Cu14—Cu45A	95.56 (17)	S22—Cu55—Cu47A	67.6 (3)
Cu56—Cu14—Cu45A	143.59 (13)	S20^xiii^—Cu55—Cu47B	152.1 (4)
S24—Cu14—Cu44	149.67 (15)	S23—Cu55—Cu47B	71.3 (3)
S19—Cu14—Cu44	52.31 (12)	S22—Cu55—Cu47B	55.7 (4)
S23—Cu14—Cu44	74.20 (13)	S20^xiii^—Cu55—Cu61^ix^	58.42 (14)
Cu45B—Cu14—Cu44	73.4 (8)	S23—Cu55—Cu61^ix^	131.7 (2)
Cu54B^x^—Cu14—Cu44	99.6 (3)	S22—Cu55—Cu61^ix^	102.19 (17)
Cu53—Cu14—Cu44	57.23 (8)	Cu47A—Cu55—Cu61^ix^	100.4 (3)
Cu35—Cu14—Cu44	56.71 (8)	S20^xiii^—Cu55—S26	106.56 (18)
Cu56—Cu14—Cu44	125.26 (11)	S23—Cu55—S26	94.01 (18)
Cu45A—Cu14—Cu44	82.98 (14)	S22—Cu55—S26	89.21 (17)
S24—Cu14—Cu54A^x^	108.33 (16)	Cu47A—Cu55—S26	52.4 (3)
S19—Cu14—Cu54A^x^	47.76 (13)	Cu47B—Cu55—S26	51.6 (4)
S23—Cu14—Cu54A^x^	120.52 (17)	Cu61^ix^—Cu55—S26	48.63 (13)
Cu53—Cu14—Cu54A^x^	69.58 (12)	S20^xiii^—Cu55—Cu15^xiii^	53.11 (13)
Cu35—Cu14—Cu54A^x^	143.18 (13)	S23—Cu55—Cu15^xiii^	104.61 (16)
Cu56—Cu14—Cu54A^x^	118.09 (11)	S22—Cu55—Cu15^xiii^	94.61 (17)
Cu45A—Cu14—Cu54A^x^	82.27 (15)	Cu47A—Cu55—Cu15^xiii^	147.3 (3)
Cu44—Cu14—Cu54A^x^	86.66 (11)	Cu61^ix^—Cu55—Cu15^xiii^	110.35 (12)
S24—Cu15—S20	120.64 (19)	S26—Cu55—Cu15^xiii^	158.88 (15)
S24—Cu15—S21^v^	120.39 (19)	S20^xiii^—Cu55—Cu53	104.77 (16)
S20—Cu15—S21^v^	118.97 (19)	S23—Cu55—Cu53	50.70 (13)
S24—Cu15—Cu53^x^	105.47 (14)	S22—Cu55—Cu53	94.01 (16)
S20—Cu15—Cu53^x^	109.50 (14)	Cu47A—Cu55—Cu53	95.9 (3)
S21^v^—Cu15—Cu53^x^	53.08 (12)	Cu47B—Cu55—Cu53	102.1 (4)
S24—Cu15—Cu37	106.01 (14)	Cu61^ix^—Cu55—Cu53	160.37 (14)
S20—Cu15—Cu37	108.57 (14)	S26—Cu55—Cu53	143.83 (14)
S21^v^—Cu15—Cu37	52.78 (12)	Cu15^xiii^—Cu55—Cu53	56.69 (9)
Cu53^x^—Cu15—Cu37	105.68 (10)	S23—Cu56—S17^x^	127.80 (18)
S24—Cu15—Cu55^x^	112.30 (15)	S23—Cu56—S27	113.04 (17)
S20—Cu15—Cu55^x^	51.18 (12)	S17^x^—Cu56—S27	117.59 (18)
S21^v^—Cu15—Cu55^x^	104.34 (15)	S23—Cu56—Cu10^ix^	103.98 (15)
Cu53^x^—Cu15—Cu55^x^	63.74 (9)	S17^x^—Cu56—Cu10^ix^	53.29 (12)
Cu37—Cu15—Cu55^x^	141.69 (12)	S27—Cu56—Cu10^ix^	103.14 (14)
S24—Cu15—Cu52^x^	52.58 (12)	S23—Cu56—Cu14	53.54 (13)
S20—Cu15—Cu52^x^	111.18 (14)	S17^x^—Cu56—Cu14	103.34 (15)
S21^v^—Cu15—Cu52^x^	105.12 (14)	S27—Cu56—Cu14	124.58 (14)
Cu53^x^—Cu15—Cu52^x^	60.02 (8)	Cu10^ix^—Cu56—Cu14	131.71 (11)
Cu37—Cu15—Cu52^x^	140.24 (11)	S23—Cu56—Cu17	124.31 (14)
Cu55^x^—Cu15—Cu52^x^	69.28 (10)	S17^x^—Cu56—Cu17	97.03 (14)
S24—Cu15—Cu36A	51.07 (16)	S27—Cu56—Cu17	52.25 (12)
S20—Cu15—Cu36A	105.7 (2)	Cu10^ix^—Cu56—Cu17	130.80 (11)
S21^v^—Cu15—Cu36A	111.22 (17)	Cu14—Cu56—Cu17	88.98 (10)
Cu53^x^—Cu15—Cu36A	144.58 (19)	S29^ix^—Cu57—S31	119.56 (18)
Cu37—Cu15—Cu36A	65.38 (10)	S29^ix^—Cu57—S27	122.84 (18)
Cu55^x^—Cu15—Cu36A	144.26 (15)	S31—Cu57—S27	117.39 (17)
Cu52^x^—Cu15—Cu36A	103.65 (14)	S29^ix^—Cu57—Cu50	136.20 (16)
S24—Cu15—Cu45A	73.40 (19)	S31—Cu57—Cu50	83.66 (13)
S20—Cu15—Cu45A	54.85 (19)	S27—Cu57—Cu50	54.73 (12)
S21^v^—Cu15—Cu45A	149.85 (15)	S29^ix^—Cu57—Cu58	106.99 (14)
Cu53^x^—Cu15—Cu45A	154.50 (13)	S31—Cu57—Cu58	104.71 (15)
Cu37—Cu15—Cu45A	98.92 (10)	S27—Cu57—Cu58	52.56 (12)
Cu55^x^—Cu15—Cu45A	92.64 (15)	Cu50—Cu57—Cu58	100.98 (10)
Cu52^x^—Cu15—Cu45A	104.14 (12)	S29^ix^—Cu57—Cu18^viii^	52.78 (13)
Cu36A—Cu15—Cu45A	54.0 (2)	S31—Cu57—Cu18^viii^	107.34 (14)
S21^v^—Cu16—S28	123.7 (2)	S27—Cu57—Cu18^viii^	105.38 (14)
S21^v^—Cu16—S25^v^	121.25 (19)	Cu50—Cu57—Cu18^viii^	160.03 (12)
S28—Cu16—S25^v^	113.94 (18)	Cu58—Cu57—Cu18^viii^	60.55 (8)
S21^v^—Cu16—Cu48^v^	159.87 (16)	S29^ix^—Cu57—Cu19	101.47 (14)
S28—Cu16—Cu48^v^	64.63 (14)	S31—Cu57—Cu19	53.22 (13)
S25^v^—Cu16—Cu48^v^	58.90 (13)	S27—Cu57—Cu19	115.88 (14)
S21^v^—Cu16—Cu54A^v^	66.70 (16)	Cu50—Cu57—Cu19	61.17 (8)
S28—Cu16—Cu54A^v^	142.30 (16)	Cu58—Cu57—Cu19	150.63 (12)
S25^v^—Cu16—Cu54A^v^	61.03 (15)	Cu18^viii^—Cu57—Cu19	138.74 (11)
Cu48^v^—Cu16—Cu54A^v^	119.61 (14)	S29^ix^—Cu57—Cu60A	102.51 (16)
S21^v^—Cu16—Cu59^viii^	105.71 (14)	S31—Cu57—Cu60A	53.13 (17)
S28—Cu16—Cu59^viii^	49.72 (12)	S27—Cu57—Cu60A	107.08 (18)
S25^v^—Cu16—Cu59^viii^	102.18 (14)	Cu50—Cu57—Cu60A	120.40 (13)
Cu48^v^—Cu16—Cu59^viii^	93.32 (10)	Cu58—Cu57—Cu60A	61.89 (15)
Cu54A^v^—Cu16—Cu59^viii^	93.24 (11)	Cu18^viii^—Cu57—Cu60A	59.86 (12)
S21^v^—Cu16—Cu46^v^	75.73 (14)	Cu19—Cu57—Cu60A	105.14 (16)
S28—Cu16—Cu46^v^	156.49 (16)	S27—Cu58—S30^viii^	126.18 (18)
S25^v^—Cu16—Cu46^v^	52.69 (13)	S27—Cu58—S32	116.77 (17)
Cu48^v^—Cu16—Cu46^v^	92.74 (10)	S30^viii^—Cu58—S32	116.33 (18)
Cu54A^v^—Cu16—Cu46^v^	53.68 (10)	S27—Cu58—Cu57	54.06 (12)
Cu59^viii^—Cu16—Cu46^v^	144.21 (11)	S30^viii^—Cu58—Cu57	106.79 (14)
S24—Cu17—S28	120.32 (19)	S32—Cu58—Cu57	101.52 (15)
S24—Cu17—S27	117.68 (18)	S27—Cu58—Cu17	53.04 (12)
S28—Cu17—S27	121.50 (18)	S30^viii^—Cu58—Cu17	101.04 (15)
S24—Cu17—Cu58	155.10 (15)	S32—Cu58—Cu17	124.80 (14)
S28—Cu17—Cu58	76.47 (14)	Cu57—Cu58—Cu17	104.84 (10)
S27—Cu17—Cu58	52.56 (12)	S27—Cu58—Cu18^viii^	106.62 (14)
S24—Cu17—Cu39	107.58 (14)	S30^viii^—Cu58—Cu18^viii^	50.84 (13)
S28—Cu17—Cu39	52.23 (12)	S32—Cu58—Cu18^viii^	104.68 (14)
S27—Cu17—Cu39	103.26 (14)	Cu57—Cu58—Cu18^viii^	60.51 (8)
Cu58—Cu17—Cu39	97.26 (10)	Cu17—Cu58—Cu18^viii^	130.51 (11)
S24—Cu17—Cu56	71.60 (14)	S27—Cu58—Cu20	124.44 (14)
S28—Cu17—Cu56	160.92 (15)	S30^viii^—Cu58—Cu20	95.21 (14)
S27—Cu17—Cu56	51.79 (12)	S32—Cu58—Cu20	51.94 (13)
Cu58—Cu17—Cu56	87.78 (9)	Cu57—Cu58—Cu20	151.86 (12)
Cu39—Cu17—Cu56	142.39 (11)	Cu17—Cu58—Cu20	87.44 (9)
S24—Cu17—Cu36B	49.3 (5)	Cu18^viii^—Cu58—Cu20	128.87 (10)
S28—Cu17—Cu36B	103.5 (5)	S27—Cu58—Cu60A	107.93 (16)
S27—Cu17—Cu36B	108.0 (5)	S30^viii^—Cu58—Cu60A	98.96 (16)
Cu58—Cu17—Cu36B	150.4 (5)	S32—Cu58—Cu60A	50.13 (15)
Cu39—Cu17—Cu36B	62.9 (5)	Cu57—Cu58—Cu60A	61.40 (12)
Cu56—Cu17—Cu36B	95.6 (4)	Cu17—Cu58—Cu60A	158.49 (13)
S24—Cu17—Cu38	106.41 (14)	Cu18^viii^—Cu58—Cu60A	59.69 (11)
S28—Cu17—Cu38	104.05 (14)	Cu20—Cu58—Cu60A	98.56 (13)
S27—Cu17—Cu38	51.14 (11)	S28^ix^—Cu59—S31	120.11 (19)
Cu58—Cu17—Cu38	84.81 (9)	S28^ix^—Cu59—S18^vi^	122.82 (19)
Cu39—Cu17—Cu38	58.76 (8)	S31—Cu59—S18^vi^	114.20 (18)
Cu56—Cu17—Cu38	84.86 (9)	S28^ix^—Cu59—Cu11	101.10 (15)
Cu36B—Cu17—Cu38	66.3 (5)	S31—Cu59—Cu11	127.31 (15)
S24—Cu17—Cu11^viii^	91.15 (13)	S18^vi^—Cu59—Cu11	54.25 (13)
S28—Cu17—Cu11^viii^	93.03 (13)	S28^ix^—Cu59—Cu19	103.81 (15)
S27—Cu17—Cu11^viii^	92.85 (12)	S31—Cu59—Cu19	51.68 (13)
Cu58—Cu17—Cu11^viii^	68.43 (8)	S18^vi^—Cu59—Cu19	123.64 (15)
Cu39—Cu17—Cu11^viii^	145.19 (11)	Cu11—Cu59—Cu19	89.69 (10)
Cu56—Cu17—Cu11^viii^	70.89 (8)	S28^ix^—Cu59—Cu16^ix^	49.72 (13)
Cu38—Cu17—Cu11^viii^	143.95 (10)	S31—Cu59—Cu16^ix^	103.59 (15)
S30—Cu18—S29^iii^	128.39 (18)	S18^vi^—Cu59—Cu16^ix^	102.20 (15)
S30—Cu18—S25	120.25 (18)	Cu11—Cu59—Cu16^ix^	128.64 (11)
S29^iii^—Cu18—S25	110.57 (18)	Cu19—Cu59—Cu16^ix^	133.17 (11)
S30—Cu18—Cu48	159.23 (15)	S25^viii^—Cu60A—S32	131.4 (3)
S29^iii^—Cu18—Cu48	61.43 (13)	S25^viii^—Cu60A—S31	117.6 (3)
S25—Cu18—Cu48	58.26 (13)	S32—Cu60A—S31	109.3 (2)
S30—Cu18—Cu57^ix^	107.01 (14)	S25^viii^—Cu60A—Cu51A	156.0 (4)
S29^iii^—Cu18—Cu57^ix^	51.86 (12)	S32—Cu60A—Cu51A	60.9 (2)
S25—Cu18—Cu57^ix^	101.12 (13)	S31—Cu60A—Cu51A	62.3 (2)
Cu48—Cu18—Cu57^ix^	93.18 (10)	S25^viii^—Cu60A—Cu12	115.8 (3)
S30—Cu18—Cu58^ix^	52.67 (12)	S32—Cu60A—Cu12	50.61 (19)
S29^iii^—Cu18—Cu58^ix^	104.74 (13)	S31—Cu60A—Cu12	112.5 (2)
S25—Cu18—Cu58^ix^	107.83 (14)	Cu51A—Cu60A—Cu12	52.0 (2)
Cu48—Cu18—Cu58^ix^	147.61 (11)	S25^viii^—Cu60A—Cu54A^viii^	60.4 (2)
Cu57^ix^—Cu18—Cu58^ix^	58.95 (8)	S32—Cu60A—Cu54A^viii^	114.5 (3)
S30—Cu18—Cu60A^ix^	102.32 (18)	S31—Cu60A—Cu54A^viii^	110.0 (3)
S29^iii^—Cu18—Cu60A^ix^	103.34 (16)	Cu51A—Cu60A—Cu54A^viii^	96.2 (3)
S25—Cu18—Cu60A^ix^	49.84 (16)	Cu12—Cu60A—Cu54A^viii^	66.6 (2)
Cu48—Cu18—Cu60A^ix^	91.35 (15)	S25^viii^—Cu60A—Cu18^viii^	54.43 (16)
Cu57^ix^—Cu18—Cu60A^ix^	61.72 (12)	S32—Cu60A—Cu18^viii^	104.1 (3)
Cu58^ix^—Cu18—Cu60A^ix^	62.03 (14)	S31—Cu60A—Cu18^viii^	103.6 (2)
S30—Cu18—Cu49	74.64 (14)	Cu51A—Cu60A—Cu18^viii^	149.1 (3)
S29^iii^—Cu18—Cu49	153.74 (15)	Cu12—Cu60A—Cu18^viii^	141.0 (2)
S25—Cu18—Cu49	51.84 (12)	Cu54A^viii^—Cu60A—Cu18^viii^	114.65 (17)
Cu48—Cu18—Cu49	92.69 (10)	S25^viii^—Cu60A—Cu57	102.0 (2)
Cu57^ix^—Cu18—Cu49	141.53 (11)	S32—Cu60A—Cu57	98.1 (3)
Cu58^ix^—Cu18—Cu49	99.86 (10)	S31—Cu60A—Cu57	50.41 (16)
Cu60A^ix^—Cu18—Cu49	80.16 (13)	Cu51A—Cu60A—Cu57	95.2 (2)
S30—Cu19—S26	116.86 (19)	Cu12—Cu60A—Cu57	141.3 (2)
S30—Cu19—S31	115.28 (18)	Cu54A^viii^—Cu60A—Cu57	147.0 (2)
S26—Cu19—S31	127.84 (19)	Cu18^viii^—Cu60A—Cu57	58.42 (14)
S30—Cu19—Cu50	152.38 (15)	S25^viii^—Cu60A—Cu58	108.6 (3)
S26—Cu19—Cu50	52.93 (13)	S32—Cu60A—Cu58	50.89 (19)
S31—Cu19—Cu50	80.41 (14)	S31—Cu60A—Cu58	98.1 (2)
S30—Cu19—Cu57	150.67 (15)	Cu51A—Cu60A—Cu58	94.9 (2)
S26—Cu19—Cu57	80.62 (14)	Cu12—Cu60A—Cu58	101.1 (2)
S31—Cu19—Cu57	52.17 (13)	Cu54A^viii^—Cu60A—Cu58	151.8 (2)
Cu50—Cu19—Cu57	56.93 (8)	Cu18^viii^—Cu60A—Cu58	58.29 (16)
S30—Cu19—Cu42	101.28 (15)	Cu57—Cu60A—Cu58	56.71 (15)
S26—Cu19—Cu42	117.94 (15)	S25^viii^—Cu60B—S31	117.7 (5)
S31—Cu19—Cu42	50.55 (12)	S25^viii^—Cu60B—S19^vi^	128.7 (6)
Cu50—Cu19—Cu42	70.28 (10)	S31—Cu60B—S19^vi^	99.8 (4)
Cu57—Cu19—Cu42	88.98 (10)	S25^viii^—Cu60B—Cu51B	173.3 (7)
S30—Cu19—Cu59	69.70 (14)	S31—Cu60B—Cu51B	56.2 (4)
S26—Cu19—Cu59	158.92 (15)	S19^vi^—Cu60B—Cu51B	53.6 (4)
S31—Cu19—Cu59	49.89 (13)	S25^viii^—Cu60B—Cu12	122.8 (5)
Cu50—Cu19—Cu59	130.27 (11)	S31—Cu60B—Cu12	117.4 (4)
Cu57—Cu19—Cu59	86.09 (9)	S19^vi^—Cu60B—Cu12	52.1 (3)
Cu42—Cu19—Cu59	77.83 (10)	Cu51B—Cu60B—Cu12	63.9 (4)
S30—Cu19—Cu49	73.02 (14)	S25^viii^—Cu60B—Cu54B^viii^	81.7 (5)
S26—Cu19—Cu49	48.90 (13)	S31—Cu60B—Cu54B^viii^	146.4 (7)
S31—Cu19—Cu49	158.54 (14)	S19^vi^—Cu60B—Cu54B^viii^	50.2 (4)
Cu50—Cu19—Cu49	84.90 (9)	Cu12—Cu60B—Cu54B^viii^	59.9 (5)
Cu57—Cu19—Cu49	129.39 (11)	S25^viii^—Cu60B—S32	112.1 (6)
Cu42—Cu19—Cu49	109.57 (10)	S31—Cu60B—S32	95.5 (4)
Cu59—Cu19—Cu49	142.72 (11)	S19^vi^—Cu60B—S32	96.6 (4)
S28—Cu20—S29	117.55 (19)	Cu51B—Cu60B—S32	72.5 (4)
S28—Cu20—S32	121.43 (19)	Cu12—Cu60B—S32	48.5 (3)
S29—Cu20—S32	120.02 (18)	Cu54B^viii^—Cu60B—S32	102.1 (6)
S28—Cu20—Cu48^v^	62.94 (14)	S26^viii^—Cu61—Cu12	139.8 (3)
S29—Cu20—Cu48^v^	60.25 (13)	S26^viii^—Cu61—S32	128.8 (2)
S32—Cu20—Cu48^v^	146.74 (15)	Cu12—Cu61—S32	58.0 (2)
S28—Cu20—Cu61	157.88 (17)	S26^viii^—Cu61—S20^vi^	120.23 (19)
S29—Cu20—Cu61	75.95 (15)	Cu12—Cu61—S20^vi^	57.2 (2)
S32—Cu20—Cu61	52.77 (14)	S32—Cu61—S20^vi^	107.92 (19)
Cu48^v^—Cu20—Cu61	135.46 (13)	S26^viii^—Cu61—Cu55^viii^	68.48 (15)
S28—Cu20—Cu58	75.81 (14)	Cu12—Cu61—Cu55^viii^	91.6 (2)
S29—Cu20—Cu58	160.69 (16)	S32—Cu61—Cu55^viii^	148.6 (2)
S32—Cu20—Cu58	52.21 (13)	S20^vi^—Cu61—Cu55^viii^	52.38 (13)
Cu48^v^—Cu20—Cu58	137.26 (12)	S26^viii^—Cu61—Cu20	109.2 (2)
Cu61—Cu20—Cu58	87.28 (11)	Cu12—Cu61—Cu20	103.4 (2)
S28—Cu20—Cu43	106.60 (14)	S32—Cu61—Cu20	53.46 (14)
S29—Cu20—Cu43	103.94 (14)	S20^vi^—Cu61—Cu20	119.65 (15)
S32—Cu20—Cu43	49.67 (12)	Cu55^viii^—Cu61—Cu20	154.16 (18)
Cu48^v^—Cu20—Cu43	97.08 (9)	S26^viii^—Cu61—Cu10	102.81 (17)
Cu61—Cu20—Cu43	85.22 (11)	Cu12—Cu61—Cu10	100.8 (2)
Cu58—Cu20—Cu43	83.78 (9)	S32—Cu61—Cu10	121.80 (16)
S16^iv^—Cu21—S13^i^	132.65 (17)	S20^vi^—Cu61—Cu10	49.98 (13)
S16^iv^—Cu21—S2	118.96 (17)	Cu55^viii^—Cu61—Cu10	67.59 (11)
S13^i^—Cu21—S2	105.74 (16)	Cu20—Cu61—Cu10	88.73 (12)
S16^iv^—Cu21—Cu31^iv^	56.28 (12)	S22—Cu62A—S2	173.3 (3)
S13^i^—Cu21—Cu31^iv^	125.03 (14)	S22—Cu62A—Cu3	117.59 (18)
S2—Cu21—Cu31^iv^	107.50 (14)	S2—Cu62A—Cu3	56.57 (13)
S16^iv^—Cu21—Cu2^ii^	54.90 (12)	S22—Cu62A—Cu13	59.63 (15)
S13^i^—Cu21—Cu2^ii^	104.47 (13)	S2—Cu62A—Cu13	119.07 (19)
S2—Cu21—Cu2^ii^	100.34 (13)	Cu3—Cu62A—Cu13	76.41 (11)
Cu31^iv^—Cu21—Cu2^ii^	110.98 (9)	S22—Cu62A—Cu52	54.26 (15)
S16^iv^—Cu21—Cu32	141.99 (15)	S2—Cu62A—Cu52	131.8 (2)
S13^i^—Cu21—Cu32	55.78 (11)	Cu3—Cu62A—Cu52	135.45 (16)
S2—Cu21—Cu32	81.15 (12)	Cu13—Cu62A—Cu52	62.12 (10)
Cu31^iv^—Cu21—Cu32	87.95 (9)	S22—Cu62A—Cu33	133.45 (19)
Cu2^ii^—Cu21—Cu32	159.19 (11)	S2—Cu62A—Cu33	53.26 (13)
S16^iv^—Cu21—Cu24^ii^	81.87 (13)	Cu3—Cu62A—Cu33	104.34 (12)
S13^i^—Cu21—Cu24^ii^	52.42 (11)	Cu13—Cu62A—Cu33	116.79 (17)
S2—Cu21—Cu24^ii^	140.57 (14)	Cu52—Cu62A—Cu33	82.07 (12)
Cu31^iv^—Cu21—Cu24^ii^	111.81 (10)	S22—Cu62A—Cu46	51.06 (15)
Cu2^ii^—Cu21—Cu24^ii^	62.77 (8)	S2—Cu62A—Cu46	122.23 (17)
Cu32—Cu21—Cu24^ii^	102.94 (9)	Cu3—Cu62A—Cu46	71.03 (10)
S16^iv^—Cu21—Cu3	105.39 (13)	Cu13—Cu62A—Cu46	63.38 (10)
S13^i^—Cu21—Cu3	113.80 (13)	Cu52—Cu62A—Cu46	101.72 (11)
S2—Cu21—Cu3	53.14 (12)	Cu33—Cu62A—Cu46	175.31 (13)
Cu31^iv^—Cu21—Cu3	60.17 (8)	S2—Cu62B—Cu13	137.6 (6)
Cu2^ii^—Cu21—Cu3	137.70 (11)	S2—Cu62B—Cu52	150.9 (7)
Cu32—Cu21—Cu3	58.99 (8)	Cu13—Cu62B—Cu52	71.3 (3)
Cu24^ii^—Cu21—Cu3	158.77 (11)	S2—Cu62B—Cu33	59.3 (3)
S3—Cu22—S15^iv^	127.13 (18)	Cu13—Cu62B—Cu33	155.4 (9)
S3—Cu22—S2	121.72 (16)	Cu52—Cu62B—Cu33	95.3 (5)
S15^iv^—Cu22—S2	111.06 (17)	S2—Cu62B—S22	129.5 (9)
S3—Cu22—Cu33	83.28 (13)	Cu13—Cu62B—S22	59.6 (3)
S15^iv^—Cu22—Cu33	129.43 (15)	Cu52—Cu62B—S22	53.8 (3)
S2—Cu22—Cu33	54.60 (12)	Cu33—Cu62B—S22	129.1 (6)
S3—Cu22—Cu26	56.87 (13)	S2—Cu62B—S17	122.0 (7)
S15^iv^—Cu22—Cu26	111.13 (15)	Cu13—Cu62B—S17	53.1 (4)
S2—Cu22—Cu26	107.62 (14)	Cu52—Cu62B—S17	75.3 (4)
Cu33—Cu22—Cu26	119.44 (10)	Cu33—Cu62B—S17	104.2 (7)
S3—Cu22—Cu9^iv^	109.23 (14)	S22—Cu62B—S17	104.6 (5)
S15^iv^—Cu22—Cu9^iv^	54.04 (13)	S2—Cu62B—Cu3	56.0 (3)
S2—Cu22—Cu9^iv^	106.66 (14)	Cu13—Cu62B—Cu3	82.0 (4)
Cu33—Cu22—Cu9^iv^	161.22 (11)	Cu52—Cu62B—Cu3	150.7 (6)
Cu26—Cu22—Cu9^iv^	61.45 (8)	Cu33—Cu62B—Cu3	113.9 (4)
S3—Cu22—Cu1	54.49 (13)	S22—Cu62B—Cu3	102.2 (6)
S15^iv^—Cu22—Cu1	99.50 (15)	S17—Cu62B—Cu3	98.4 (5)
S2—Cu22—Cu1	117.27 (14)	S2—Cu62B—Cu11^i^	111.3 (6)
Cu33—Cu22—Cu1	63.49 (8)	Cu13—Cu62B—Cu11^i^	92.8 (6)
Cu26—Cu22—Cu1	110.13 (10)	Cu52—Cu62B—Cu11^i^	61.3 (4)
Cu9^iv^—Cu22—Cu1	135.14 (11)	Cu33—Cu62B—Cu11^i^	62.6 (4)
S3—Cu22—Cu25	110.28 (14)	S22—Cu62B—Cu11^i^	114.4 (5)
S15^iv^—Cu22—Cu25	102.36 (14)	S17—Cu62B—Cu11^i^	47.1 (3)
S2—Cu22—Cu25	53.95 (13)	Cu3—Cu62B—Cu11^i^	133.9 (6)
Cu33—Cu22—Cu25	101.72 (10)	S2—Cu62B—Cu32	78.0 (4)
Cu26—Cu22—Cu25	61.45 (8)	Cu13—Cu62B—Cu32	74.0 (4)
Cu9^iv^—Cu22—Cu25	61.25 (8)	Cu52—Cu62B—Cu32	123.0 (7)
Cu1—Cu22—Cu25	158.14 (11)	Cu33—Cu62B—Cu32	98.2 (5)
S14^iv^—Cu23—S4	124.53 (18)	S22—Cu62B—Cu32	132.0 (5)
S14^iv^—Cu23—S3	119.27 (17)	S17—Cu62B—Cu32	47.7 (3)
S4—Cu23—S3	112.70 (16)	Cu3—Cu62B—Cu32	57.3 (3)
S14^iv^—Cu23—Cu2	107.77 (14)	Cu11^i^—Cu62B—Cu32	77.2 (5)
S4—Cu23—Cu2	53.46 (13)	Cu31^xi^—S1—Cu32^v^	110.08 (19)
S3—Cu23—Cu2	122.23 (14)	Cu31^xi^—S1—Cu37	118.2 (2)
S14^iv^—Cu23—Cu8^iv^	53.22 (12)	Cu32^v^—S1—Cu37	114.01 (18)
S4—Cu23—Cu8^iv^	99.43 (14)	Cu31^xi^—S1—Cu34	129.0 (2)
S3—Cu23—Cu8^iv^	101.81 (14)	Cu32^v^—S1—Cu34	71.87 (14)
Cu2—Cu23—Cu8^iv^	133.87 (11)	Cu37—S1—Cu34	104.82 (17)
S14^iv^—Cu23—Cu1	100.67 (14)	Cu31^xi^—S1—Cu3^v^	72.52 (14)
S4—Cu23—Cu1	126.87 (14)	Cu32^v^—S1—Cu3^v^	72.21 (14)
S3—Cu23—Cu1	51.92 (13)	Cu37—S1—Cu3^v^	82.12 (16)
Cu2—Cu23—Cu1	89.69 (10)	Cu34—S1—Cu3^v^	143.0 (2)
Cu8^iv^—Cu23—Cu1	131.78 (11)	Cu31^xi^—S1—Cu24	72.16 (13)
S13^iv^—Cu24—S4	121.13 (17)	Cu32^v^—S1—Cu24	126.6 (2)
S13^iv^—Cu24—S1	132.55 (17)	Cu37—S1—Cu24	109.65 (19)
S4—Cu24—S1	105.23 (16)	Cu34—S1—Cu24	68.50 (13)
S13^iv^—Cu24—Cu34	138.01 (15)	Cu3^v^—S1—Cu24	144.15 (19)
S4—Cu24—Cu34	80.08 (13)	Cu62B—S2—Cu33	66.6 (4)
S1—Cu24—Cu34	55.21 (12)	Cu62A—S2—Cu33	77.59 (16)
S13^iv^—Cu24—Cu7^iv^	54.20 (12)	Cu62B—S2—Cu25	113.6 (5)
S4—Cu24—Cu7^iv^	107.17 (14)	Cu62A—S2—Cu25	88.5 (2)
S1—Cu24—Cu7^iv^	105.00 (13)	Cu33—S2—Cu25	129.3 (2)
Cu34—Cu24—Cu7^iv^	160.19 (11)	Cu62B—S2—Cu21	115.8 (5)
S13^iv^—Cu24—Cu31^xi^	82.47 (13)	Cu62A—S2—Cu21	135.4 (2)
S4—Cu24—Cu31^xi^	143.24 (15)	Cu33—S2—Cu21	111.61 (19)
S1—Cu24—Cu31^xi^	52.26 (11)	Cu25—S2—Cu21	112.19 (17)
Cu34—Cu24—Cu31^xi^	101.24 (9)	Cu62B—S2—Cu22	125.6 (4)
Cu7^iv^—Cu24—Cu31^xi^	61.72 (8)	Cu62A—S2—Cu22	114.1 (2)
S13^iv^—Cu24—Cu21^ii^	54.26 (12)	Cu33—S2—Cu22	70.43 (14)
S4—Cu24—Cu21^ii^	105.98 (14)	Cu25—S2—Cu22	71.74 (14)
S1—Cu24—Cu21^ii^	124.05 (14)	Cu21—S2—Cu22	109.91 (17)
Cu34—Cu24—Cu21^ii^	86.44 (9)	Cu62B—S2—Cu3	73.0 (4)
Cu7^iv^—Cu24—Cu21^ii^	108.42 (9)	Cu62A—S2—Cu3	71.80 (15)
Cu31^xi^—Cu24—Cu21^ii^	110.77 (10)	Cu33—S2—Cu3	137.20 (19)
S13^iv^—Cu24—Cu2	102.12 (13)	Cu25—S2—Cu3	79.48 (15)
S4—Cu24—Cu2	52.05 (12)	Cu21—S2—Cu3	73.87 (14)
S1—Cu24—Cu2	114.73 (13)	Cu22—S2—Cu3	150.2 (2)
Cu34—Cu24—Cu2	60.45 (8)	Cu22—S3—Cu23	114.86 (18)
Cu7^iv^—Cu24—Cu2	138.51 (10)	Cu22—S3—Cu44	109.39 (19)
Cu31^xi^—Cu24—Cu2	157.31 (11)	Cu23—S3—Cu44	115.35 (19)
Cu21^ii^—Cu24—Cu2	58.18 (8)	Cu22—S3—Cu35	124.6 (2)
S12^iv^—Cu25—S2	122.41 (17)	Cu23—S3—Cu35	113.01 (19)
S12^iv^—Cu25—S6	121.90 (18)	Cu44—S3—Cu35	71.87 (14)
S2—Cu25—S6	115.35 (16)	Cu22—S3—Cu1	73.12 (15)
S12^iv^—Cu25—Cu30^iv^	55.42 (12)	Cu23—S3—Cu1	77.46 (15)
S2—Cu25—Cu30^iv^	103.88 (14)	Cu44—S3—Cu1	73.07 (15)
S6—Cu25—Cu30^iv^	116.64 (15)	Cu35—S3—Cu1	144.5 (2)
S12^iv^—Cu25—Cu22	106.59 (14)	Cu22—S3—Cu26	71.06 (14)
S2—Cu25—Cu22	54.31 (12)	Cu23—S3—Cu26	105.73 (18)
S6—Cu25—Cu22	101.26 (14)	Cu44—S3—Cu26	132.4 (2)
Cu30^iv^—Cu25—Cu22	142.05 (11)	Cu35—S3—Cu26	70.65 (14)
S12^iv^—Cu25—Cu9^iv^	53.48 (12)	Cu1—S3—Cu26	141.63 (19)
S2—Cu25—Cu9^iv^	104.92 (13)	Cu45B—S4—Cu23	95.3 (11)
S6—Cu25—Cu9^iv^	107.08 (13)	Cu23—S4—Cu45A	108.8 (2)
Cu30^iv^—Cu25—Cu9^iv^	108.23 (10)	Cu45B—S4—Cu2	77.8 (10)
Cu22—Cu25—Cu9^iv^	58.89 (8)	Cu23—S4—Cu2	74.66 (16)
S12^iv^—Cu25—Cu26	103.82 (14)	Cu45A—S4—Cu2	72.15 (18)
S2—Cu25—Cu26	105.59 (14)	Cu45B—S4—Cu24	134.8 (13)
S6—Cu25—Cu26	52.44 (12)	Cu23—S4—Cu24	111.95 (18)
Cu30^iv^—Cu25—Cu26	150.23 (12)	Cu45A—S4—Cu24	117.9 (3)
Cu22—Cu25—Cu26	58.78 (8)	Cu2—S4—Cu24	75.85 (15)
Cu9^iv^—Cu25—Cu26	59.76 (8)	Cu23—S4—Cu36A	125.1 (2)
S12^iv^—Cu25—Cu5	100.60 (14)	Cu45A—S4—Cu36A	66.6 (2)
S2—Cu25—Cu5	121.04 (13)	Cu2—S4—Cu36A	138.2 (2)
S6—Cu25—Cu5	50.03 (12)	Cu24—S4—Cu36A	117.8 (2)
Cu30^iv^—Cu25—Cu5	67.54 (9)	Cu45B—S4—Cu27	120.5 (11)
Cu22—Cu25—Cu5	148.73 (10)	Cu23—S4—Cu27	87.74 (17)
Cu9^iv^—Cu25—Cu5	133.74 (10)	Cu45A—S4—Cu27	129.9 (2)
Cu26—Cu25—Cu5	100.12 (10)	Cu2—S4—Cu27	156.2 (2)
S12^iv^—Cu25—Cu3	106.21 (13)	Cu24—S4—Cu27	96.71 (17)
S2—Cu25—Cu3	50.42 (11)	Cu36A—S4—Cu27	65.39 (18)
S6—Cu25—Cu3	114.70 (13)	Cu46—S5—Cu4^iii^	103.85 (19)
Cu30^iv^—Cu25—Cu3	59.04 (8)	Cu46—S5—Cu30^iv^	148.9 (2)
Cu22—Cu25—Cu3	104.39 (9)	Cu4^iii^—S5—Cu30^iv^	107.14 (19)
Cu9^iv^—Cu25—Cu3	137.57 (10)	Cu46—S5—Cu5	75.15 (16)
Cu26—Cu25—Cu3	148.94 (10)	Cu4^iii^—S5—Cu5	122.3 (2)
Cu5—Cu25—Cu3	82.05 (8)	Cu30^iv^—S5—Cu5	85.86 (17)
S11^iv^—Cu26—S6	128.71 (18)	Cu46—S5—Cu3	91.47 (18)
S11^iv^—Cu26—S3	114.98 (17)	Cu4^iii^—S5—Cu3	120.9 (2)
S6—Cu26—S3	115.92 (17)	Cu30^iv^—S5—Cu3	75.20 (15)
S11^iv^—Cu26—Cu22	102.69 (14)	Cu5—S5—Cu3	116.9 (2)
S6—Cu26—Cu22	103.10 (14)	Cu46—S5—Cu28	114.32 (19)
S3—Cu26—Cu22	52.07 (12)	Cu4^iii^—S5—Cu28	63.04 (13)
S11^iv^—Cu26—Cu35	139.20 (15)	Cu30^iv^—S5—Cu28	78.13 (15)
S6—Cu26—Cu35	79.92 (13)	Cu5—S5—Cu28	65.56 (13)
S3—Cu26—Cu35	53.16 (11)	Cu3—S5—Cu28	152.86 (19)
Cu22—Cu26—Cu35	96.29 (10)	Cu29B—S6—Cu47B	136.1 (8)
S11^iv^—Cu26—Cu6	108.80 (15)	Cu29B—S6—Cu5	71.8 (5)
S6—Cu26—Cu6	54.87 (13)	Cu47B—S6—Cu5	84.3 (4)
S3—Cu26—Cu6	113.10 (13)	Cu47A—S6—Cu5	98.6 (4)
Cu22—Cu26—Cu6	148.46 (11)	Cu29B—S6—Cu26	89.7 (6)
Cu35—Cu26—Cu6	60.43 (8)	Cu47B—S6—Cu26	126.8 (4)
S11^iv^—Cu26—Cu9^iv^	52.90 (12)	Cu47A—S6—Cu26	114.5 (4)
S6—Cu26—Cu9^iv^	108.40 (14)	Cu5—S6—Cu26	145.5 (2)
S3—Cu26—Cu9^iv^	103.32 (13)	Cu29B—S6—Cu25	93.8 (7)
Cu22—Cu26—Cu9^iv^	59.29 (8)	Cu47B—S6—Cu25	117.3 (5)
Cu35—Cu26—Cu9^iv^	155.15 (12)	Cu47A—S6—Cu25	127.0 (4)
Cu6—Cu26—Cu9^iv^	143.58 (12)	Cu5—S6—Cu25	78.48 (15)
S11^iv^—Cu26—Cu25	106.92 (14)	Cu26—S6—Cu25	73.88 (14)
S6—Cu26—Cu25	53.68 (12)	Cu47A—S6—Cu29A	119.3 (4)
S3—Cu26—Cu25	104.69 (14)	Cu5—S6—Cu29A	72.40 (16)
Cu22—Cu26—Cu25	59.77 (8)	Cu26—S6—Cu29A	98.09 (19)
Cu35—Cu26—Cu25	113.84 (10)	Cu25—S6—Cu29A	110.2 (2)
Cu6—Cu26—Cu25	107.87 (10)	Cu29B—S6—Cu6	76.0 (6)
Cu9^iv^—Cu26—Cu25	59.98 (8)	Cu47B—S6—Cu6	91.5 (4)
S11^iv^—Cu26—Cu29A^iv^	59.08 (15)	Cu47A—S6—Cu6	77.4 (4)
S6—Cu26—Cu29A^iv^	109.14 (18)	Cu5—S6—Cu6	127.2 (2)
S3—Cu26—Cu29A^iv^	108.20 (15)	Cu26—S6—Cu6	72.43 (15)
Cu22—Cu26—Cu29A^iv^	147.56 (15)	Cu25—S6—Cu6	144.7 (2)
Cu35—Cu26—Cu29A^iv^	86.05 (11)	Cu29A—S6—Cu6	65.15 (17)
Cu6—Cu26—Cu29A^iv^	57.94 (13)	Cu29B^iv^—S7—Cu35	127.4 (6)
Cu9^iv^—Cu26—Cu29A^iv^	111.81 (13)	Cu29A^iv^—S7—Cu35	113.7 (2)
Cu25—Cu26—Cu29A^iv^	147.10 (13)	Cu29B^iv^—S7—Cu6	70.4 (5)
S10^iv^—Cu27—S7	135.41 (18)	Cu29A^iv^—S7—Cu6	73.36 (16)
S10^iv^—Cu27—S4	113.34 (18)	Cu35—S7—Cu6	74.43 (15)
S7—Cu27—S4	101.50 (16)	Cu29B^iv^—S7—Cu38	91.7 (7)
S10^iv^—Cu27—Cu29A^iv^	81.60 (16)	Cu29A^iv^—S7—Cu38	109.8 (2)
S7—Cu27—Cu29A^iv^	53.93 (14)	Cu35—S7—Cu38	115.68 (18)
S4—Cu27—Cu29A^iv^	133.8 (2)	Cu6—S7—Cu38	75.45 (15)
S10^iv^—Cu27—S8	112.16 (18)	Cu29B^iv^—S7—Cu36B	128.8 (7)
S7—Cu27—S8	91.42 (15)	Cu35—S7—Cu36B	99.5 (6)
S4—Cu27—S8	93.90 (16)	Cu6—S7—Cu36B	152.7 (6)
Cu29A^iv^—Cu27—S8	121.7 (2)	Cu38—S7—Cu36B	83.9 (7)
S10^iv^—Cu27—Cu36A	158.4 (2)	Cu29B^iv^—S7—Cu27	72.3 (5)
S7—Cu27—Cu36A	65.63 (18)	Cu29A^iv^—S7—Cu27	65.96 (15)
S4—Cu27—Cu36A	56.3 (2)	Cu35—S7—Cu27	118.08 (19)
Cu29A^iv^—Cu27—Cu36A	119.56 (19)	Cu6—S7—Cu27	139.09 (19)
S8—Cu27—Cu36A	54.70 (15)	Cu38—S7—Cu27	122.0 (2)
S10^iv^—Cu27—Cu36B	161.9 (5)	Cu36B—S7—Cu27	67.5 (5)
S7—Cu27—Cu36B	55.4 (6)	Cu29A^iv^—S7—Cu36A	126.5 (2)
S4—Cu27—Cu36B	71.4 (7)	Cu35—S7—Cu36A	92.47 (18)
S8—Cu27—Cu36B	49.8 (5)	Cu6—S7—Cu36A	159.9 (2)
S10^iv^—Cu27—Cu29B^iv^	87.4 (4)	Cu38—S7—Cu36A	97.5 (2)
S7—Cu27—Cu29B^iv^	48.8 (4)	Cu27—S7—Cu36A	60.59 (14)
S4—Cu27—Cu29B^iv^	143.0 (4)	Cu36B—S8—Cu37	91.2 (7)
Cu29A^iv^—Cu27—Cu29B^iv^	15.2 (4)	Cu28^iv^—S8—Cu37	107.4 (2)
S8—Cu27—Cu29B^iv^	106.5 (5)	Cu36B—S8—Cu39	81.7 (8)
Cu36A—Cu27—Cu29B^iv^	112.0 (4)	Cu28^iv^—S8—Cu39	122.4 (2)
S10^iv^—Cu27—Cu28^iv^	63.40 (14)	Cu37—S8—Cu39	112.90 (19)
S7—Cu27—Cu28^iv^	128.21 (15)	Cu36B—S8—Cu4	145.7 (6)
S4—Cu27—Cu28^iv^	111.34 (15)	Cu28^iv^—S8—Cu4	77.33 (16)
Cu29A^iv^—Cu27—Cu28^iv^	114.29 (15)	Cu37—S8—Cu4	73.37 (16)
S8—Cu27—Cu28^iv^	48.80 (12)	Cu39—S8—Cu4	76.82 (16)
Cu36A—Cu27—Cu28^iv^	100.95 (13)	Cu28^iv^—S8—Cu36A	128.9 (2)
S10^iv^—Cu27—Cu8^iv^	49.02 (13)	Cu37—S8—Cu36A	79.9 (2)
S7—Cu27—Cu8^iv^	103.63 (14)	Cu39—S8—Cu36A	97.8 (2)
S4—Cu27—Cu8^iv^	94.10 (14)	Cu4—S8—Cu36A	147.7 (2)
Cu29A^iv^—Cu27—Cu8^iv^	62.04 (16)	Cu36B—S8—Cu27	66.7 (5)
S8—Cu27—Cu8^iv^	161.17 (15)	Cu28^iv^—S8—Cu27	70.39 (14)
Cu36A—Cu27—Cu8^iv^	142.32 (17)	Cu37—S8—Cu27	119.6 (2)
Cu28^iv^—Cu27—Cu8^iv^	112.37 (10)	Cu39—S8—Cu27	117.7 (2)
S10^iv^—Cu27—Cu5^iv^	49.47 (13)	Cu4—S8—Cu27	147.5 (2)
S7—Cu27—Cu5^iv^	97.46 (14)	Cu36A—S8—Cu27	63.02 (15)
S4—Cu27—Cu5^iv^	160.96 (15)	Cu48^v^—S9—Cu4	84.23 (16)
Cu29A^iv^—Cu27—Cu5^iv^	58.61 (11)	Cu48^v^—S9—Cu7^v^	78.62 (15)
S8—Cu27—Cu5^iv^	87.28 (13)	Cu4—S9—Cu7^v^	124.1 (2)
Cu36A—Cu27—Cu5^iv^	135.8 (2)	Cu48^v^—S9—Cu28^v^	115.99 (19)
Cu28^iv^—Cu27—Cu5^iv^	56.27 (9)	Cu4—S9—Cu28^v^	69.73 (15)
Cu8^iv^—Cu27—Cu5^iv^	79.69 (9)	Cu7^v^—S9—Cu28^v^	71.26 (15)
S8^iv^—Cu28—S9^iii^	132.68 (19)	Cu48^v^—S9—Cu31	119.01 (19)
S8^iv^—Cu28—Cu4^iii^	131.44 (16)	Cu4—S9—Cu31	154.8 (2)
S9^iii^—Cu28—Cu4^iii^	55.05 (13)	Cu7^v^—S9—Cu31	73.50 (15)
S8^iv^—Cu28—Cu7	122.55 (17)	Cu28^v^—S9—Cu31	104.55 (17)
S9^iii^—Cu28—Cu7	54.32 (13)	Cu48^v^—S9—Cu30	120.86 (19)
Cu4^iii^—Cu28—Cu7	99.78 (10)	Cu4—S9—Cu30	81.60 (16)
S8^iv^—Cu28—S10	109.55 (17)	Cu7^v^—S9—Cu30	151.0 (2)
S9^iii^—Cu28—S10	98.95 (16)	Cu28^v^—S9—Cu30	111.87 (18)
Cu4^iii^—Cu28—S10	116.13 (13)	Cu31—S9—Cu30	78.00 (15)
Cu7—Cu28—S10	49.82 (11)	Cu8—S10—Cu27^iv^	80.41 (16)
S8^iv^—Cu28—S5	115.50 (18)	Cu8—S10—Cu7	121.4 (2)
S9^iii^—Cu28—S5	100.13 (16)	Cu27^iv^—S10—Cu7	101.00 (18)
Cu4^iii^—Cu28—S5	49.83 (11)	Cu8—S10—Cu49	92.03 (17)
Cu7—Cu28—S5	117.55 (13)	Cu27^iv^—S10—Cu49	152.7 (2)
S10—Cu28—S5	91.61 (15)	Cu7—S10—Cu49	105.31 (18)
S8^iv^—Cu28—Cu5	101.72 (15)	Cu8—S10—Cu5	113.4 (2)
S9^iii^—Cu28—Cu5	125.42 (14)	Cu27^iv^—S10—Cu5	81.98 (16)
Cu4^iii^—Cu28—Cu5	95.28 (11)	Cu7—S10—Cu5	124.9 (2)
Cu7—Cu28—Cu5	96.56 (10)	Cu49—S10—Cu5	77.00 (16)
S10—Cu28—Cu5	50.09 (11)	Cu8—S10—Cu28	148.14 (19)
S5—Cu28—Cu5	49.15 (11)	Cu27^iv^—S10—Cu28	67.84 (14)
S8^iv^—Cu28—Cu27^iv^	60.81 (13)	Cu7—S10—Cu28	65.00 (13)
S9^iii^—Cu28—Cu27^iv^	132.01 (16)	Cu49—S10—Cu28	117.36 (19)
Cu4^iii^—Cu28—Cu27^iv^	160.43 (13)	Cu5—S10—Cu28	65.84 (14)
Cu7—Cu28—Cu27^iv^	79.30 (10)	Cu8—S11—Cu50	102.93 (19)
S10—Cu28—Cu27^iv^	48.76 (11)	Cu8—S11—Cu26^iv^	102.45 (18)
S5—Cu28—Cu27^iv^	113.06 (14)	Cu50—S11—Cu26^iv^	154.4 (2)
Cu5—Cu28—Cu27^iv^	65.62 (9)	Cu8—S11—Cu9	122.5 (2)
S8^iv^—Cu28—Cu4^iv^	52.12 (13)	Cu50—S11—Cu9	94.58 (17)
S9^iii^—Cu28—Cu4^iv^	98.24 (14)	Cu26^iv^—S11—Cu9	74.44 (15)
Cu4^iii^—Cu28—Cu4^iv^	80.50 (10)	Cu8—S11—Cu6	123.2 (2)
Cu7—Cu28—Cu4^iv^	140.30 (12)	Cu50—S11—Cu6	72.35 (15)
S10—Cu28—Cu4^iv^	161.06 (13)	Cu26^iv^—S11—Cu6	90.85 (17)
S5—Cu28—Cu4^iv^	93.13 (13)	Cu9—S11—Cu6	114.2 (2)
Cu5—Cu28—Cu4^iv^	123.06 (12)	Cu8—S11—Cu29A	70.83 (16)
Cu27^iv^—Cu28—Cu4^iv^	112.84 (10)	Cu50—S11—Cu29A	114.7 (2)
S7^iv^—Cu29A—S6	128.5 (3)	Cu26^iv^—S11—Cu29A	70.99 (14)
S7^iv^—Cu29A—Cu6	136.2 (2)	Cu9—S11—Cu29A	145.1 (2)
S6—Cu29A—Cu6	57.86 (14)	Cu6—S11—Cu29A	62.03 (15)
S7^iv^—Cu29A—Cu27^iv^	60.11 (14)	Cu8—S11—Cu42	62.09 (14)
S6—Cu29A—Cu27^iv^	121.1 (2)	Cu50—S11—Cu42	80.75 (15)
Cu6—Cu29A—Cu27^iv^	162.3 (2)	Cu26^iv^—S11—Cu42	114.57 (19)
S7^iv^—Cu29A—S11	126.1 (3)	Cu9—S11—Cu42	67.70 (14)
S6—Cu29A—S11	101.92 (19)	Cu6—S11—Cu42	153.09 (19)
Cu6—Cu29A—S11	54.56 (15)	Cu29A—S11—Cu42	132.8 (2)
Cu27^iv^—Cu29A—S11	112.4 (3)	Cu43—S12—Cu38	116.24 (18)
S7^iv^—Cu29A—Cu6^iv^	53.92 (14)	Cu43—S12—Cu30	109.21 (19)
S6—Cu29A—Cu6^iv^	102.8 (2)	Cu38—S12—Cu30	126.5 (2)
Cu6—Cu29A—Cu6^iv^	82.35 (12)	Cu43—S12—Cu25^iv^	113.63 (19)
Cu27^iv^—Cu29A—Cu6^iv^	113.90 (14)	Cu38—S12—Cu25^iv^	113.27 (19)
S11—Cu29A—Cu6^iv^	102.48 (17)	Cu30—S12—Cu25^iv^	69.08 (13)
S7^iv^—Cu29A—Cu5	109.5 (2)	Cu43—S12—Cu9	76.53 (15)
S6—Cu29A—Cu5	52.91 (14)	Cu38—S12—Cu9	77.76 (15)
Cu6—Cu29A—Cu5	104.53 (13)	Cu30—S12—Cu9	141.5 (2)
Cu27^iv^—Cu29A—Cu5	69.00 (12)	Cu25^iv^—S12—Cu9	73.80 (14)
S11—Cu29A—Cu5	116.37 (18)	Cu43—S12—Cu39	102.51 (18)
Cu6^iv^—Cu29A—Cu5	136.8 (3)	Cu38—S12—Cu39	73.63 (15)
S7^iv^—Cu29A—Cu8	110.8 (2)	Cu30—S12—Cu39	70.27 (15)
S6—Cu29A—Cu8	114.47 (18)	Cu25^iv^—S12—Cu39	132.3 (2)
Cu6—Cu29A—Cu8	98.5 (2)	Cu9—S12—Cu39	147.4 (2)
Cu27^iv^—Cu29A—Cu8	65.14 (14)	Cu41—S13—Cu24^iv^	115.25 (19)
S11—Cu29A—Cu8	49.71 (16)	Cu41—S13—Cu40	113.18 (18)
Cu6^iv^—Cu29A—Cu8	136.4 (2)	Cu24^iv^—S13—Cu40	108.87 (18)
Cu5—Cu29A—Cu8	85.57 (13)	Cu41—S13—Cu7	78.89 (15)
S7^iv^—Cu29A—Cu26^iv^	79.21 (15)	Cu24^iv^—S13—Cu7	72.81 (14)
S6—Cu29A—Cu26^iv^	132.71 (18)	Cu40—S13—Cu7	69.15 (14)
Cu6—Cu29A—Cu26^iv^	75.64 (14)	Cu41—S13—Cu21^vi^	113.72 (19)
Cu27^iv^—Cu29A—Cu26^iv^	105.76 (18)	Cu24^iv^—S13—Cu21^vi^	73.32 (13)
S11—Cu29A—Cu26^iv^	49.93 (15)	Cu40—S13—Cu21^vi^	125.7 (2)
Cu6^iv^—Cu29A—Cu26^iv^	59.71 (10)	Cu7—S13—Cu21^vi^	146.02 (19)
Cu5—Cu29A—Cu26^iv^	163.5 (3)	Cu41—S13—Cu32^vi^	105.88 (16)
Cu8—Cu29A—Cu26^iv^	78.14 (18)	Cu24^iv^—S13—Cu32^vi^	133.32 (19)
S6—Cu29B—S7^iv^	163.8 (14)	Cu40—S13—Cu32^vi^	71.65 (14)
S6—Cu29B—Cu6^iv^	118.2 (8)	Cu7—S13—Cu32^vi^	138.81 (19)
S7^iv^—Cu29B—Cu6^iv^	57.9 (4)	Cu21^vi^—S13—Cu32^vi^	70.55 (13)
S6—Cu29B—Cu5	58.3 (4)	Cu23^iv^—S14—Cu42	118.75 (19)
S7^iv^—Cu29B—Cu5	121.3 (7)	Cu23^iv^—S14—Cu1^vi^	96.20 (17)
Cu6^iv^—Cu29B—Cu5	166.6 (12)	Cu42—S14—Cu1^vi^	71.88 (15)
S6—Cu29B—Cu27^iv^	127.0 (7)	Cu23^iv^—S14—Cu41	113.42 (19)
S7^iv^—Cu29B—Cu27^iv^	58.9 (4)	Cu42—S14—Cu41	101.19 (18)
Cu6^iv^—Cu29B—Cu27^iv^	114.7 (6)	Cu1^vi^—S14—Cu41	148.4 (2)
Cu5—Cu29B—Cu27^iv^	69.7 (4)	Cu23^iv^—S14—Cu8	76.02 (15)
S6—Cu29B—Cu6	57.1 (4)	Cu42—S14—Cu8	67.19 (15)
S7^iv^—Cu29B—Cu6	131.9 (8)	Cu1^vi^—S14—Cu8	126.8 (2)
Cu6^iv^—Cu29B—Cu6	81.7 (5)	Cu41—S14—Cu8	73.65 (15)
Cu5—Cu29B—Cu6	104.1 (5)	Cu23^iv^—S14—Cu33^vi^	132.1 (2)
Cu27^iv^—Cu29B—Cu6	135.6 (10)	Cu42—S14—Cu33^vi^	103.51 (17)
S5^iv^—Cu30—S12	138.15 (18)	Cu1^vi^—S14—Cu33^vi^	75.50 (15)
S5^iv^—Cu30—S9	109.47 (17)	Cu41—S14—Cu33^vi^	76.40 (14)
S12—Cu30—S9	112.08 (16)	Cu8—S14—Cu33^vi^	145.8 (2)
S5^iv^—Cu30—Cu25^iv^	85.27 (14)	Cu51A—S15—Cu22^iv^	152.3 (3)
S12—Cu30—Cu25^iv^	55.49 (12)	Cu51A—S15—Cu2^vi^	87.5 (3)
S9—Cu30—Cu25^iv^	150.43 (16)	Cu22^iv^—S15—Cu2^vi^	107.67 (19)
S5^iv^—Cu30—Cu39	141.59 (17)	Cu22^iv^—S15—Cu51B	154.3 (4)
S12—Cu30—Cu39	55.33 (12)	Cu2^vi^—S15—Cu51B	96.9 (4)
S9—Cu30—Cu39	77.89 (13)	Cu51A—S15—Cu1^vi^	96.3 (3)
Cu25^iv^—Cu30—Cu39	106.81 (11)	Cu22^iv^—S15—Cu1^vi^	94.68 (18)
S5^iv^—Cu30—Cu3^iv^	52.79 (13)	Cu2^vi^—S15—Cu1^vi^	122.4 (2)
S12—Cu30—Cu3^iv^	113.38 (15)	Cu51A—S15—Cu9	79.4 (3)
S9—Cu30—Cu3^iv^	100.65 (14)	Cu22^iv^—S15—Cu9	72.94 (15)
Cu25^iv^—Cu30—Cu3^iv^	67.32 (9)	Cu2^vi^—S15—Cu9	121.3 (2)
Cu39—Cu30—Cu3^iv^	165.38 (12)	Cu51B—S15—Cu9	88.0 (3)
S5^iv^—Cu30—Cu31	97.01 (14)	Cu1^vi^—S15—Cu9	115.8 (2)
S12—Cu30—Cu31	105.11 (15)	Cu21^iv^—S16—Cu43	117.66 (19)
S9—Cu30—Cu31	50.85 (12)	Cu21^iv^—S16—Cu34^vi^	107.75 (18)
Cu25^iv^—Cu30—Cu31	103.28 (10)	Cu43—S16—Cu34^vi^	113.25 (17)
Cu39—Cu30—Cu31	114.52 (10)	Cu21^iv^—S16—Cu31	69.59 (13)
Cu3^iv^—Cu30—Cu31	56.61 (8)	Cu43—S16—Cu31	116.90 (19)
S5^iv^—Cu30—Cu4	97.18 (14)	Cu34^vi^—S16—Cu31	123.6 (2)
S12—Cu30—Cu4	113.94 (14)	Cu21^iv^—S16—Cu2^vi^	72.30 (14)
S9—Cu30—Cu4	48.29 (12)	Cu43—S16—Cu2^vi^	76.84 (15)
Cu25^iv^—Cu30—Cu4	158.11 (12)	Cu34^vi^—S16—Cu2^vi^	73.45 (14)
Cu39—Cu30—Cu4	58.71 (8)	Cu31—S16—Cu2^vi^	141.52 (19)
Cu3^iv^—Cu30—Cu4	130.73 (10)	Cu21^iv^—S16—Cu40^v^	128.50 (19)
Cu31—Cu30—Cu4	98.02 (9)	Cu43—S16—Cu40^v^	108.87 (18)
S1^xi^—Cu31—S16	134.34 (18)	Cu34^vi^—S16—Cu40^v^	70.20 (14)
S1^xi^—Cu31—S9	118.24 (17)	Cu31—S16—Cu40^v^	70.62 (14)
S16—Cu31—S9	105.93 (16)	Cu2^vi^—S16—Cu40^v^	142.32 (19)
S1^xi^—Cu31—Cu21^iv^	82.06 (13)	Cu13—S17—Cu11^i^	119.6 (2)
S16—Cu31—Cu21^iv^	54.13 (12)	Cu13—S17—Cu56^xiii^	92.73 (18)
S9—Cu31—Cu21^iv^	143.89 (15)	Cu11^i^—S17—Cu56^xiii^	98.03 (17)
S1^xi^—Cu31—Cu40^v^	139.27 (15)	Cu13—S17—Cu32	92.01 (18)
S16—Cu31—Cu40^v^	54.98 (11)	Cu11^i^—S17—Cu32	109.41 (19)
S9—Cu31—Cu40^v^	81.55 (12)	Cu56^xiii^—S17—Cu32	145.1 (2)
Cu21^iv^—Cu31—Cu40^v^	102.58 (10)	Cu13—S17—Cu10^xiv^	115.3 (2)
S1^xi^—Cu31—Cu24^xi^	55.58 (12)	Cu11^i^—S17—Cu10^xiv^	124.9 (2)
S16—Cu31—Cu24^xi^	125.13 (14)	Cu56^xiii^—S17—Cu10^xiv^	74.27 (15)
S9—Cu31—Cu24^xi^	104.28 (13)	Cu32—S17—Cu10^xiv^	72.52 (14)
Cu21^iv^—Cu31—Cu24^xi^	111.76 (10)	Cu13—S17—Cu62B	57.8 (3)
Cu40^v^—Cu31—Cu24^xi^	86.20 (9)	Cu11^i^—S17—Cu62B	74.2 (4)
S1^xi^—Cu31—Cu3^iv^	55.29 (12)	Cu56^xiii^—S17—Cu62B	135.4 (3)
S16—Cu31—Cu3^iv^	106.25 (13)	Cu32—S17—Cu62B	74.5 (3)
S9—Cu31—Cu3^iv^	102.22 (13)	Cu10^xiv^—S17—Cu62B	146.0 (3)
Cu21^iv^—Cu31—Cu3^iv^	63.28 (8)	Cu33—S18—Cu59^i^	115.9 (2)
Cu40^v^—Cu31—Cu3^iv^	160.80 (11)	Cu33—S18—Cu52	107.9 (2)
Cu24^xi^—Cu31—Cu3^iv^	110.63 (9)	Cu59^i^—S18—Cu52	115.85 (19)
S1^xi^—Cu31—Cu7^v^	105.42 (14)	Cu33—S18—Cu11^i^	76.35 (15)
S16—Cu31—Cu7^v^	110.46 (13)	Cu59^i^—S18—Cu11^i^	73.49 (16)
S9—Cu31—Cu7^v^	52.38 (12)	Cu52—S18—Cu11^i^	74.17 (15)
Cu21^iv^—Cu31—Cu7^v^	155.80 (11)	Cu33—S18—Cu44	73.59 (14)
Cu40^v^—Cu31—Cu7^v^	56.65 (7)	Cu59^i^—S18—Cu44	110.6 (2)
Cu24^xi^—Cu31—Cu7^v^	58.97 (8)	Cu52—S18—Cu44	125.6 (2)
Cu3^iv^—Cu31—Cu7^v^	139.83 (10)	Cu11^i^—S18—Cu44	148.0 (2)
S1^xi^—Cu31—Cu30	104.86 (14)	Cu33—S18—Cu53	133.8 (2)
S16—Cu31—Cu30	93.47 (13)	Cu59^i^—S18—Cu53	103.40 (18)
S9—Cu31—Cu30	51.15 (12)	Cu52—S18—Cu53	72.73 (15)
Cu21^iv^—Cu31—Cu30	96.61 (9)	Cu11^i^—S18—Cu53	141.01 (19)
Cu40^v^—Cu31—Cu30	114.53 (9)	Cu44—S18—Cu53	70.39 (14)
Cu24^xi^—Cu31—Cu30	140.57 (10)	Cu12^i^—S19—Cu54A^x^	87.1 (3)
Cu3^iv^—Cu31—Cu30	58.09 (7)	Cu12^i^—S19—Cu51B^i^	74.7 (4)
Cu7^v^—Cu31—Cu30	103.38 (9)	Cu54A^x^—S19—Cu51B^i^	120.9 (3)
S1^iii^—Cu32—S17	131.38 (18)	Cu12^i^—S19—Cu45A	58.4 (3)
S1^iii^—Cu32—S13^i^	117.79 (16)	Cu54A^x^—S19—Cu45A	115.3 (3)
S17—Cu32—S13^i^	110.74 (17)	Cu45B—S19—Cu14	68.9 (9)
S1^iii^—Cu32—Cu34^iii^	55.14 (12)	Cu54B^x^—S19—Cu14	72.7 (4)
S17—Cu32—Cu34^iii^	111.93 (14)	Cu12^i^—S19—Cu14	122.1 (3)
S13^i^—Cu32—Cu34^iii^	102.88 (13)	Cu54A^x^—S19—Cu14	81.73 (19)
S1^iii^—Cu32—Cu10^xiv^	108.77 (14)	Cu51B^i^—S19—Cu14	154.2 (3)
S17—Cu32—Cu10^xiv^	54.02 (13)	Cu45A—S19—Cu14	76.0 (2)
S13^i^—Cu32—Cu10^xiv^	104.06 (13)	Cu45B—S19—Cu44	92.1 (12)
Cu34^iii^—Cu32—Cu10^xiv^	61.38 (8)	Cu54B^x^—S19—Cu44	137.8 (5)
S1^iii^—Cu32—Cu21	79.91 (12)	Cu12^i^—S19—Cu44	148.3 (3)
S17—Cu32—Cu21	135.87 (15)	Cu54A^x^—S19—Cu44	123.0 (2)
S13^i^—Cu32—Cu21	53.67 (11)	Cu51B^i^—S19—Cu44	80.6 (3)
Cu34^iii^—Cu32—Cu21	111.89 (9)	Cu45A—S19—Cu44	109.2 (3)
Cu10^xiv^—Cu32—Cu21	156.37 (11)	Cu14—S19—Cu44	76.17 (15)
S1^iii^—Cu32—Cu3	55.35 (12)	Cu12^i^—S19—Cu60B^i^	67.2 (4)
S17—Cu32—Cu3	106.52 (14)	Cu54A^x^—S19—Cu60B^i^	56.1 (3)
S13^i^—Cu32—Cu3	114.89 (13)	Cu45A—S19—Cu60B^i^	125.5 (4)
Cu34^iii^—Cu32—Cu3	109.99 (10)	Cu14—S19—Cu60B^i^	137.3 (3)
Cu10^xiv^—Cu32—Cu3	140.95 (11)	Cu44—S19—Cu60B^i^	119.0 (3)
Cu21—Cu32—Cu3	62.21 (8)	Cu12^i^—S19—Cu51A^i^	58.8 (3)
S1^iii^—Cu32—Cu40^i^	106.12 (14)	Cu54A^x^—S19—Cu51A^i^	116.7 (3)
S17—Cu32—Cu40^i^	100.42 (14)	Cu45A—S19—Cu51A^i^	91.4 (3)
S13^i^—Cu32—Cu40^i^	52.59 (11)	Cu14—S19—Cu51A^i^	161.1 (2)
Cu34^iii^—Cu32—Cu40^i^	58.77 (8)	Cu44—S19—Cu51A^i^	95.3 (2)
Cu10^xiv^—Cu32—Cu40^i^	59.11 (8)	Cu12^i^—S20—Cu55^x^	103.5 (2)
Cu21—Cu32—Cu40^i^	97.59 (9)	Cu12^i^—S20—Cu10^i^	123.5 (3)
Cu3—Cu32—Cu40^i^	153.04 (10)	Cu55^x^—S20—Cu10^i^	86.07 (18)
S1^iii^—Cu32—Cu62B	109.3 (4)	Cu12^i^—S20—Cu15	121.4 (3)
S17—Cu32—Cu62B	57.8 (3)	Cu55^x^—S20—Cu15	75.71 (16)
S13^i^—Cu32—Cu62B	105.4 (3)	Cu10^i^—S20—Cu15	115.0 (2)
Cu34^iii^—Cu32—Cu62B	151.7 (3)	Cu12^i^—S20—Cu34	98.3 (2)
Cu10^xiv^—Cu32—Cu62B	111.4 (3)	Cu55^x^—S20—Cu34	156.2 (2)
Cu21—Cu32—Cu62B	84.9 (3)	Cu10^i^—S20—Cu34	73.94 (15)
Cu3—Cu32—Cu62B	56.5 (3)	Cu15—S20—Cu34	100.93 (18)
Cu40^i^—Cu32—Cu62B	144.4 (3)	Cu12^i^—S20—Cu61^i^	57.4 (2)
S18—Cu33—S2	130.36 (18)	Cu55^x^—S20—Cu61^i^	69.19 (15)
S18—Cu33—S14^i^	120.61 (18)	Cu10^i^—S20—Cu61^i^	76.08 (17)
S2—Cu33—S14^i^	108.33 (16)	Cu15—S20—Cu61^i^	142.4 (2)
S18—Cu33—Cu62B	78.6 (3)	Cu34—S20—Cu61^i^	116.6 (2)
S2—Cu33—Cu62B	54.1 (3)	Cu12^i^—S20—Cu45A	56.6 (2)
S14^i^—Cu33—Cu62B	147.8 (4)	Cu55^x^—S20—Cu45A	121.9 (2)
S18—Cu33—Cu22	136.94 (16)	Cu10^i^—S20—Cu45A	152.0 (2)
S2—Cu33—Cu22	54.97 (12)	Cu15—S20—Cu45A	74.11 (17)
S14^i^—Cu33—Cu22	81.27 (12)	Cu34—S20—Cu45A	78.34 (16)
Cu62B—Cu33—Cu22	102.3 (4)	Cu61^i^—S20—Cu45A	113.8 (2)
S18—Cu33—Cu44	54.53 (12)	Cu16^iii^—S21—Cu13	121.2 (2)
S2—Cu33—Cu44	122.45 (15)	Cu16^iii^—S21—Cu53^xiii^	103.03 (19)
S14^i^—Cu33—Cu44	104.15 (14)	Cu13—S21—Cu53^xiii^	97.83 (19)
Cu62B—Cu33—Cu44	108.0 (4)	Cu16^iii^—S21—Cu37^iii^	87.47 (18)
Cu22—Cu33—Cu44	85.70 (9)	Cu13—S21—Cu37^iii^	104.83 (19)
S18—Cu33—Cu11^i^	52.99 (12)	Cu53^xiii^—S21—Cu37^iii^	145.3 (2)
S2—Cu33—Cu11^i^	110.31 (14)	Cu16^iii^—S21—Cu15^iii^	116.7 (2)
S14^i^—Cu33—Cu11^i^	103.00 (14)	Cu13—S21—Cu15^iii^	122.0 (2)
Cu22—Cu33—Cu11^i^	165.01 (11)	Cu53^xiii^—S21—Cu15^iii^	72.35 (15)
Cu44—Cu33—Cu11^i^	106.80 (9)	Cu37^iii^—S21—Cu15^iii^	73.34 (15)
S18—Cu33—Cu62A	81.28 (14)	Cu16^iii^—S21—Cu54A	65.78 (16)
S2—Cu33—Cu62A	49.15 (12)	Cu13—S21—Cu54A	64.53 (16)
S14^i^—Cu33—Cu62A	156.87 (14)	Cu53^xiii^—S21—Cu54A	80.44 (15)
Cu22—Cu33—Cu62A	86.99 (11)	Cu37^iii^—S21—Cu54A	133.2 (2)
Cu44—Cu33—Cu62A	94.70 (11)	Cu15^iii^—S21—Cu54A	152.6 (2)
Cu11^i^—Cu33—Cu62A	83.81 (11)	Cu54B—S22—Cu46	86.8 (5)
S18—Cu33—Cu1	104.01 (14)	Cu62A—S22—Cu46	82.0 (2)
S2—Cu33—Cu1	113.08 (13)	Cu54B—S22—Cu52	97.5 (5)
S14^i^—Cu33—Cu1	51.19 (12)	Cu62A—S22—Cu52	76.72 (19)
Cu62B—Cu33—Cu1	155.5 (5)	Cu46—S22—Cu52	147.0 (2)
Cu22—Cu33—Cu1	58.86 (8)	Cu54B—S22—Cu47B	128.5 (6)
Cu44—Cu33—Cu1	58.47 (8)	Cu46—S22—Cu47B	77.9 (4)
Cu11^i^—Cu33—Cu1	134.75 (11)	Cu52—S22—Cu47B	121.5 (4)
Cu62A—Cu33—Cu1	135.73 (13)	Cu54B—S22—Cu55	89.3 (5)
S18—Cu33—Cu41^i^	102.99 (14)	Cu62A—S22—Cu55	121.6 (2)
S2—Cu33—Cu41^i^	100.88 (14)	Cu46—S22—Cu55	129.3 (2)
S14^i^—Cu33—Cu41^i^	51.25 (12)	Cu52—S22—Cu55	83.60 (18)
Cu22—Cu33—Cu41^i^	118.47 (10)	Cu47B—S22—Cu55	65.8 (4)
Cu44—Cu33—Cu41^i^	136.25 (11)	Cu62A—S22—Cu54A	132.0 (2)
Cu11^i^—Cu33—Cu41^i^	57.77 (8)	Cu46—S22—Cu54A	66.08 (17)
Cu62A—Cu33—Cu41^i^	120.62 (12)	Cu52—S22—Cu54A	111.6 (2)
Cu1—Cu33—Cu41^i^	101.28 (9)	Cu55—S22—Cu54A	106.4 (2)
S16^i^—Cu34—S20	117.94 (17)	Cu54B—S22—Cu13	75.3 (5)
S16^i^—Cu34—S1	124.42 (16)	Cu62A—S22—Cu13	71.95 (18)
S20—Cu34—S1	117.57 (17)	Cu46—S22—Cu13	76.94 (16)
S16^i^—Cu34—Cu24	83.81 (12)	Cu52—S22—Cu13	72.61 (16)
S20—Cu34—Cu24	137.80 (15)	Cu47B—S22—Cu13	143.8 (4)
S1—Cu34—Cu24	56.29 (12)	Cu55—S22—Cu13	149.3 (2)
S16^i^—Cu34—Cu40^xii^	55.90 (12)	Cu54A—S22—Cu13	66.71 (17)
S20—Cu34—Cu40^xii^	104.92 (14)	Cu54B—S22—Cu62B	129.8 (6)
S1—Cu34—Cu40^xii^	106.54 (14)	Cu46—S22—Cu62B	91.4 (4)
Cu24—Cu34—Cu40^xii^	116.95 (10)	Cu52—S22—Cu62B	60.8 (4)
S16^i^—Cu34—Cu32^v^	110.88 (14)	Cu47B—S22—Cu62B	99.8 (6)
S20—Cu34—Cu32^v^	102.88 (14)	Cu55—S22—Cu62B	127.5 (4)
S1—Cu34—Cu32^v^	52.99 (12)	Cu13—S22—Cu62B	55.6 (4)
Cu24—Cu34—Cu32^v^	101.53 (9)	Cu62A—S22—Cu47A	80.1 (3)
Cu40^xii^—Cu34—Cu32^v^	61.35 (8)	Cu46—S22—Cu47A	87.8 (3)
S16^i^—Cu34—Cu10^i^	105.56 (13)	Cu52—S22—Cu47A	112.6 (3)
S20—Cu34—Cu10^i^	52.68 (12)	Cu55—S22—Cu47A	58.1 (3)
S1—Cu34—Cu10^i^	105.12 (13)	Cu54A—S22—Cu47A	130.2 (3)
Cu24—Cu34—Cu10^i^	160.52 (11)	Cu13—S22—Cu47A	149.6 (3)
Cu40^xii^—Cu34—Cu10^i^	59.26 (8)	Cu35—S23—Cu56	114.8 (2)
Cu32^v^—Cu34—Cu10^i^	59.32 (8)	Cu35—S23—Cu55	140.2 (2)
S16^i^—Cu34—Cu2	53.96 (12)	Cu56—S23—Cu55	92.39 (18)
S20—Cu34—Cu2	98.98 (14)	Cu35—S23—Cu53	113.2 (2)
S1—Cu34—Cu2	118.67 (13)	Cu56—S23—Cu53	112.00 (18)
Cu24—Cu34—Cu2	63.40 (8)	Cu55—S23—Cu53	78.91 (17)
Cu40^xii^—Cu34—Cu2	109.23 (10)	Cu35—S23—Cu14	75.60 (16)
Cu32^v^—Cu34—Cu2	157.81 (10)	Cu56—S23—Cu14	75.39 (15)
Cu10^i^—Cu34—Cu2	135.89 (10)	Cu55—S23—Cu14	142.3 (2)
S16^i^—Cu34—Cu45A	104.47 (15)	Cu53—S23—Cu14	73.30 (16)
S20—Cu34—Cu45A	52.53 (17)	Cu35—S23—Cu47A	75.2 (3)
S1—Cu34—Cu45A	110.63 (16)	Cu56—S23—Cu47A	116.3 (4)
Cu24—Cu34—Cu45A	88.52 (14)	Cu55—S23—Cu47A	66.5 (3)
Cu40^xii^—Cu34—Cu45A	142.48 (13)	Cu53—S23—Cu47A	120.3 (4)
Cu32^v^—Cu34—Cu45A	144.02 (13)	Cu14—S23—Cu47A	150.8 (3)
Cu10^i^—Cu34—Cu45A	105.11 (14)	Cu36B—S24—Cu17	79.3 (8)
Cu2—Cu34—Cu45A	55.93 (12)	Cu36A—S24—Cu17	97.1 (3)
S23—Cu35—S7	119.66 (18)	Cu36B—S24—Cu14	97.5 (6)
S23—Cu35—S3	130.64 (19)	Cu36A—S24—Cu14	91.6 (2)
S7—Cu35—S3	108.13 (17)	Cu17—S24—Cu14	122.1 (2)
S23—Cu35—Cu44	79.35 (14)	Cu36B—S24—Cu15	88.9 (7)
S7—Cu35—Cu44	144.05 (16)	Cu36A—S24—Cu15	76.4 (2)
S3—Cu35—Cu44	53.54 (12)	Cu17—S24—Cu15	120.0 (2)
S23—Cu35—Cu26	139.88 (16)	Cu14—S24—Cu15	117.7 (2)
S7—Cu35—Cu26	80.83 (13)	Cu36A—S24—Cu52^x^	151.6 (3)
S3—Cu35—Cu26	56.18 (12)	Cu17—S24—Cu52^x^	96.89 (18)
Cu44—Cu35—Cu26	104.39 (10)	Cu14—S24—Cu52^x^	101.64 (19)
S23—Cu35—Cu6	102.82 (15)	Cu15—S24—Cu52^x^	75.18 (15)
S7—Cu35—Cu6	52.79 (12)	Cu60A^ix^—S25—Cu16^iii^	102.3 (2)
S3—Cu35—Cu6	115.95 (14)	Cu60B^ix^—S25—Cu49	118.1 (4)
Cu44—Cu35—Cu6	158.85 (12)	Cu60A^ix^—S25—Cu49	106.9 (2)
Cu26—Cu35—Cu6	60.29 (8)	Cu16^iii^—S25—Cu49	147.7 (2)
S23—Cu35—Cu14	53.75 (13)	Cu60B^ix^—S25—Cu18	94.0 (4)
S7—Cu35—Cu14	102.01 (14)	Cu60A^ix^—S25—Cu18	75.7 (2)
S3—Cu35—Cu14	106.68 (14)	Cu16^iii^—S25—Cu18	126.5 (2)
Cu44—Cu35—Cu14	62.95 (8)	Cu49—S25—Cu18	75.04 (16)
Cu26—Cu35—Cu14	162.08 (11)	Cu60B^ix^—S25—Cu46	114.9 (4)
Cu6—Cu35—Cu14	135.20 (11)	Cu60A^ix^—S25—Cu46	129.2 (3)
S23—Cu35—Cu47A	54.9 (2)	Cu16^iii^—S25—Cu46	77.49 (16)
S7—Cu35—Cu47A	110.7 (3)	Cu49—S25—Cu46	73.43 (15)
S3—Cu35—Cu47A	119.3 (3)	Cu18—S25—Cu46	144.5 (2)
Cu44—Cu35—Cu47A	105.2 (3)	Cu60B^ix^—S25—Cu48	117.3 (4)
Cu26—Cu35—Cu47A	86.4 (2)	Cu60A^ix^—S25—Cu48	113.1 (2)
Cu6—Cu35—Cu47A	61.8 (3)	Cu16^iii^—S25—Cu48	66.57 (14)
Cu14—Cu35—Cu47A	108.7 (2)	Cu49—S25—Cu48	112.60 (18)
S24—Cu36A—S4	134.5 (3)	Cu18—S25—Cu48	65.79 (14)
S24—Cu36A—S8	120.6 (3)	Cu46—S25—Cu48	112.85 (17)
S4—Cu36A—S8	100.7 (2)	Cu60A^ix^—S25—Cu54A	71.1 (2)
S24—Cu36A—Cu45A	80.1 (2)	Cu16^iii^—S25—Cu54A	67.56 (17)
S4—Cu36A—Cu45A	54.7 (2)	Cu49—S25—Cu54A	109.3 (2)
S8—Cu36A—Cu45A	141.4 (3)	Cu18—S25—Cu54A	146.4 (2)
S24—Cu36A—Cu27	157.7 (3)	Cu46—S25—Cu54A	61.96 (14)
S4—Cu36A—Cu27	58.27 (15)	Cu48—S25—Cu54A	133.7 (2)
S8—Cu36A—Cu27	62.29 (15)	Cu61^ix^—S26—Cu49	106.7 (2)
Cu45A—Cu36A—Cu27	111.6 (3)	Cu61^ix^—S26—Cu19	89.62 (19)
S24—Cu36A—S7	104.1 (3)	Cu49—S26—Cu19	80.68 (17)
S4—Cu36A—S7	94.41 (18)	Cu61^ix^—S26—Cu50	130.2 (2)
S8—Cu36A—S7	88.7 (2)	Cu49—S26—Cu50	116.3 (2)
Cu45A—Cu36A—S7	119.4 (3)	Cu19—S26—Cu50	74.48 (16)
Cu27—Cu36A—S7	53.78 (13)	Cu61^ix^—S26—Cu47B	117.8 (4)
S24—Cu36A—Cu15	52.55 (14)	Cu49—S26—Cu47B	79.8 (4)
S4—Cu36A—Cu15	104.4 (3)	Cu19—S26—Cu47B	149.9 (4)
S8—Cu36A—Cu15	101.14 (18)	Cu50—S26—Cu47B	94.1 (4)
Cu45A—Cu36A—Cu15	64.0 (2)	Cu61^ix^—S26—Cu47A	121.4 (3)
Cu27—Cu36A—Cu15	149.3 (2)	Cu49—S26—Cu47A	92.6 (3)
S7—Cu36A—Cu15	156.5 (2)	Cu19—S26—Cu47A	148.8 (4)
S24—Cu36A—Cu37	99.59 (18)	Cu50—S26—Cu47A	81.6 (3)
S4—Cu36A—Cu37	93.8 (2)	Cu61^ix^—S26—Cu55	62.90 (15)
S8—Cu36A—Cu37	48.33 (14)	Cu49—S26—Cu55	119.3 (2)
Cu45A—Cu36A—Cu37	99.5 (3)	Cu19—S26—Cu55	149.0 (2)
Cu27—Cu36A—Cu37	97.07 (13)	Cu50—S26—Cu55	111.34 (19)
S7—Cu36A—Cu37	137.1 (2)	Cu47B—S26—Cu55	61.1 (4)
Cu15—Cu36A—Cu37	56.44 (11)	Cu47A—S26—Cu55	59.4 (3)
S24—Cu36B—S8	132.6 (10)	Cu58—S27—Cu56	116.10 (18)
S24—Cu36B—S7	119.5 (10)	Cu58—S27—Cu38	112.53 (19)
S8—Cu36B—S7	103.1 (8)	Cu56—S27—Cu38	114.12 (19)
S24—Cu36B—Cu27	158.9 (13)	Cu58—S27—Cu17	74.40 (15)
S8—Cu36B—Cu27	63.5 (4)	Cu56—S27—Cu17	75.96 (15)
S7—Cu36B—Cu27	57.1 (4)	Cu38—S27—Cu17	77.34 (15)
S24—Cu36B—Cu17	51.4 (5)	Cu58—S27—Cu50	130.9 (2)
S8—Cu36B—Cu17	104.5 (10)	Cu56—S27—Cu50	102.52 (19)
S7—Cu36B—Cu17	99.7 (10)	Cu38—S27—Cu50	73.98 (14)
Cu27—Cu36B—Cu17	146.3 (12)	Cu17—S27—Cu50	147.7 (2)
S24—Cu36B—Cu39	104.8 (9)	Cu58—S27—Cu57	73.38 (15)
S8—Cu36B—Cu39	50.1 (5)	Cu56—S27—Cu57	101.52 (18)
S7—Cu36B—Cu39	92.9 (9)	Cu38—S27—Cu57	133.6 (2)
Cu27—Cu36B—Cu39	96.2 (7)	Cu17—S27—Cu57	142.4 (2)
Cu17—Cu36B—Cu39	57.9 (6)	Cu50—S27—Cu57	69.96 (14)
S24—Cu36B—Cu45B	71.6 (9)	Cu20—S28—Cu16	124.8 (2)
S8—Cu36B—Cu45B	124.9 (13)	Cu20—S28—Cu59^viii^	93.40 (18)
S7—Cu36B—Cu45B	97.4 (12)	Cu16—S28—Cu59^viii^	80.56 (17)
Cu27—Cu36B—Cu45B	87.8 (9)	Cu20—S28—Cu39	100.23 (19)
Cu17—Cu36B—Cu45B	121.6 (8)	Cu16—S28—Cu39	95.68 (18)
Cu39—Cu36B—Cu45B	169.5 (13)	Cu59^viii^—S28—Cu39	165.4 (2)
S8—Cu37—S21^v^	129.21 (19)	Cu20—S28—Cu17	117.0 (2)
S8—Cu37—S1	113.83 (17)	Cu16—S28—Cu17	118.1 (2)
S21^v^—Cu37—S1	115.94 (18)	Cu59^viii^—S28—Cu17	93.80 (18)
S8—Cu37—Cu4	53.49 (13)	Cu39—S28—Cu17	75.44 (16)
S21^v^—Cu37—Cu4	107.50 (16)	Cu20—S28—Cu48^v^	67.34 (15)
S1—Cu37—Cu4	118.77 (14)	Cu16—S28—Cu48^v^	64.40 (14)
S8—Cu37—Cu15	105.95 (15)	Cu59^viii^—S28—Cu48^v^	111.19 (19)
S21^v^—Cu37—Cu15	53.88 (13)	Cu39—S28—Cu48^v^	79.20 (15)
S1—Cu37—Cu15	102.42 (14)	Cu17—S28—Cu48^v^	154.6 (2)
Cu4—Cu37—Cu15	138.40 (11)	Cu20—S29—Cu57^viii^	95.27 (17)
S8—Cu37—Cu36A	51.75 (17)	Cu20—S29—Cu18^v^	126.2 (2)
S21^v^—Cu37—Cu36A	105.76 (15)	Cu57^viii^—S29—Cu18^v^	75.36 (15)
S1—Cu37—Cu36A	103.9 (2)	Cu20—S29—Cu40^v^	107.43 (18)
Cu4—Cu37—Cu36A	103.35 (17)	Cu57^viii^—S29—Cu40^v^	157.1 (2)
Cu15—Cu37—Cu36A	58.19 (10)	Cu18^v^—S29—Cu40^v^	93.02 (17)
S8—Cu37—Cu3^v^	124.60 (14)	Cu20—S29—Cu10	118.4 (2)
S21^v^—Cu37—Cu3^v^	95.61 (14)	Cu57^viii^—S29—Cu10	96.12 (17)
S1—Cu37—Cu3^v^	49.78 (12)	Cu18^v^—S29—Cu10	115.2 (2)
Cu4—Cu37—Cu3^v^	86.38 (9)	Cu40^v^—S29—Cu10	70.70 (14)
Cu15—Cu37—Cu3^v^	128.22 (10)	Cu20—S29—Cu48^v^	68.99 (14)
Cu36A—Cu37—Cu3^v^	152.25 (16)	Cu57^viii^—S29—Cu48^v^	110.05 (19)
S27—Cu38—S7	119.44 (17)	Cu18^v^—S29—Cu48^v^	65.55 (14)
S27—Cu38—S12	121.20 (17)	Cu40^v^—S29—Cu48^v^	81.71 (14)
S7—Cu38—S12	117.62 (17)	Cu10—S29—Cu48^v^	152.40 (19)
S27—Cu38—Cu50	53.43 (12)	Cu18—S30—Cu41	89.29 (17)
S7—Cu38—Cu50	103.71 (14)	Cu18—S30—Cu19	122.6 (2)
S12—Cu38—Cu50	122.91 (15)	Cu41—S30—Cu19	102.53 (18)
S27—Cu38—Cu39	104.01 (14)	Cu18—S30—Cu11	114.2 (2)
S7—Cu38—Cu39	98.81 (14)	Cu41—S30—Cu11	75.47 (16)
S12—Cu38—Cu39	53.72 (13)	Cu19—S30—Cu11	123.1 (2)
Cu50—Cu38—Cu39	154.11 (11)	Cu18—S30—Cu58^ix^	76.49 (16)
S27—Cu38—Cu6	104.41 (14)	Cu41—S30—Cu58^ix^	155.0 (2)
S7—Cu38—Cu6	52.24 (12)	Cu19—S30—Cu58^ix^	102.45 (18)
S12—Cu38—Cu6	120.94 (14)	Cu11—S30—Cu58^ix^	91.56 (17)
Cu50—Cu38—Cu6	58.74 (8)	Cu42—S31—Cu59	105.7 (2)
Cu39—Cu38—Cu6	147.11 (11)	Cu42—S31—Cu57	120.4 (2)
S27—Cu38—Cu17	51.52 (12)	Cu59—S31—Cu57	117.27 (19)
S7—Cu38—Cu17	100.46 (14)	Cu42—S31—Cu19	75.93 (16)
S12—Cu38—Cu17	105.39 (14)	Cu59—S31—Cu19	78.43 (16)
Cu50—Cu38—Cu17	103.67 (9)	Cu57—S31—Cu19	74.61 (15)
Cu39—Cu38—Cu17	59.22 (8)	Cu42—S31—Cu51B	63.7 (3)
Cu6—Cu38—Cu17	132.94 (10)	Cu59—S31—Cu51B	108.6 (3)
S27—Cu38—Cu9	101.61 (13)	Cu57—S31—Cu51B	128.4 (3)
S7—Cu38—Cu9	125.93 (14)	Cu19—S31—Cu51B	139.5 (3)
S12—Cu38—Cu9	51.59 (12)	Cu42—S31—Cu60A	132.4 (2)
Cu50—Cu38—Cu9	72.77 (9)	Cu59—S31—Cu60A	102.8 (2)
Cu39—Cu38—Cu9	103.91 (10)	Cu57—S31—Cu60A	76.5 (2)
Cu6—Cu38—Cu9	86.19 (9)	Cu19—S31—Cu60A	147.7 (2)
Cu17—Cu38—Cu9	133.33 (10)	Cu42—S31—Cu60B	128.5 (3)
S8—Cu39—S28	120.67 (19)	Cu59—S31—Cu60B	89.0 (4)
S8—Cu39—S12	116.84 (18)	Cu57—S31—Cu60B	92.9 (4)
S28—Cu39—S12	121.95 (18)	Cu19—S31—Cu60B	155.2 (3)
S8—Cu39—Cu30	81.64 (14)	Cu51B—S31—Cu60B	64.8 (4)
S28—Cu39—Cu30	140.79 (18)	Cu42—S31—Cu51A	69.3 (2)
S12—Cu39—Cu30	54.40 (12)	Cu59—S31—Cu51A	122.4 (3)
S8—Cu39—Cu38	101.58 (15)	Cu57—S31—Cu51A	112.8 (3)
S28—Cu39—Cu38	107.02 (15)	Cu19—S31—Cu51A	143.0 (2)
S12—Cu39—Cu38	52.65 (12)	Cu60A—S31—Cu51A	63.3 (2)
Cu30—Cu39—Cu38	98.09 (10)	Cu12—S32—Cu43	89.6 (2)
S8—Cu39—Cu17	104.00 (15)	Cu12—S32—Cu61	60.2 (2)
S28—Cu39—Cu17	52.32 (13)	Cu43—S32—Cu61	115.3 (2)
S12—Cu39—Cu17	106.93 (14)	Cu12—S32—Cu60A	75.9 (3)
Cu30—Cu39—Cu17	159.92 (12)	Cu43—S32—Cu60A	134.9 (2)
Cu38—Cu39—Cu17	62.02 (8)	Cu61—S32—Cu60A	94.2 (2)
S8—Cu39—Cu4	51.74 (13)	Cu12—S32—Cu20	121.9 (3)
S28—Cu39—Cu4	99.76 (15)	Cu43—S32—Cu20	79.38 (16)
S12—Cu39—Cu4	121.86 (14)	Cu61—S32—Cu20	73.76 (18)
Cu30—Cu39—Cu4	67.56 (9)	Cu60A—S32—Cu20	144.1 (3)
Cu38—Cu39—Cu4	149.96 (12)	Cu12—S32—Cu58	153.6 (3)
Cu17—Cu39—Cu4	131.04 (11)	Cu43—S32—Cu58	114.3 (2)
S8—Cu39—Cu36B	48.2 (5)	Cu61—S32—Cu58	114.2 (2)
S28—Cu39—Cu36B	100.4 (4)	Cu60A—S32—Cu58	79.0 (2)
S12—Cu39—Cu36B	111.8 (5)	Cu20—S32—Cu58	75.85 (16)
Cu30—Cu39—Cu36B	117.4 (3)	Cu12—S32—Cu51A	59.9 (3)
Cu38—Cu39—Cu36B	66.0 (5)	Cu43—S32—Cu51A	69.4 (2)
Cu17—Cu39—Cu36B	59.2 (4)	Cu61—S32—Cu51A	119.9 (3)
Cu4—Cu39—Cu36B	96.3 (5)	Cu60A—S32—Cu51A	66.4 (2)
S13—Cu40—S29^iii^	124.08 (17)	Cu20—S32—Cu51A	148.8 (3)
S13—Cu40—S16^iii^	121.74 (16)	Cu58—S32—Cu51A	116.3 (3)
S29^iii^—Cu40—S16^iii^	114.18 (17)	Cu12—S32—Cu60B	62.6 (4)
S13—Cu40—Cu7	55.65 (12)	Cu43—S32—Cu60B	127.7 (3)
S29^iii^—Cu40—Cu7	99.08 (14)	Cu61—S32—Cu60B	89.3 (3)
S16^iii^—Cu40—Cu7	116.37 (13)	Cu20—S32—Cu60B	152.7 (3)
S13—Cu40—Cu34^xv^	106.48 (14)	Cu58—S32—Cu60B	92.6 (3)
S29^iii^—Cu40—Cu34^xv^	106.51 (14)		

Symmetry codes: (i) *x*, *y*, *z*−1; (ii) −*x*, −*y*+1, −*z*; (iii) *x*, *y*−1, *z*; (iv) −*x*, −*y*+1, −*z*+1; (v) *x*, *y*+1, *z*; (vi) *x*, *y*, *z*+1; (vii) *x*, *y*+1, *z*+1; (viii) −*x*+1/2, *y*+1/2, −*z*+3/2; (ix) −*x*+1/2, *y*−1/2, −*z*+3/2; (x) −*x*+1/2, *y*+1/2, −*z*+1/2; (xi) −*x*, −*y*+2, −*z*+1; (xii) *x*, *y*+1, *z*−1; (xiii) −*x*+1/2, *y*−1/2, −*z*+1/2; (xiv) *x*, *y*−1, *z*−1; (xv) *x*, *y*−1, *z*+1.
