# Supplementary material for: Natural djurleite with refined composition Cu61.39S32 revealing disorder of some Cu sites
Source: IUCrdata. 2022 Jul 12;7(Pt 7):x220694. doi: 10.1107/S2414314622006940 (PMC9635409; doi:10.1107/S2414314622006940)
Supplement: Supplementary file 3 [file x-07-x220694-sup3.docx]

**SUPPLEMENTARY MATERIALS:**

**Natural djurleite with refined composition Cu_61.39_S_32_ revealing disorder of some Cu sites**

**Yawei Zhou, Changzeng Fan*, Bin Wen and Lifeng Zhang**

State Key Laboratory of Metastable Materials Science and Technology, Yanshan University,

Qinhuangdao 066004, P.R. China

*Correspondence email: [chzfan@ysu.edu.cn](mailto:chzfan@ysu.edu.cn)

The chemical compositions were examined quantitatively by energy dispersive X-ray spectroscopy (EDX) analysis attached to a Hitachi S-3400N SEM for the purpose of guiding the crystal structure refinement. The examined points are designated in Fig. S1, and the corresponding results are listed in Table S1. The deviation relative to the results of refinement of chemical composition is probably caused by the tilt of the single crystal surface to the incident beam especially for spot 3 and selected area 5. For ease of reading, the atomic ratio of Cu and S are calculated and shown in the last column of Table S1.


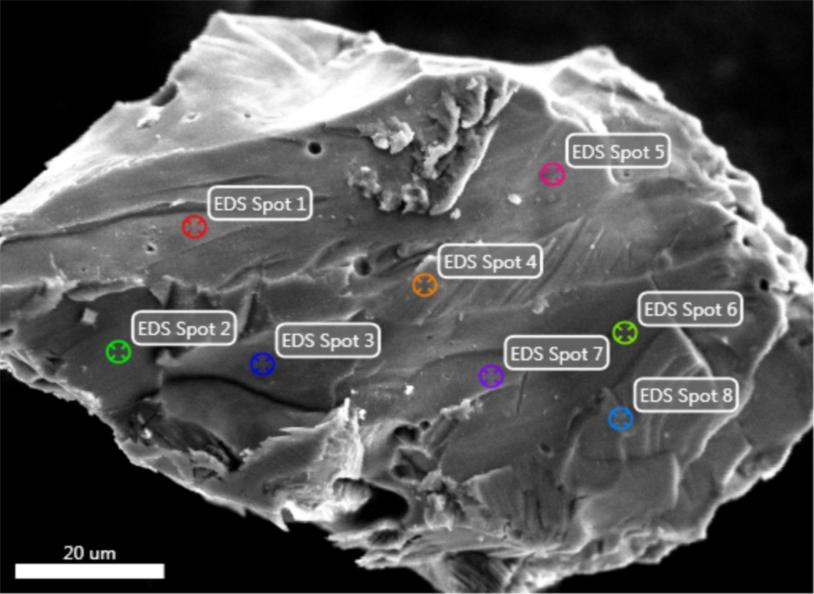

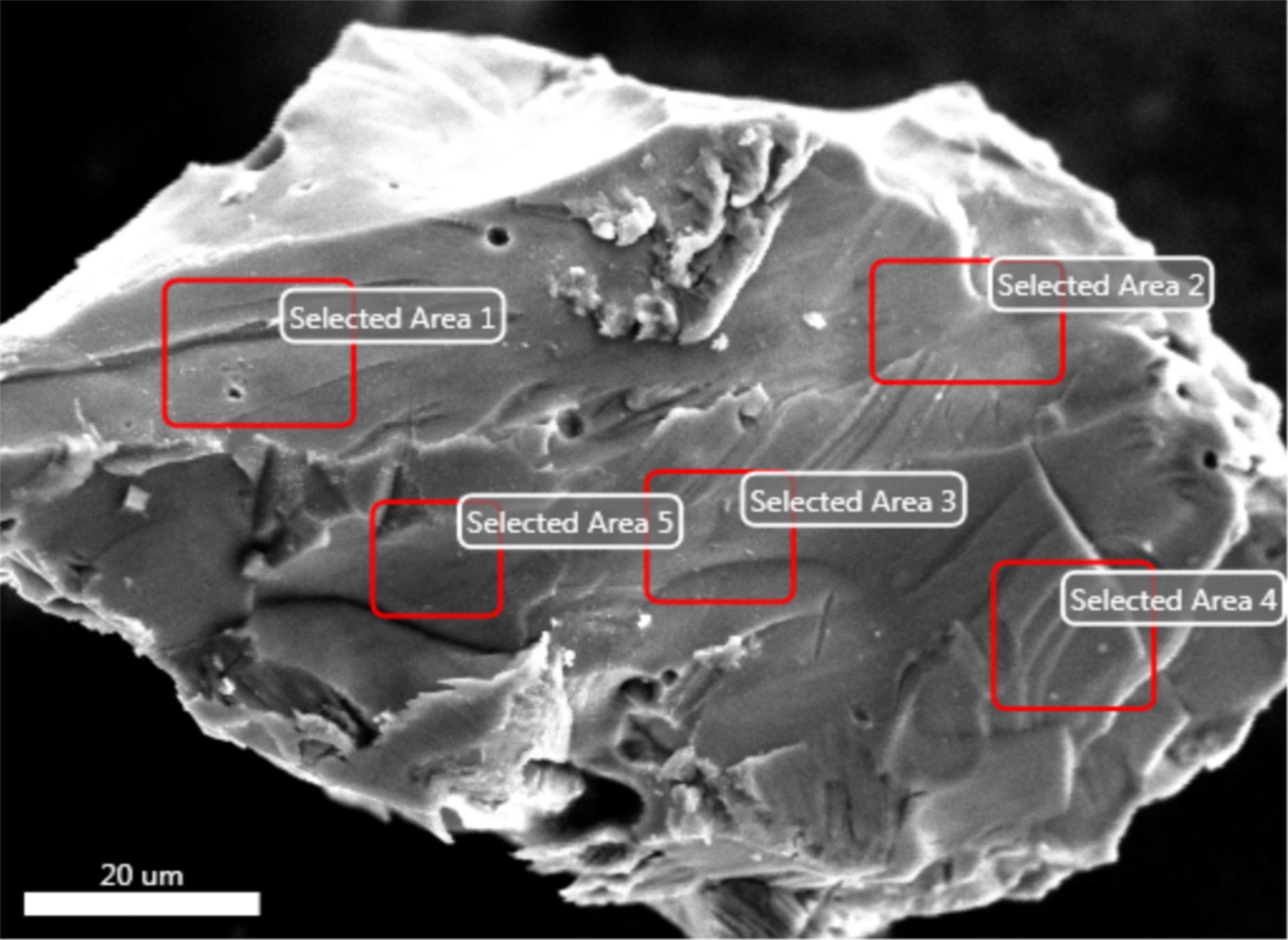


Fig. S1 Single crystal of Cu_61.39_S_32_ with selected spots and areas for EDX analysis

**Table S1 EDX results for selected points as designated in Fig. S1**

|  | Element | Weight (%) | Atomic (%) | Error (%) | Cu : S |
| --- | --- | --- | --- | --- | --- |
| Spot1 | CuK | 76.80 | 62.55 | 2.75 | 1.670:1 |
|  | SK | 23.20 | 37.45 | 5.34 |  |
| Spot2 | CuK | 87.86 | 78.51 | 2.77 | 3.653:1 |
|  | SK | 12.14 | 21.49 | 6.20 |  |
| Spot3 | CuK | 98.73 | 97.51 | 2.93 | 39.161:1 |
|  | SK | 1.27 | 2.49 | 13.68 |  |
| Spot4 | CuK | 80.03 | 66.92 | 2.82 | 2.023:1 |
|  | SK | 19.97 | 33.08 | 5.62 |  |
| Spot5 | CuK | 79.67 | 66.42 | 2.76 | 1.978:1 |
|  | SK | 20.33 | 33.58 | 5.60 |  |
| Spot6 | CuK | 80.28 | 67.26 | 2.82 | 2.054:1 |
|  | SK | 19.72 | 32.74 | 5.66 |  |
| Spot7 | CuK | 80.96 | 68.21 | 2.77 | 2.146:1 |
|  | SK | 19.04 | 31.79 | 5.69 |  |
| Spot8 | CuK | 80.59 | 67.69 | 2.91 | 2.095:1 |
|  | SK | 19.41 | 32.31 | 5.70 |  |
| Area1 | CuK | 76.68 | 62.39 | 2.71 | 1.659:1 |
|  | SK | 23.32 | 37.61 | 5.24 |  |
| Area2 | CuK | 78.08 | 64.25 | 2.68 | 1.797:1 |
|  | SK | 21.92 | 35.75 | 5.38 |  |
| Area3 | CuK | 85.86 | 75.40 | 2.72 | 3.065:1 |
|  | SK | 14.14 | 24.60 | 5.97 |  |
| Area4 | CuK | 83.72 | 72.18 | 2.81 | 2.595:1 |
|  | SK | 16.28 | 27.82 | 5.85 |  |
| Area5 | CuK | 90.69 | 83.09 | 2.76 | 4.914:1 |
|  | SK | 9.31 | 16.91 | 6.57 |  |
